# *In Situ* Observation of Elusive Dirhodium Carbenes and Studies on the Innate Role of Carboxamidate Ligands in Dirhodium Paddlewheel Complexes: A Combined Experimental and Computational Approach

**DOI:** 10.1021/jacs.4c09847

**Published:** 2024-09-11

**Authors:** Matthias Peeters, Lorenzo Baldinelli, Markus Leutzsch, Fabio Caló, Alexander A. Auer, Giovanni Bistoni, Alois Fürstner

**Affiliations:** † Max-Planck-Institut für Kohlenforschung, Mülheim/Ruhr D-45470, Germany; ‡ Department of Chemistry, Biology and Biotechnology, University of Perugia, Via Elce di Sotto 8, Perugia I-06123, Italy

## Abstract

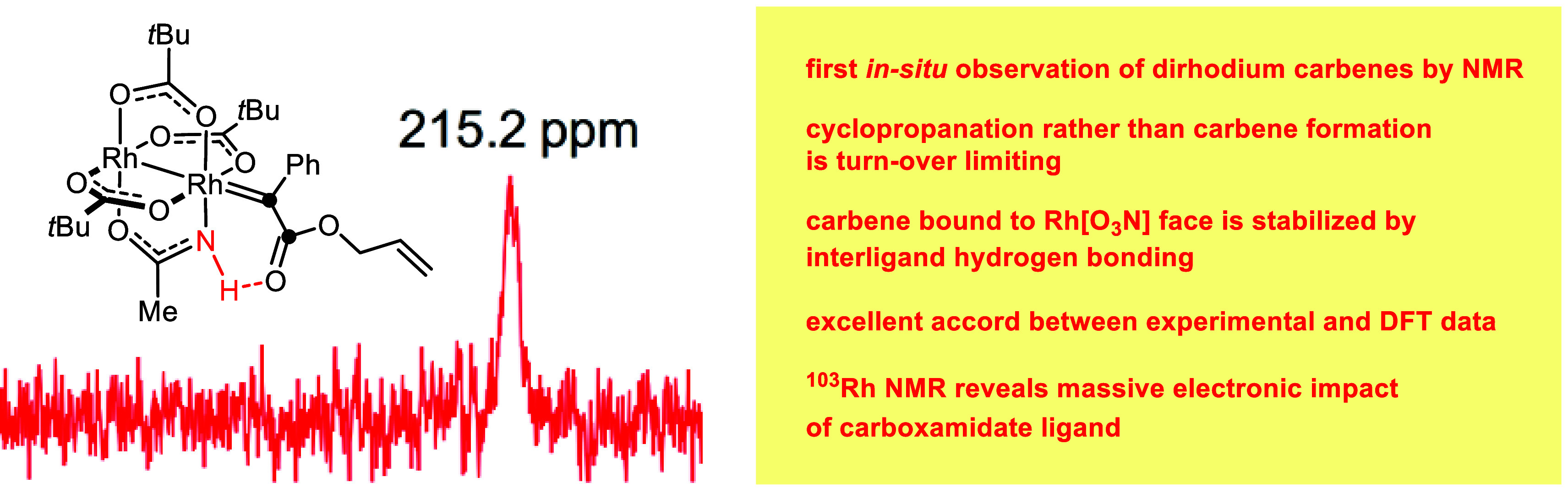

Carboxamidates as equatorial ligands in dirhodium paddlewheel catalysts are widely believed to increase selectivity at the expense of reactivity. The results of the combined experimental and computational approach described in this paper show that one has to beware of such generalizations. First, ^103^Rh NMR revealed how strongly primary carboxamidates impact the electronic nature of the rhodium center they are bound to; at the same time, such ligands stabilize donor/acceptor carbenes by engaging their ester carbonyl group into peripheral interligand hydrogen bonding. This array benefits selectivity as well as reactivity if maintained along the entire reaction coordinate of a catalytic cyclopropanation. In settings where the hydrogen bond needs to be distorted for the reaction to proceed, however, it constitutes a significant enthalpic handicap. Representative examples for each scenario were analyzed by DFT; in both cases, the cyclopropanation step rather than carbene formation was found to be turnover-limiting. While this conclusion somehow contradicts the literature, it implied that the direct observation of highly reactive dirhodium carbenes in truly catalytic settings might be possible, even though the intermediates carry olefinic sites amenable to intramolecular cyclopropanation. Such *in situ* monitoring by NMR is without precedent, yet it was successful with the homoleptic catalyst [Rh_2_(OPiv)_4_] as well as with its heteroleptic sibling [Rh_2_(OPiv)_3_(acam)] comprising an acetamidate (acam); in the latter case, the carbene bound to the rhodium atom at the [O_3_N]-face was observed, which concurs with the computational data that this species is stabilized by the forecited interligand hydrogen bonding.

## Introduction

Catalytic transformations effected by dirhodium(II) “paddlewheel” complexes take place at an axial coordination site where ligand exchange is fast and facile. In contrast, the anionic μ-bridging equatorial “paddlewheel” ligands are tightly bound and impart high stability onto such catalysts; although not limited to, carboxylates and carboxamidates dominate the field.^1,2^ For this unique combination of effects, (chiral) dirhodium(II) paddlewheel complexes are highly enabling; they excel in different types of catalytic transformations ranging from asymmetric Lewis acid catalysis to oxidation reactions.^3^ Of arguably highest significance, however, is their widespread use in metal carbene and metal nitrene chemistry.^4−21^

Somewhat surprisingly, the exceptional level of sophistication in the field is juxtaposed by a still rather limited understanding of some fundamental features of these popular catalysts and the derived reactive intermediates. For example, dirhodium carboxamidates are commonly viewed as (notably) less active but more selective than dirhodium carboxylates.^22−29^ The stronger donor capacity of amides is usually cited in this context, which is supposed to entail higher electron density at rhodium and, as a consequence, increased electron back-donation from the metal into the carbene (nitrene) center.^11,30−33^ While this seems to provide a rather intuitive explanation for potentially lower reactivity of these “superelectrophilic” species, the supposed correlation between back-bonding and chemo-, regio- and stereoselectivity is less apparent. Apart from a general reference to the Hammond postulate as a conceptual link between the stabilization of a reactive intermediate and the observed selectivity, little other reasoning can be found in the literature.^32,34,35^

**Scheme 1:**
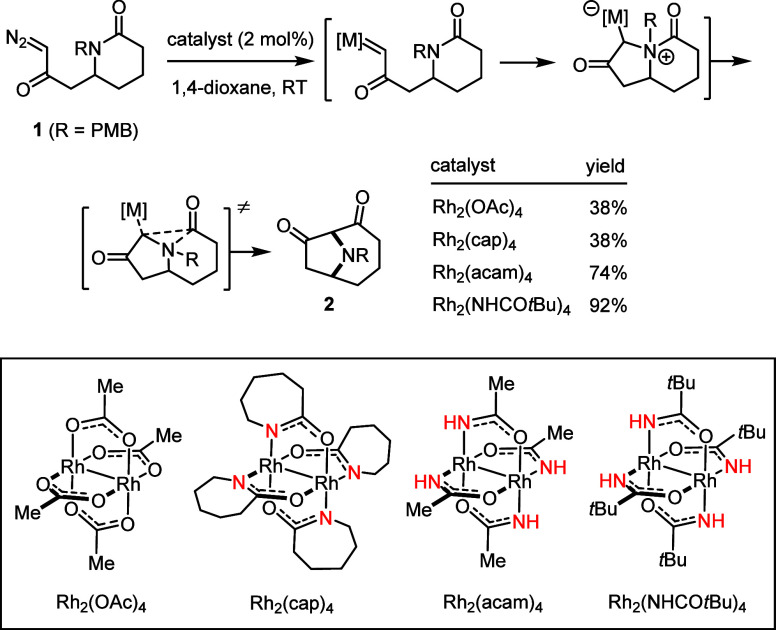
Comparison of Different Homoleptic Dirhodium Catalysts in the Formation of a Nitrogen-Bridged Bicycle via Metal Carbene and Ylide Formation, Followed by a Stevens-Type Rearrangement^36^

On closer inspection, one can hardly overlook that the generalization that carboxamidates increase selectivity and/or efficiency at the expense of reactivity is not correct. An example reported by Nemoto, Hamada and co-workers may suffice to illustrate the point (Scheme 1):^36^ the formation of the nitrogen-bridged bicycle **2** from diazoketone **1** is thought to commence with the attack of the amide N atom onto the rhodium carbene primarily formed, followed by a Stevens-type [1,2] acyl shift to give the final product. [Rh_2_(OAc)_4_] and the standard tetracarboxamidate complex [Rh_2_(cap)_4_] perform equally poorly, whereas [Rh_2_(acam)_4_] or [Rh_2_(NHC(O)*t*Bu)_4_] both led to a much better yield. Whether the catalyst comprises a carboxylate or carboxamidate is apparently not the decisive factor; rather the specific type of carboxamidate (primary versus secondary) seems to be of critical importance. Transient hydrogen bonding of the reactive intermediates was tentatively invoked to explain the results.^36−38^

## Results and Discussion

### Stannylated Cyclopropanes

**Scheme 2:**
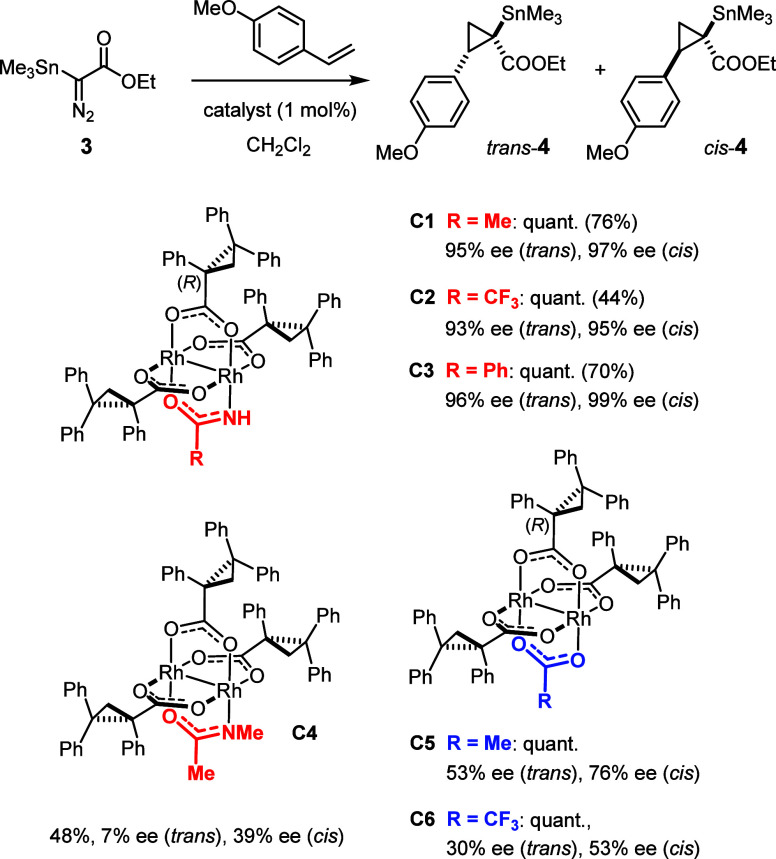
Comparison of the Performance of Different Heteroleptic Dirhodium Catalysts in Asymmetric Cyclopropanation Using α-Trimethylstannyl α-Diazoacetate **3** In all cases, the diastereoselectivity was ∼1:1; the conversions were quantitative after a 6 h reaction time according to ^1^H NMR (isolated yields in brackets) except for **C4** as the catalyst.

As part of our studies into new concepts for metal carbene chemistry,^39−42^ we reached a similar conclusion while studying the asymmetric cyclopropanation of olefins with α-trimethylstannyl α-diazoacetate **3** (Scheme 2).^43,44^*Heteroleptic* dirhodium catalysts^45^ comprising three chiral carboxylate ligands and one achiral acetamidate turned out to be uniquely effective and selective in this case. While replacement of the acetamidate in **C1** by trifluoroacetamidate (**C2**) or benzamidate (**C3**) was largely inconsequential, methylation of the N atom as found in **C4** proved detrimental for both yield and selectivity. Likewise, the ee dropped massively when the acetamidate was formally replaced by acetate (**C5**) or trifluoroacetate (**C6**). High enantioselectivity hence mandated a protic ligand.^43,44^

**Figure 1:**
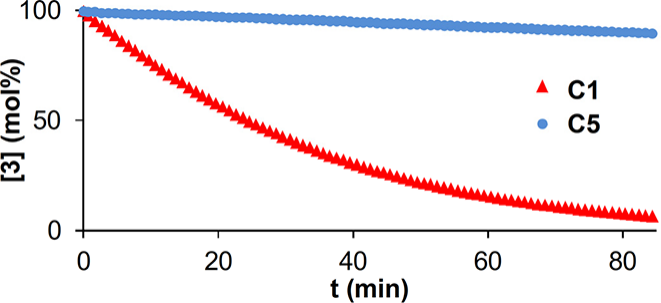
Consumption (^1^H NMR) of the stannylated diazoester **3** during the cyclopropanation of *p*-methoxystyrene catalyzed by two different heteroleptic complexes (1 mol%) in CD_2_Cl_2_ at 0 °C.

Under this proviso, the reaction must occur preferentially or even exclusively at the Rh-center ligated to the N atom (Rh[O_3_N]), which contradicts the general perception that amidates entail lower reactivity. This startling conclusion was corroborated by kinetic data:^44^ while **3** was rapidly consumed and product **4** formed in high yield with **C1** as the catalyst, the analogous acetate-containing complex **C5** reacted much more slowly (and less selectively) (Figure 1). Since both catalysts are heteroleptic and essentially isosteric (the −NH group in **C1** and the corresponding O atom in **C5** are of similar size), it seems that the primary amidate *upregulates* reactivity and selectivity alike.

**Figure 2:**
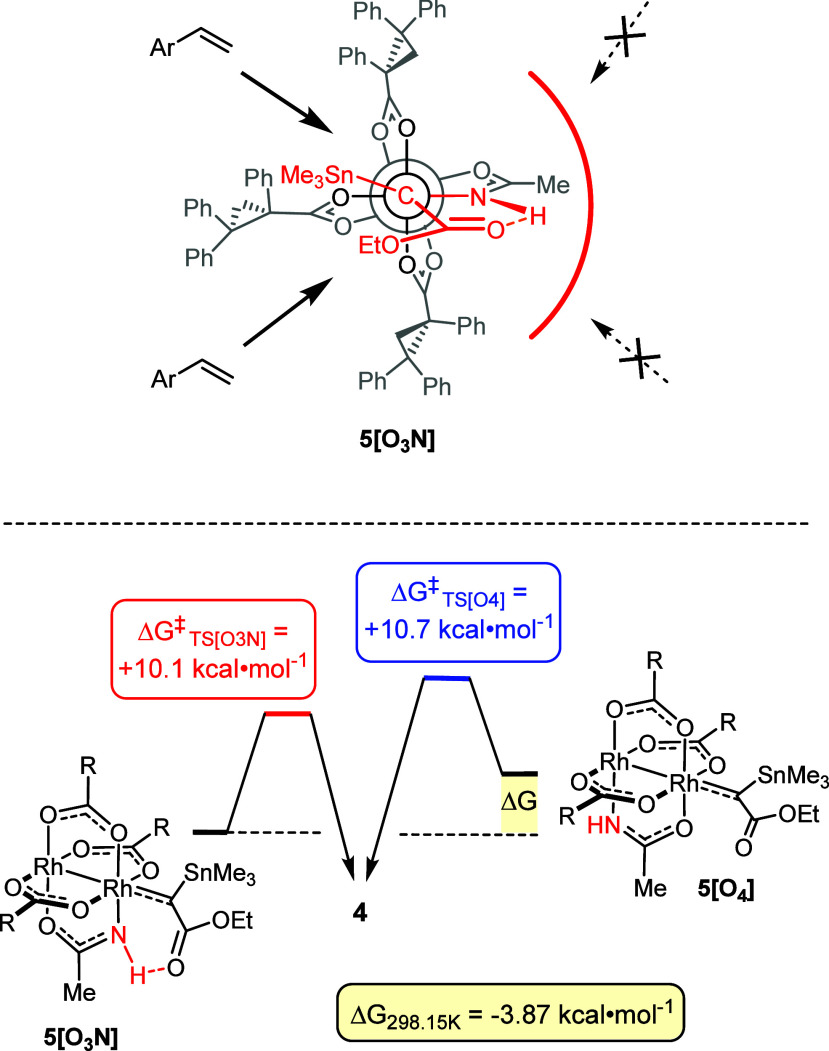
Top: Newman projection along the C(carbene)−Rh−Rh axis of the stannylated carbene **5[O**_**3**_**N]** benefiting from interligand hydrogen bonding of the ester carbonyl group to the –NH group of the adjacent acetamidate ligand; the arrows show the viable and the blocked trajectories for the incoming reaction partner; bottom: computed barriers for cyclopropanation of *p*-methoxystyrene by the isomeric carbenes **5[O**_**3**_**N]** and **5[O**_**4**_**]** bound to the [O_3_N] and the [O_4_] faces of the heteroleptic catalyst **C1**, respectively.

A detailed computational analysis allowed us to shed light into this apparent paradox. It was found that the protic –NH substituent engages the carbonyl group of the rhodium carbene intermediate **5[O**_**3**_**N]** derived from **C1** in short (∼2.37 Å) interligand hydrogen bonding (Figure 2, top).^44,46^ In order not to disrupt this stabilizing interaction, the incoming alkene approaches the highly electrophilic carbene center from the “backside”, opposite to the –NH function. These trajectories translate into tight and well-ordered transition states, which allow the absolute configuration of the resulting diastereomeric stannylated cyclopropanes *trans*-**4** and *cis*-**4** to be rationalized (dr ≈ 1:1; ee (*trans*) = 95%, ee (*cis*) = 97%). The validity of this model was affirmed by the fact that it also guided a successful search for “second generation” catalysts with greatly improved diastereoselectivity.^44^

While this first round of analysis had been focused on the selectivity issues, the question as to why the reaction does not occur at the [O_4_]-face of the dirhodium catalyst **C1** remained unanswered. Therefore, we now complement the picture with computational data for the isomeric carbene **5[O**_**4**_**]** (Figure 2, bottom). For the lack of a stabilizing peripheral H-bond, this species is notably less stable (Δ*G*(**5**[O_3_N]-**5**[O_4_])_298.15K_ = −3.87 kcal·mol^–1^); in addition, the barrier for the cyclopropanation of 4-methoxystyrene as the model substrate is slightly higher than that for **5[O**_**3**_**N]** (ΔΔ*G*^⧧^_298.15K_ = −0.6 kcal·mol^–1^). The carbene benefiting from hydrogen bonding to the equatorial acetamidate ligand is hence thermodynamically favored and kinetically more competent at the same time. Taken together, the experimental evidence and computational data indicate that the formation of the stannylated cyclopropanes **4** catalyzed by **C1** proceeds preferentialy, if not even exclusively, at the [O_3_N]-face of the heteroleptic complex.

### Extrapolations

It remained to be seen, however, whether or not this conclusion can be generalized. Complex **C1** and its siblings carry elaborate chiral ligands, the sheer size of which might also play a role in determining the face at which an incoming diazo derivative as bulky as **3** will react; it needed to be checked if the preference remains the same in catalysts endowed with smaller equatorial ligands. Another aspect concerns the olefinic reaction partner: as mentioned above, styrene approaches the carbene intermediate **5[O**_**3**_**N]** from the backside, away from the −NH group, to preclude distortion of the interligand hydrogen bonding array with the ester carbonyl (Figure 2).^44^ It is likely that such a pathway is geometrically precluded in *intramolecular* settings, if the tether between the reacting sites is short.

To address these questions, the kinetic profiles of the intramolecular cyclopropanation of the allyl ester **6** with formation of lactone **7** were recorded by ^1^H NMR using different catalysts. Once again, the very bulky heteroleptic amidate complex **C1** resulted even in a ∼36 times faster consumption of the substrate/formation of the product than the isosteric complex **C5** bearing an acetate (for the data, see the Supporting Information); this observation suggests that the catalytic reaction proceeded largely at the [O_3_N]-face of **C1** in analogy to the previous case.

**Scheme 3:**
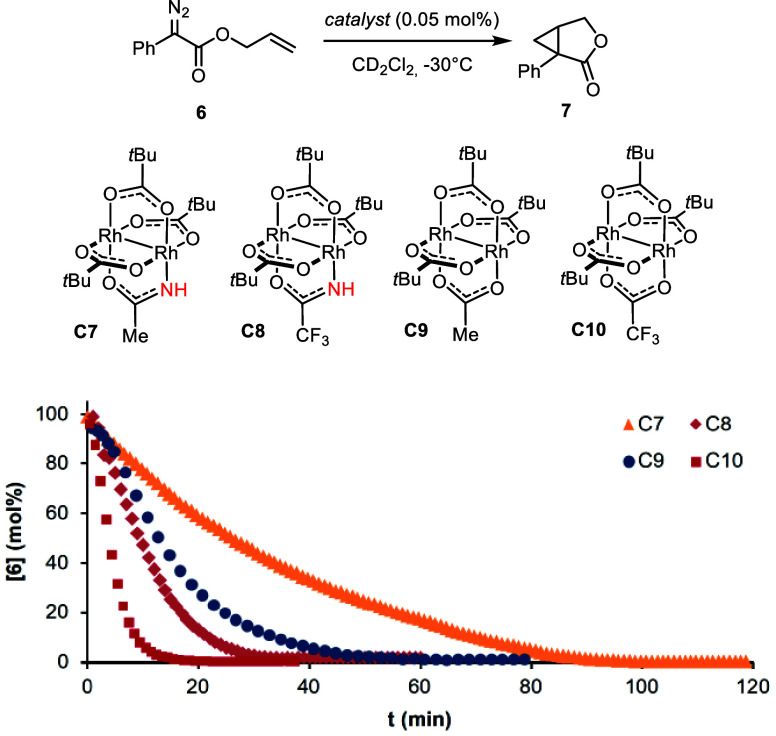
Intramolecular Cyclopropanation with Formation of **7** Catalyzed by Different Heteroleptic Complexes The kinetic profiles show the consumption of the starting diazoester **6** as monitored by ^1^H NMR.

Strikingly, however, the catalyst ranking changed completely when the sterically less encumbered achiral heteroleptic complexes **C7**–**C10** were tested (Scheme 3):^47,48^ thus, **C9** and **C10** comprising an acetate or trifluoroacetate outcompeted **C7** bearing the acetamide by factors of ∼2 and ∼7, respectively. Because these data are at odds with the results described above for the intermolecular cyclopropanation leading to **4**, additional experimental and computational data were needed in search of a more consistent overall picture.

### ^103^Rh NMR

Using a modified triple resonance experiment, our group had recently shown that high quality ^103^Rh NMR spectra of dirhodium paddlewheel catalysts can be obtained in short acquisition times, which had been basically inaccessible before.^49^ As this technique provides an entirely new opportunity to study the electronic nature of such complexes,^48^ the ^103^Rh shifts of complexes **C7**−**C10** were recorded to see if any (qualitative) correlation between the spectral data and the observed reactivity trends can be seen.

**Figure 3:**
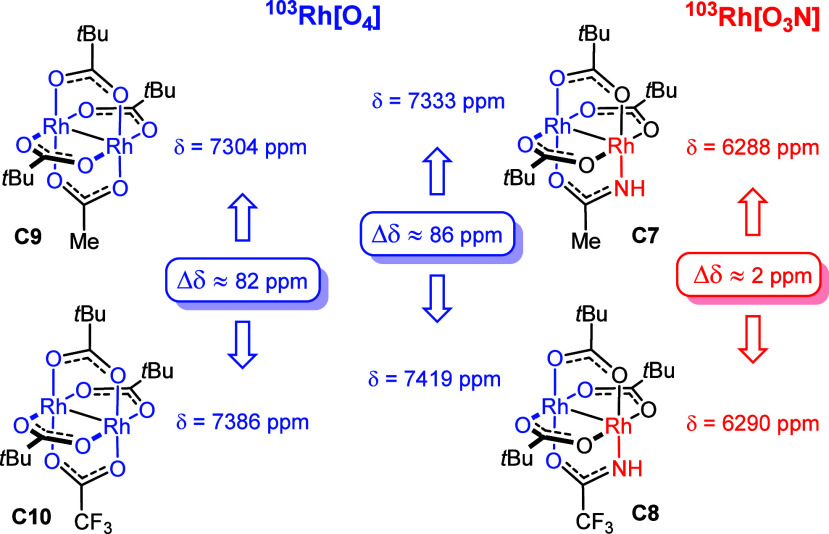
Comparison of the ^103^Rh NMR shifts (CD_3_CN) of catalysts **C7–C10**.

Some aspects are indeed noteworthy (Figure 3). First, the two rhodium centers of the heteroleptic amidate-containing complexes **C7** and **C8** have hugely different shifts, with Rh[O_3_N] being >1000 ppm more shielded than its neighbor Rh[O_4_] comprised within the [O_4_]-face; this massive differential had been observed before.^48,49^ Equally striking is the very different response of the inequivalent rhodium atoms to lateral trifluoromethylation: whereas the induced shift change at Rh[O_3_N] is negligible (Δδ ≈ 2 ppm), Rh[O_4_] is clearly affected (Δδ ≈ 86 ppm) by this chemical modification of the ligand sphere. Interestingly, the shift difference between the Rh[O_4_] sites in **C9** and its trifluoromethylated sibling **C10** is almost exactly the same (Δδ ≈ 82 ppm).

As shown in Scheme 3, the incorporation of trifluoromethylated ligands entailed notably higher reaction rates in the intramolecular cyclopropanation converting **6** into **7**; this observation is in qualitative agreement with computational data reported in the literature that trifluoromethylation of a homoleptic dirhodium paddlewheel complex lowers all barriers.^50^ Since the ^103^Rh shifts indicate that only Rh[O_4_] of **C8** “feels” the effect and gets deshielded compared to its nonfluorinated cousin **C7** whereas Rh[O_3_N] is insensitive, it seems reasonable to conclude that the formation of **7** proceeds, at least preferentially, at the [O_4_]-face of such amidate-containing heteroleptic complexes. Under this premise, catalyst **C9** comprising two Rh[O_4_] sites should be more reactive than its amidate-containing cousin **C7** (and **C10** more reactive than **C8**), which is indeed the case (Scheme 3). Despite this qualitatively coherent picture, we note as a caveat that a carbene bound to the [O_3_N]-face is likely affected by lateral trifluoromethylation in an indirect manner: the electron withdrawing group acidifies the amidate –NH group (**C7**: δ_NH_ = 4.86 ppm; **C8**: δ_NH_ = 6.47 ppm). Modulation of the interligand hydrogen bond between the protic ligand and the neighboring carbene is certainly relevant; to which extent it synergizes with the electronic effects exerted by the rhodium centers is currently unknown.

### Computational Study

In any case, it is important to note at this point that the provisional conclusions drawn above are seemingly contradictory: on the one hand, strong experimental and computational evidence is available that the *intermolecular* asymmetric cyclopropanation leading to the stannylated cyclopropanes **4** occurs at the [O_3_N]-face of the heteroleptic complex **C1**, whereas kinetic and ^103^Rh NMR spectroscopic data suggest that the *intramolecular* formation of **7** proceeds, at least preferentially, at the [O_4_]-face of catalyst **C7**. In an attempt to resolve this apparent paradox, the latter reaction was subjected to a supplementary computational study.

The computational protocol used in this work is consistent with that successfully adopted in previously published mechanistic studies on related transformations.^44,51^ Different computational settings were compared and the results found to be largely independent of the technical parameters of the calculations. The reaction energetics obtained with different basis sets, exchange correlation functionals and implicit solvation models for the reaction pathways at the [O_3_N]-face and [O_4_]-face are contained in the Supporting Information. In the end, we resorted to a preliminary exploration of the chemical space for the relevant reaction intermediates using the CREST algorithm at the XTB2 level.^52,53^ Refined geometries, frequencies and thermostatistical corrections at 298.15 K were computed using the hybrid exchange-correlation functional B3LYP-D3(BJ) together with Alrichs’ def2-SVP basis set.^54−59^ Electronic energies were refined at B3LYP-D3(BJ)/def2-TZVP(-f) level. In all calculations, solvent effects were included using the C-PCM implicit solvation model for CH_2_Cl_2_.^60,61^ The resolution of identity (RI) approximation was used in the RIJCOSX variant together with the corresponding auxiliary basis set.^62−64^ Initial guesses for the transition state (TS) structures were found with NEB and relaxed scan calculations.^65,66^ Final TS structures were then obtained through fully relaxed geometry optimizations. The low-energy stationary points were characterized by calculating vibrational modes. Transition states showed a single imaginary frequency, while reaction intermediates showed no imaginary vibrational frequency.

**Figure 4:**
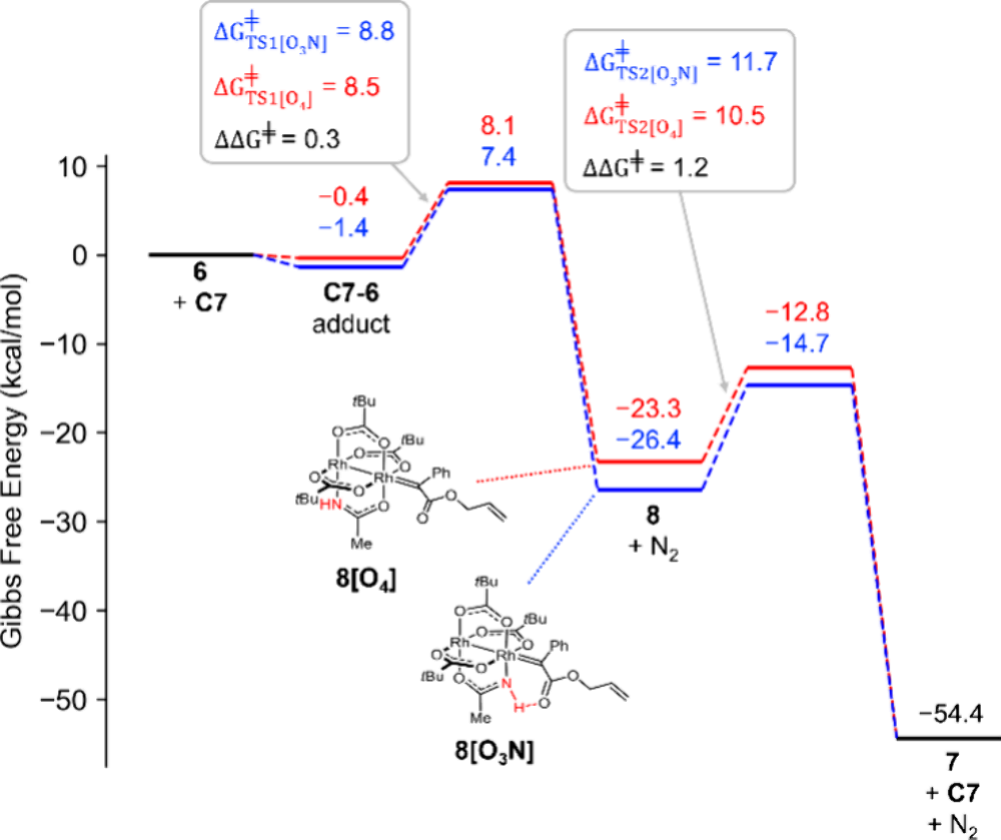
Gibbs free energy profile of the intramolecular cyclopropanation that converts **6** into **7** catalyzed by the heteroleptic dirhodium paddlewheel complex **C7**.

**Figure 5:**
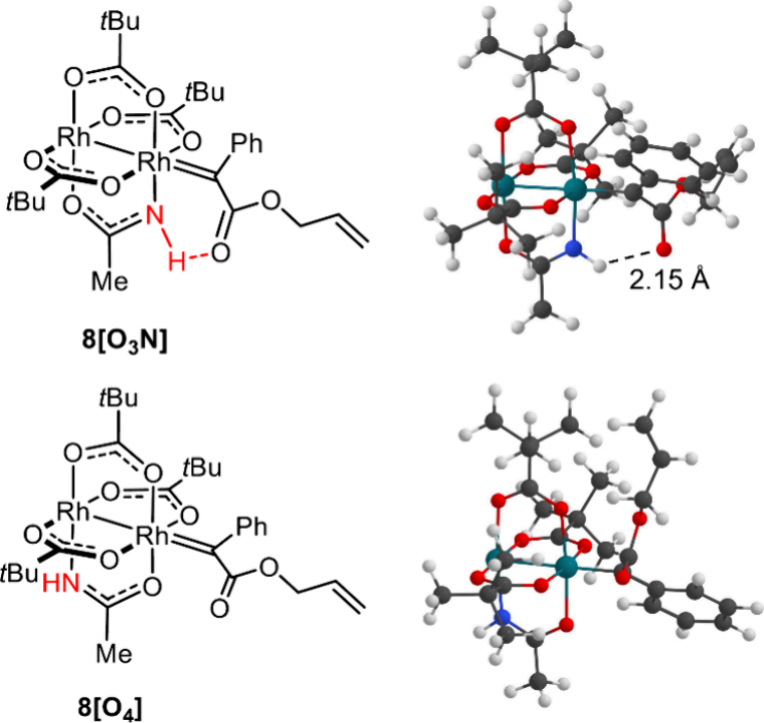
Computed structures of the isomeric dirhodium carbene intermediates **8[O**_**3**_**N]** and **8[O**_**4**_**]** derived from diazoester **6** and the heteroleptic catalyst **C7**.

The initial adduct formation upon reaction of diazoester **6** with the rhodium centers of the heteroleptic amidate complex **C7** is almost barrierless at both faces (Figure 4). While the extrusion of dinitrogen is somewhat easier at the [O_4_]-face than at the [O_3_N]-face (Δ*G*^⧧^_TS1[O4]_ = 8.5 kcal·mol^–1^; Δ*G*^⧧^_TS1[O3N]_ = 8.8 kcal·mol^–1^), only the carbene flanked by the –NH group can benefit from interligand hydrogen bonding; as a result, **8[O**_**3**_**N]** is significantly more stable than its counterpart **8[O**_**4**_**]** (ΔΔ*G* = 3.1 kcal·mol^–1^) (Figure 5). In the subsequent cyclopropane forming step, however, the stabilizing hydrogen bond gets severely distorted when the tethered olefin approaches the carbene center. Because of this enthalpic penalty, the computed barrier for the reaction at the [O_3_N]-face is higher than that at the [O_4_]-face—in contrast to what had been found for the intermolecular case, where the hydrogen bond persisted along the entire reaction coordinate (Figure 2). Since the computed differences are small (ΔΔ*G*^⧧^_TS2_ = 1.2 kcal·mol^–1^), it is likely that cyclopropane formation will proceed to some extent at both inequivalent rhodium sites.

### The Turnover-Limiting Step

**Scheme 4:**
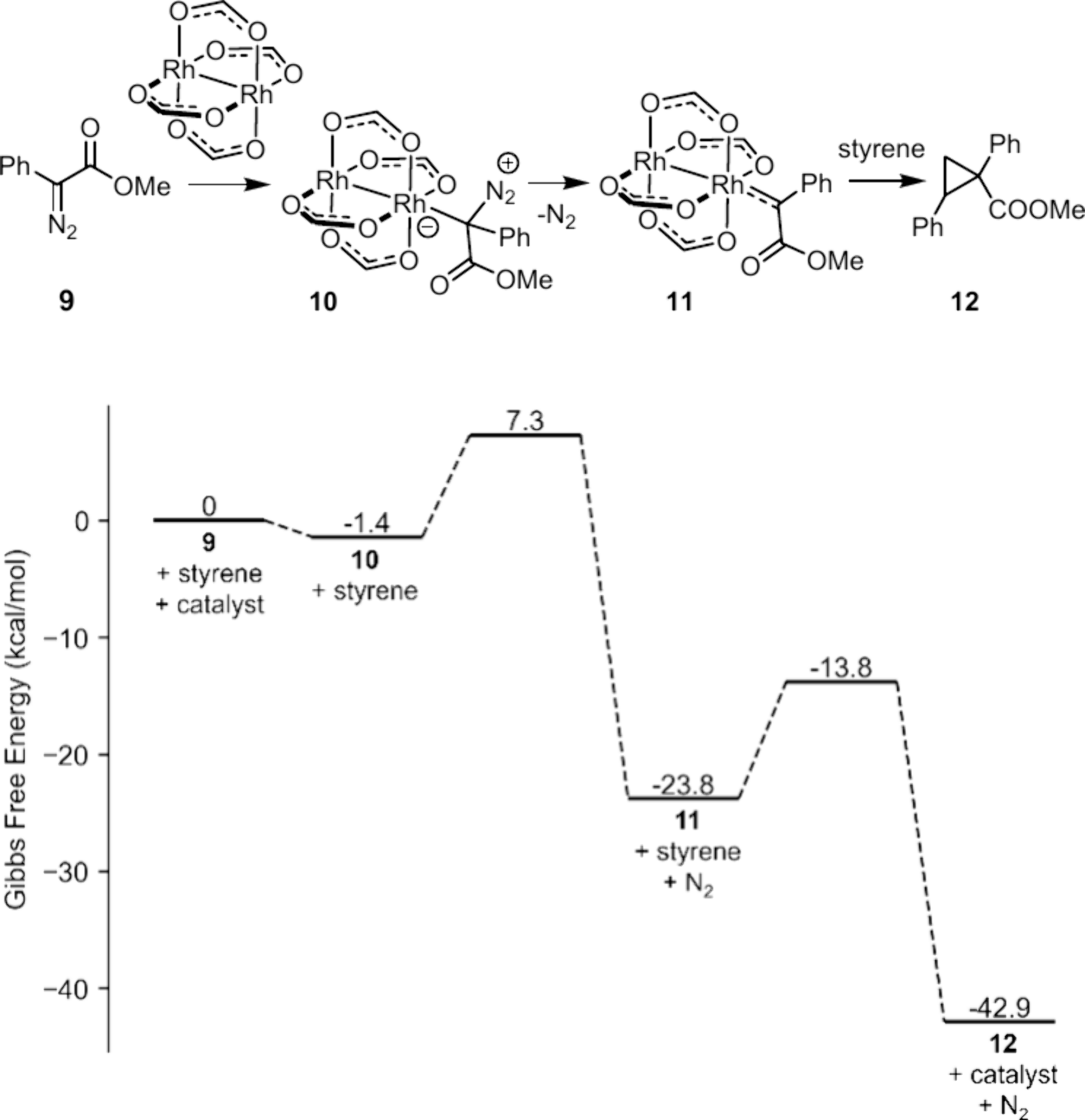
Gibbs Free Energy Profile of the Intermolecular Cyclopropanation of Styrene with Phenyl Diazoacetate **9** Catalyzed by Dirhodium Tetraformate Revisited

No matter at which face the reaction takes place, the computed profile shown in Figure 4 implies that cyclopropane formation is the turnover-limiting step. This conclusion came as a surprise because it contradicts a related computational investigation reported in the literature: specifically, it has been claimed that nitrogen extrusion from ylide **10** derived from diazoester **9** with formation of the carbene intermediate **11** has a notably higher barrier (Δ*E*^⧧^ = 11.3 kcal·mol^–1^) than the subsequent cyclopropanation giving rise to **12** (Δ*E*^⧧^ = 4.5 kcal·mol^–1^) when dirhodium tetraformate was used as the model catalyst (Scheme 4);^50,67^ a recent kinetic study cross-referencing this work seems to reinforce this view.^68^ In addition, strong experimental evidence suggests that the generation of dirhodium carbenes from diazo-*ketones* is turnover-limiting independent of the subsequent chemical reaction (cyclopropanation, C–H insertion, ylide formation).^69,70^

Puzzled by this disaccord, we first chose to recalculate the reaction coordinate for the conversion of **9** → **12** but with our computational settings, which take entropy, solvent (rather than vacuum), thermal corrections, zero-point energy vibrations, and dispersion corrections into account (Scheme 4). Under these conditions, the energetic profile differs from that reported in the literature:^50^ cyclopropanation proves to be turnover limiting in this intermolecular model reaction, just as it is in our intramolecular case shown in Figure 4. Whether this conclusion pertains only to the specific examples under the chosen settings described herein or is more general needs to be seen. In any case, it is of note that the current examples comprise paddlewheel complexes bearing different equatorial ligands (carboxylates, carboxamidates). Much more work needs to be done to understand if and how additives able to ligate the metal centers as transient axial ligands in the intermediates along the reaction coordinate affect the energetic landscape.^71−73^ In the present context, however, such additive effects can be ignored:^74^ as we were aiming to study and compare the innate properties excerted by different sets of equatorial ligands, utmost care was taken to use heteroleptic dirhodium paddlewheel complexes in the absence of any potential external ligands that might compete with the diazo compounds for the axial binding sites.^75^

### In Situ Observation of Reactive Carbene Intermediates in Catalytic Transformations

If carbene formation rather than the subsequent cyclopropanation were turnover limiting, as previously suggested,^50^ any attempt to monitor carbene intermediates in situ would be futile. However, if cyclopropanation has the higher barrier and the carbene complex might even be the catalyst resting state, as suggested by our computational results, the reactive intermediates might build up in a concentration detectable by advanced spectroscopic techniques. In this context, however, we cannot help but emphasize that superelectrophilic dirhodium carbenes had defied direct inspection for decades. It was only in 2013 that Davies, Berry and co-workers reported the ^13^C NMR spectrum and EXAFS data of a donor/acceptor carbene formed by diazo decomposition with a stoichiometric amount of a dirhodium complex.^76^ Shortly thereafter, our group managed to isolate a set of representative dirhodium carbenes and characterize these highly sensitive “superelectrophilic” complexes by spectroscopic as well as crystallographic means.^77−80^

In the present case, the challenge is arguably even higher: to confirm the conclusions drawn from the DFT calculations, the active species must be generated, detected and unambiguously identified in a truly *catalytic* setup, that is with low loadings of the dirhodium paddlewheel catalyst (rather than stoichiometric amounts) in the presence of a reactive olefinic substrate. Even if a small amount of the carbene intermediate is present in such a mixture, as suggested by our computations, it is definitely short-lived: the kinetic experiments showed that the cyclopropanations proceed rapidly at low temperatures. To the best of our knowledge, the literature does not contain any hint that it is possible to detect and monitor a transient dirhodium carbene in situ.

**Figure 6:**
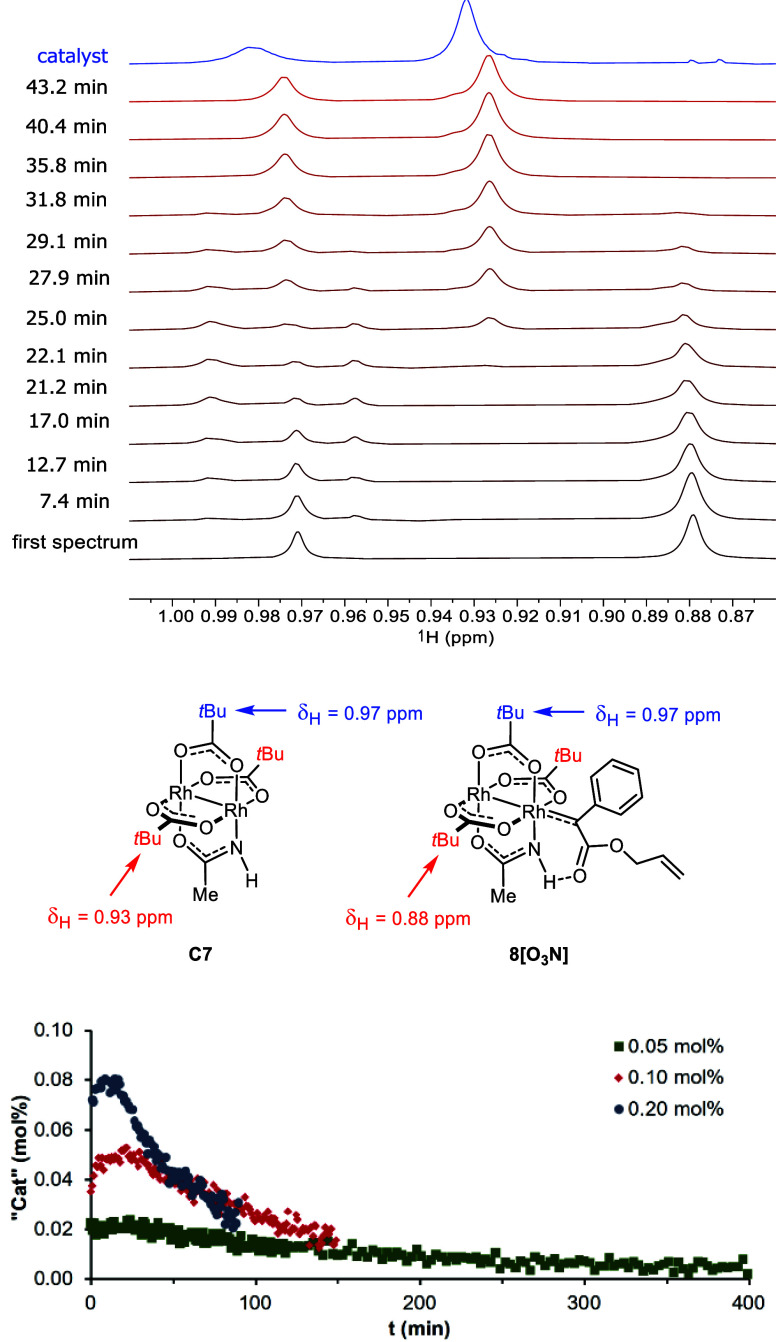
Top: Part of the aliphatic region of the ^1^H NMR spectra (600 MHz, CD_2_Cl_2_, 233 K) showing the two signals of the *tert*-Bu groups *trans* and *cis* (1:2) to the acetamidate ligand of the putative carbene intermediate **8[O**_**3**_**N]** derived from **C7** (0.5 mol%), which disappeared once complete conversion of substrate **6** was reached; at this point, the corresponding signals of the bare catalyst **C7** reappeared in the mixture and gained intensity, which were assigned by comparison to the signals of pure **C7** shown in blue. Bottom: Integral over the signal of the *tert*-Bu groups *cis* to the acetamidate ligand of the putative carbene intermediate **8[O**_**3**_**N]** as a function of time and catalyst loading; the spectra were recorded at 273 K.

Yet we felt encouraged to make such an attempt. It was deemed helpful that catalyst **C7** comprises three *tert*-butyl substituents, which might lead to sufficiently intense peaks even when working with low loadings; they appear in a range of the ^1^H NMR spectrum that is hardly obscured by other signals of the substrate and/or the emerging products. Indeed, when zooming in into the pertinent aliphatic region of the spectra of a mixture comprising diazoester **6** and catalytic amounts of **C7** in CD_2_Cl_2_ at −40 °C, slightly broadened singlets at δ_H_ = 0.97 and 0.88 ppm were detected, which could very well be attributed to two chemically inequivalent *t*Bu-groups (Figure 6).^81,82^ These signals lost intensity with time; once fully disappeared, the corresponding signals of the bare catalyst **C7** at δ_H_ = 0.97 and 0.93 ppm started to grow in and became clearly visible after a short while. Importantly, the signal of the fleeting intermediate observed at the outset of the reaction became gradually more intense in a series of independent experiments in which the catalyst loading was increased from 0.05 to 0.2 mol%. Their tentative attribution to the short-lived dirhodium carbene **8[O**_**3**_**N]** is also based on the fact that the relative intensity of the signals was 1:2, consistent with an intermediate in which one pivalate is *trans* but two privalates are *cis* to the acetamidate ligand.^83,84^

**Figure 7:**
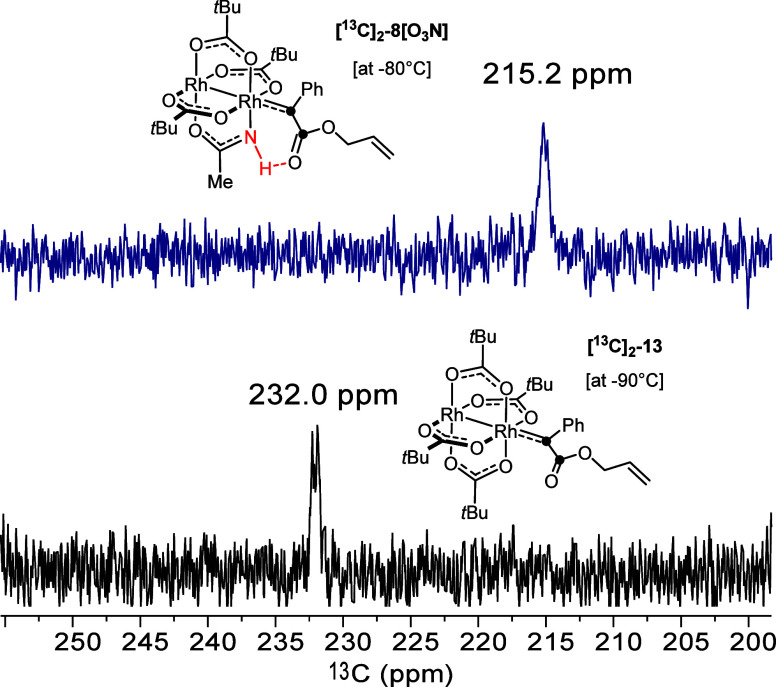
Carbene signals recorded from the crude reaction mixture formed on mixing of [^13^C]_2_-**6** and either catalyst **C7** at −80 °C (top) or [Rh_2_(OPiv)_4_] (5 mol% each) in CD_2_Cl_2_ at −90 °C (bottom); the black dots show the labeled sites.

These data were certainly suggestive but not unequivocal. To confirm that a reactive metal carbene is present in the mixture as a detectable intermediate of the catalytic intramolecular cyclopropanation reaction, it was mandatory to record more characteristic spectral fingerprints. To this end, we resorted to the use the doubly labeled diazoester [^13^C]_2_-**6** as the substrate (readily prepared from the commercially available [^13^C]_2_-phenylacetic acid, see the Supporting Information) and increased the catalyst loading to 5 mol% (Figure 7). When [Rh_2_(OPiv)_4_] was used, the ^13^C NMR spectrum of the reaction mixture recorded in CD_2_Cl_2_ at −90 °C showed a distinct signal at 232.0 ppm, which falls into the range of the few other known (donor/acceptor) dirhodium carbenes residing at Rh[O_4_]-faces (Table 1). The appearance of this resonance as a doublet confirms the vicinity of a second labeled site, which is the ester carbonyl C atom; the observed line broadening is well befitting the proposed carbene intermediate **13** that reacts with the tethered olefin almost on the NMR time scale.^85^ Finally, it is important to note that this carbene signal was no longer detectable once all substrate had been consumed.

**Table 1:**
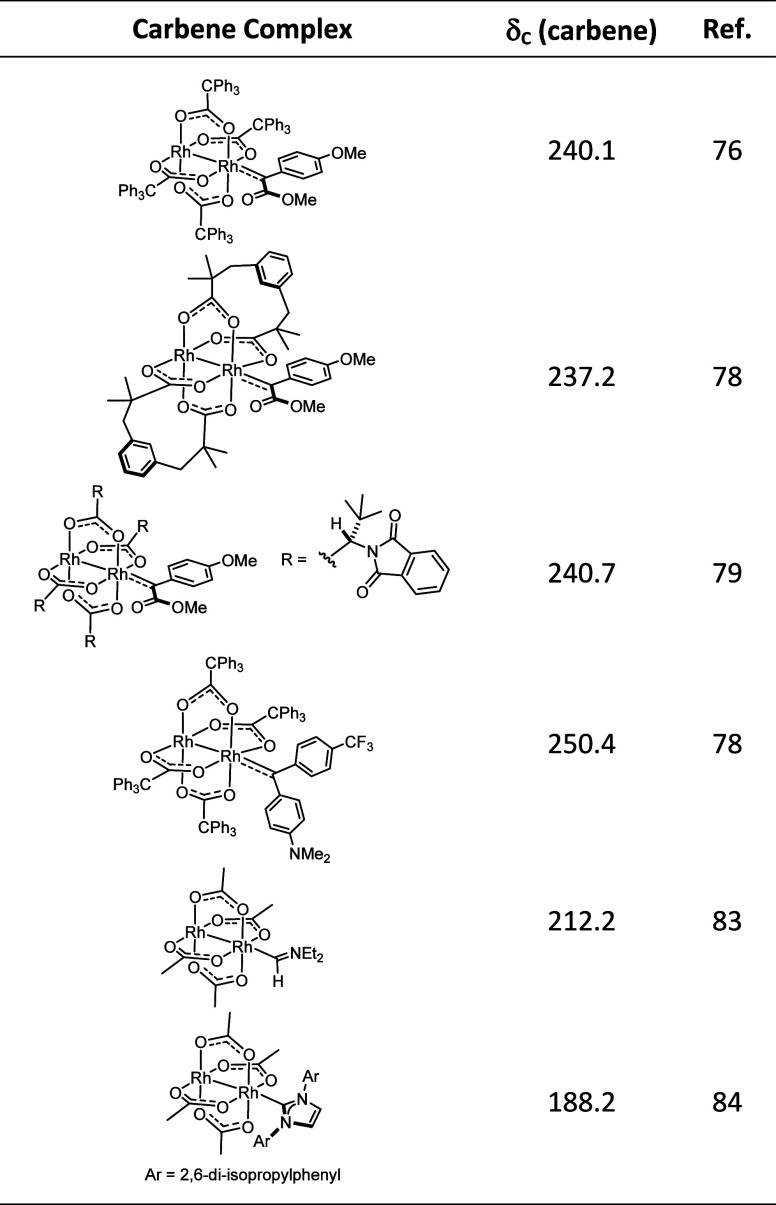
^13^C NMR Shifts of Dirhodium Carbene Complexes

The analogous experiment with the heteroleptic acetamidate-containing catalyst **C7** was more difficult because of the lower solubility of this complex at the very low temperature. This aspect notwithstanding, a broad peak at δ_C_ = 215.2 ppm was recorded, which also disappeared at complete conversion of the substrate. The upfield shift of no less than 16.8 ppm relative to the signal of the [O_4_]-face bound carbene **13** suggests that this resonance belongs to the isotopologue of carbene **8[O**_**3**_**N]** ligated to the Rh[O_3_N]-face, which has been computed to be the notably more stable and somewhat less reactive of the two isomeric intermediates derived from catalyst **C7** (Figure 4).

**Figure 8:**
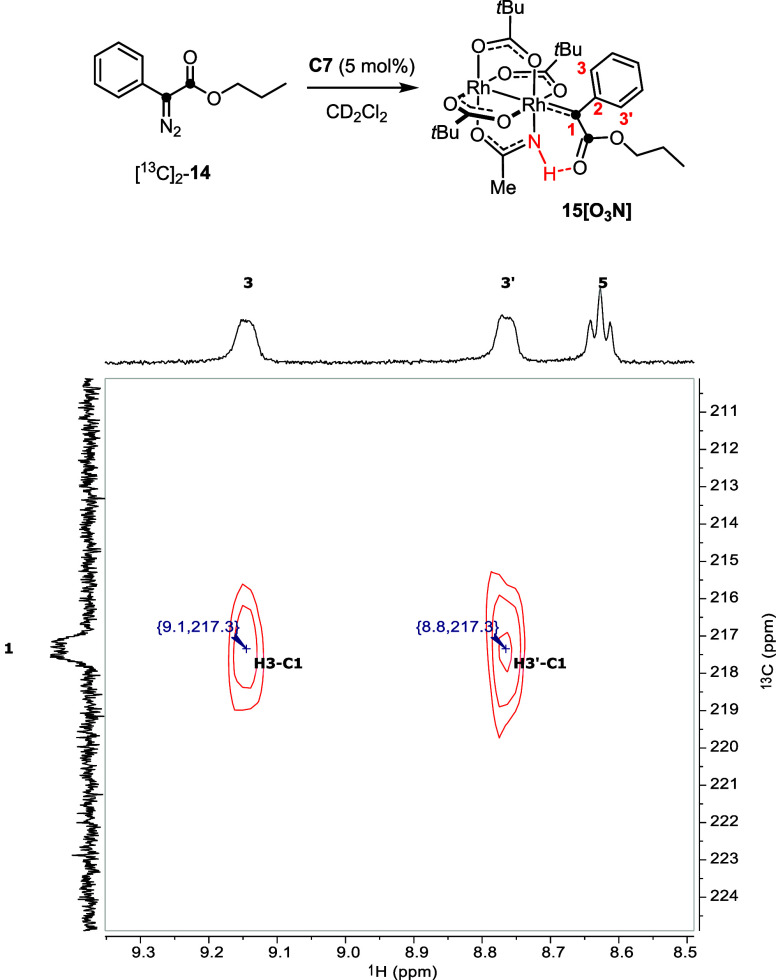
Top: ^1^H–^13^C HMBC (500 MHz, CD_2_Cl_2_, 183 K) showing the cross peak between the signal of the ^13^C-labeled carbene C1 atom of carbene **15[O**_**3**_**N]** and the two *ortho*-H atoms H3 and H3′ on the flanking phenyl ring; arbitrary numbering scheme as shown.

Since no spectroscopic data of dirhodium carbenes derived from carboxamidate-based catalysts are currently available for comparison, further evidence was sought to confirm the assignment. To this end, the analogous propyl (rather than allyl) ester [^13^C]_2_-**14** was prepared and exposed to complex **C7** in the hope that the missing double bond would increase the lifetime of the derived carbene to the extent that further spectroscopic characterization is possible. This turned out to be the case. At −90 °C, fairly clean diazo decomposition took place with formation of a reactive species **15[O**_**3**_**N]** with a distinctive signal at δ_C_ = 217.3 ppm, very similar to the shift observed for the carbene **8[O**_**3**_**N]** derived in situ from the allyl ester [^13^C]_2_-**6** with the same catalyst (Figure 8). Importantly, HMBC cross peaks with two magnetically inequivalent *ortho*-H atoms on the adjacent phenyl ring (δ_H_ = 9.1, 8.8 ppm) were detected, indicating hindered rotation about the Ar–C=M bond. This structural attribute is characteristic of donor/acceptor dirhodium carbenes: our previous crystallographic data had shown that such reactive intermediates adopt a conformation in which the aryl ring is aligned with the carbene center to ensure orbital overlap between the aromatic π-system and the empty p-orbital.^78,79^ At higher temperatures, the signals average out; from these VT NMR data, the barrier to rotation can be estimated to be on the order of 9.8 ± 0.2 kcal·mol^–1^ (for details, see the Supporting Information). The equally labeled ester carbonyl of the reactive intermediate could also be detected as a doublet resonating at δ_C_ = 177.7 ppm.

Although these data leave no serious doubt that the resonances observed at 232.0 ppm and at 215.2/217.3 ppm derive from [O_4_]-bound and [O_3_N]-bound dirhodium carbenes, respectively, this assignment was further validated by computing the ^13^C NMR shifts of such species. This is a nontrivial task, as the shift of the carbene center is strongly influenced by the C–Rh bond, the accurate description of which by DFT is challenging by all means.

Multiple computational setups were tested to compute the ^13^C NMR shifts for **8[O**_**4**_**]** and for **8[O**_**3**_**N]** for the heteroleptic catalyst and **13[O**_**4**_**]** for the homoleptic catalyst. Tetramethylsilane (TMS) was used as reference compound in all NMR calculations (see the Supporting Information). Arguably most significant is the finding that carbene **8[O**_**3**_**N]** was invariably found to resonate at lower frequencies (∼8–10 ppm) than **8[O**_**4**_**]**, independent of the chosen level of theory. In an attempt to obtain accurate shift values, a composite computational method provided the best quantitative agreement with experimental data using TMS as reference. This method combines scalar relativistic single-point NMR calculations at the TPSSh/ZORA-def2-TZVPP + CPCM level using ORCA 5.0.3,^86−90^ with corrections for the spin–orbit coupling (SOC) effect^91^ calculated at PBE0 level using the Amsterdam Density Functional (ADF) 2017 software.^92,93^ With this particular method, the computed chemical shift of **8[O**_**4**_**]** and **8[O**_**3**_**N]** were 235.1 and 227.0 ppm, respectively, which is in remarkably good agreement with the recorded values of 232.0 ppm and 215.2/217.3 ppm. Moreover, the computed ^13^C NMR shift (174.7 ppm) of the ester carbonyl group of **8[O**_**3**_**N]** was also very close to the measured resonance (177.7 ppm), which confirms the validity of the chosen composite method.

## Conclusions

The observation of reactive dirhodium carbenes comprising olefinic sites by NMR in truly catalytic set-ups fully confirms the conclusion reached by our refined DFT calculations that cyclopropanation rather than carbene formation is the turnover-limiting step in the cyclopropanation reaction of alkenes with donor/acceptor diazoesters, at least in the absence of additives, in contrast to what has previously been stated. This is correct for the homoleptic tetracarboxylate dirhodium paddlewheel catalyst [Rh_2_(OPiv)_4_] as well as for the heteroleptic complex **C7** comprising one acetamidate ligand. In the latter case, the carbene bound to the Rh[O_3_N] face was observed by NMR spectroscopy, which is also in excellent agreement with the computational data: this species is the thermodynamically more stable and slightly less reactive of the two isomeric complexes that can derive from this catalyst. This concordance, in turn, corroborates the notion that primary carboxamidates exert a stabilizing function by engaging the ester carbonyl substituent of a classical donor/acceptor carbene in interligand hydrogen bonding.

In settings, in which this enthalpic bonus is maintained along the reaction coordinate, as is the case for the *intermolecular* cyclopropanation shown in Scheme 2, the incorporation of a primary carboxamidate ligand into the catalyst hence entails *mutually reinforcing effects*: the protic equatorial ligand favors carbene formation at the [O_3_N]-face (further assisted by the steric bulk in **C1**) and renders the resulting carbene species more stable as well as more reactive than the isomeric carbene residing at the [O_4_]-face. Downstream of carbene formation, the hydrogen bonding array dictates the trajectory of the incoming olefinic reaction partner and leads to a tight stereodetermining transition state of the actual cyclopropanation event; the protic amidate ligand is hence inherently accountable for the high rates, good yields and remarkable levels of enantioselectivity attainable with such heteroleptic catalysts.^43,44^

The situation is more involved for *intramolecular* settings, especially when smaller equatorial ligands are chosen that allow the incoming diazoester to reach both faces of a heteroleptic catalyst such as **C7** with similar ease (Scheme 3). Although the carbene at the [O_3_N]-face is again considerably more stable by virtue of peripheral hydrogen bonding, this structural feature turns into a handicap as it needs to be distorted or broken when the tethered olefin approaches the reactive center to form the cyclopropane ring. For these *counterbalancing effects*, the catalytic reaction will likely take place to some extent at both faces of **C7**.

From a conceptual viewpoint, the present study shows that many common perceptions as to the actual role of the ligands in dirhodium paddlewheel catalysts are too simplistic and might eventually even miss the point. Rather, one has to beware of generalizations since an understanding for the subtle effects that are operative is only beginning to emerge. This caveat is in perfect agreement with conclusions drawn from complementary investigations into ^103^Rh NMR shifts, that open an entirely new analytical window to study the electronic character of such paddlewheel complexes in previously inconceivable detail.^48,49,94^ At the same time, it is increasingly clear that heteroleptic dirhodium complexes, which so far played a fairly minor role at best, provide unique opportunities for (asymmetric) catalysis, as they allow synergies between steric and electronic factors to be harnessed that their homoleptic counterparts cannot offer. This and related aspects are subject to ongoing studies in our laboratories.

## Supporting Information Available

Experimental section including characterization data, experimental NMR studies, and computational details (PDF)
Effect of the basis set, functional, set and implicit solvation model on the reaction profile on the [O_3_N]-face and the [O_4_]-face and NMR shifts of the carbenic carbon for the intermediates (PDF)
