# Supplementary material for: In Situ Observation of Elusive Dirhodium Carbenes and Studies on the Innate Role of Carboxamidate Ligands in Dirhodium Paddlewheel Complexes: A Combined Experimental and Computational Approach
Source: J Am Chem Soc. 2024 Sep 11;146(38):26466–77. doi: 10.1021/jacs.4c09847 (PMC11440507; doi:10.1021/jacs.4c09847)
Supplement: Supplementary file 1 — ja4c09847_si_001.pdf [file ja4c09847_si_001.pdf]

SUPPORTING INFORMATION - PART 1

PREPARATIVE AND SPECTROSCOPIC DATA

The In Situ Observation of Elusive Dirhodium Carbenes and Studies on The Innate Role of Carboxamidate Ligands in Dirhodium Paddlewheel Complexes.

A Combined Experimental and Computational Approach

Matthias Peeters,[a] Lorenzo Baldinelli,[b] Markus Leutzsch,[a] Fabio Caló,[a] Alexander Auer,[a] Giovanni Bistoni,[b],* and Alois Fürstner[a],*

[a] *Max-Planck-Institut für Kohlenforschung, D-45470 Mülheim/Ruhr, Germany*

[b] *University of Perugia, Department of Chemistry, Biology and Biotechnology, Via Elce di Sotto 8, I-06123 Perugia, Italy*

Emails: fuerstner@kofo.mpg.de; giovanni.bistoni@unipg.it

Table of Contents

Preparation of the Dirhodium Complexes and the Substrates S2
Dirhodium Complexes S2
^{13}C-Labeled Compounds S3
Kinetic experiments S5
Kinetic studies with complexes **C7** - **C10** S5
Kinetic studies with ´bulky´ complexes **C1** and **C5** S6
NMR Studies of the Reactive Intermediates S8
With Diazoester [^{13}C]$_2$-**6** S9
With Diazoester [^{13}C]$_2$-**14** S12
NMR Spectra of New Compounds S17
References S27

Preparation of the Dirhodium Complexes and the Substrates

General. Unless stated otherwise, all reactions were carried out under argon atmosphere in flame-dried Schlenk glassware, ensuring inert conditions. The solvents were purified by distillation over the indicated drying agents and were transferred under argon: THF, Et_2O (Mg/anthracene); pentane, toluene (Na/K); CH_2Cl_2, chlorobenzene (CaH_2). MeCN was dried by an absorption solvent purification system based on molecular sieves. Flash chromatography: Merck Geduran silica gel 60 (40 – 63 µm).

Allyl phenylacetate (98 %) was bought from TCI and used without purification.

$Rh_2(CF_3\text{-acam})(OPiv)_3$(**C8**),[1] $Rh_2(OPiv)_4$,[2] $Rh_2(acam)(TPCP)_3$(**C1**),[3] $Rh_2(OAc)(TPCP)_3$(**C5**),[3] allyl 2-diazo-2-phenylacetate (**6**)[4] were prepared according to literature procedures.

NMR spectra were recorded on a Bruker Avance III HD nanobay 300, Avance III HD 400, Avance III 500 or Avance Neo 600 MHz NMR spectrometer. ^{1}H and ^{13}C NMR chemical shifts are given in ppm relative to Me_4Si (δ = 0 ppm), coupling constants (J) in Hz. ^{1}H and ^{13}C NMR chemical shifts were referenced using the solvent signals as internal reference:[5] (CD_3CN: $\delta(CD_3)$ = 1.32 ppm, residual CHD_2CN: δ_H = 1.94 ppm; $CDCl_3$: δ_C = 77.2 ppm; residual $CHCl_3$: δ_H = 7.26 ppm; CD_2Cl_2: δ_C = 54.0 ppm; residual $CHDCl_2$: δ_H = 5.32 ppm; δ_H = 7.16 ppm). ^{15}N NMR shifts were extracted from the indirect dimension of the ^{1}H-^{15}N HMBC spectra. ^{103}Rh NMR shifts were extracted from the indirect dimension of a 2D H(C)Rh triple resonance spectrum and were generally acquired with a 5 mm TBI probe (^{1}H, ^{31}P,^{109}Ag, ^{13}C) with z-gradient coil on an Avance III 500 MHz NMR spectrometer at 298K.[6] The probe could be tuned beyond the specifications to ^{103}Rh on the broadband X-channel. ^{103}Rh, ^{15}N and ^{19}F chemical shifts were referenced indirectly to the ^{1}H chemical shift of the solvent according to IUPAC recommendations using the xiref macro in Bruker Topspin[7]. ^{15}N chemical shifts are reported relative to CH_3NO_2 (δ = 0 ppm; Ξ = 10.136767%), ^{19}F chemical shifts are reported relative to CCl_3F (δ = 0 ppm; Ξ = 94.094 011%) and ^{103}Rh chemical shifts were referenced with Ξ = 3.16%.

IR: Alpha Platinum ATR (Bruker), wavenumbers ($\tilde{\nu}$) in cm^{-1}; mdium and weak resonances were omitted.

MS (EI): Finnigan MAT 8200 (70 eV), ESI-MS: ESQ 3000 (Bruker) or Thermo Scientific LTQ-FT or Thermo Scientific Exactive Spectrometer. HRMS: Bruker APEX III FT-MS (7 T magnet), MAT 95 (Finnigan), Thermo Scientific LTQ-FT or Thermo Scientific Exactive Spectrometer.

Dirhodium Complexes

Dirhodium(II) (acetamidate)tris(trifluoroacetate) [$Rh_2(acam)(OTfa)_3$] (S1)

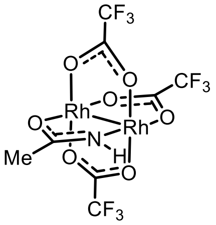

[$Rh_2(acam)_4\cdot 2H_2O$] (400 mg, 0.84 mmol) was filled into a flame-dried two neck round bottom flask with an attached reflux condenser. Trifluoroacetic acid (16 mL, 210 mmol) was added and the resulting suspension was stirred at 60 °C (bath temperature) for 22 min. The mixture was then quickly cooled to 0°C in an ice bath. All volatile materials were distilled off under reduced pressure. The remaining crude material was purified by flash chromatography (silica, toluene/MeCN 9:1) to give the title compound as a green solid (424 mg, 79%). ^{1}H NMR (600 MHz, CD_3CN): δ 5.40 (s, 1H), 1.90 (s, 3H) ppm. ^{13}C NMR (151 MHz, CD_3CN): δ 191.33, 175.71 (q, J = 39.1 Hz), 173.83 (q, J = 38.9 Hz), 112.80 (q, J = 285.1 Hz), 111.61 (q, J = 284.7 Hz), 24.09 ppm. ^{19}F NMR (565 MHz, CD_3CN): δ −75.77, −75.84 ppm. HRMS (ESI $^{-}$): m/z calcd. for $C_8H_3F_9NO_7Rh_2$ $[M-H]^-$: 601.7881; found: 601.7885.

Dirhodium(II) (acetamidate)tris(pivalate) [Rh_2(acam)(OPiv)$_3$] (C7)

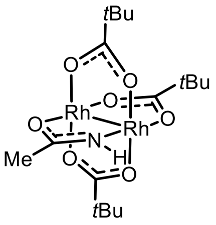

Pivalic acid (29.4 mg, 0.29 mmol) was filled into a flame-dried pressure Schlenk flask and dissolved in MeCN (0.8 mL). *N*-Ethyldiisopropylamine (48 µL, 0.27 mmol) was added and the mixture left stirring for 10 min. A solution of complex **S1** (55 mg, 0.09 mmol) in MeCN (2.4 mL) was added dropwise to this mixture and stirring was continued at 60°C (bath temperature) for 18 h. The mixture was cooled to room temperature and concentrated. The residue was purified by flash chromatography (silica, toluene/acetonitrile 5:1) to give the title compound as a green solid (19.8 mg, 38%). The spectral data were consistent with the literature.[6]

Dirhodium(II) (acetate)tris(pivalate) [Rh_2(OAc)(OPiv)$_3$] (C9)

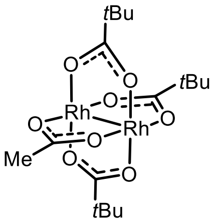

A flame-dried Schlenk flask was charged with *n*-tetrabutylammonium acetate (11 mg, 0.04 mmol) and MeCN (0.5 mL). A solution of Rh_2(OTfa)(OPiv)$_3$ (16 mg, 0.03 mmol) in MeCN (1 mL) was added dropwise to the mixture and stirring was continued for 3 h at room temperature. The mixture was concentrated and the residue purified by flash chromatography (silica, toluene/acetonitrile 8:1) to afford the title compound as a green solid (10 mg, 68%). ^{1}H NMR (600 MHz, CD_3CN) δ 1.76 (s, 3H), 0.92 (s, 18H), 0.90 (s, 9H). ^{13}C NMR (151 MHz, CD_3CN) δ 199.8, 199.7, 41.0, 40.8, 28.0, 28.0, 23.6. ^{103}Rh NMR (H(C)Rh, 15.9 MHz, CD3CN) δ 7304. HRMS (ESI$^+$): m/z calcd. for $C_{17}H_{30}NaO_8Rh_2$ [M+Na]$^+$: 590.99430; found: 590.99409.

Dirhodium(II) (trifluoroacetate)tris(pivalate) [Rh_2(OTfa)(OPiv)$_3$] (C10)

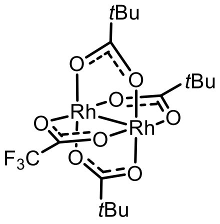

A flame dried pressure Schlenk flask was charged with [Rh_2(OTfa)$_4$] (69 mg, 0.10 mmol). Pivalic acid (32 mg, 0.31 mmol), acetonitrile (4 mL) and *N*-ethyldiisopropylamine (55 µL, 0.31 mmol) were added and the resulting mixture was stirred at 60°C (bath temperature) overnight. The mixture was cooled to room temperature and concentrated. The residue was purified by flash chromatography (silica, toluene/acetonitrile 20:1 to 10:1) to give the title compound as a green solid (26 mg, 40%). ^{1}H NMR (600 MHz, CD_3CN) δ 0.93 (s, 18H), 0.92 (s, 9H). ^{13}C NMR (151 MHz, CD_3CN) δ 201.8, 201.2, 173.9 (q, J = 38.5 Hz), 111.9 (q, J = 285.1 Hz), 41.3, 41.2, 27.9 (2 C). ^{19}F NMR (565 MHz, CD_3CN) δ -75.97. ^{103}Rh NMR (H(C)Rh, 15.9 MHz, CD_3CN) δ 7386. HRMS (ESI$^+$): m/z calcd. for $C_{17}H_{27}F_3NaO_8Rh_2$ [M+Na]$^+$: 644.96603; found: 644.96643.

^{13}C-Labeled Compounds

Esterification: General Procedure

To a solution of commercial doubly-^{13}C-labeled phenylacetic acid (1 equiv.) in CH_2Cl_2 (0.1 M) were added the corresponding alcohol alcohol (1.5 equiv.) and DMAP (0.1 equiv.). The mixture was cooled to 0° C and stirred for 15 min prior to the portionwise addition of N-ethyl-N'-(dimethylaminopropyl)-carbodiimide hydrochloride (1.5 equiv.). After full consumption of the starting material, the reaction was diluted with water (30 mL) and extracted with CH_2Cl_2 (3 x 20 mL). The combined organic phases were washed with brine (30 mL), dried over $MgSO_4$ and concentrated under reduced pressure. The

residue was purified by flash chromatography (silica, pentane/*tert*-butyl methyl ether 19:1) to afford the corresponding doubly-labeled ester.

Allyl 2-phenyl-1,2-[$^{13}C]_2$-acetate ([$^{13}C]_2$-S2)

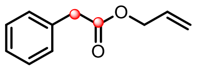

Colorless oil (470 mg, 91 %). ^{1}H NMR (400 MHz, $CDCl_3$) δ 7.36 – 7.27 (m, 5H), 5.91 (ddt, *J* = 17.2, 10.4, 5.7 Hz, 1H), 5.27 (dq, *J* = 17.2, 1.5 Hz, 1H), 5.22 (dq, *J* = 10.4, 1.3 Hz, 1H), 4.64 – 4.56 (m, 2H), 3.66 (dd, *J* = 129.6, 7.9 Hz, 2H).^{13}C NMR (101 MHz, $CDCl_3$) 171.4 (d, *J* = 57.5 Hz), 134.1 (dd, *J* = 43.9, 3.0 Hz), 132.2 (d, *J* = 2.2 Hz), 129.71 – 129.21 (m), 128.7 (d, *J* = 3.7 Hz), 127.3, 118.4, 65.6 (dd, *J* = 2.5, 1.2 Hz), 41.5 (d, *J* = 57.7 Hz). IR (ATR): ṽ = 1690, 1496, 1454, 1272, 1222, 1123, 986, 928, 758, 696 cm^{-1}. HRMS (EI): [M]$^+$: 178.089890; found: 178.089790.

Propyl 2-phenyl-1,2-[$^{13}C]_2$-acetate ([$^{13}C]_2$-S3)

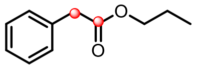

Colorless oil (287 mg, 89 %). ^{1}H NMR (400 MHz, $CDCl_3$) δ 7.37 – 7.21 (m, 5H), 4.06 (td, *J* = 6.7, 3.0 Hz, 2H), 3.62 (dd, *J* = 129.5, 7.9 Hz, 2H), 1.71 – 1.58 (m, 2H), 0.91 (t, *J* = 7.4 Hz, 3H). ^{13}C NMR (101 MHz, $CDCl_3$) δ 171.8 (d, *J* = 57.6 Hz), 134.3 (dd, *J* = 43.8, 2.9 Hz), 129.42 – 129.31 (m), 128.7 (d, *J* = 3.8 Hz), 127.2, 66.6 (dd, *J* = 2.7, 1.1 Hz), 41.6 (d, *J* = 57.6 Hz), 22.1 (d, *J* = 2.2 Hz), 10.5. IR (ATR): ṽ = 2968, 1689, 1496, 1455, 1227, 1131, 969, 703 cm^{-1}. HRMS (EI): [M]$^+$: 180.105540; found: 180.105390.

Diazotation: General Procedure

The ^{13}C-labeled diazo esters were prepared according to the literature describing the non-labeled compounds. A solution of the ^{13}C labeled ester and 4-acetamidobenzenesulfonyl azide (1.3 equiv.) in acetonitrile was cooled with an ice bath before DBU (1.3 equiv.) was added. The mixture was warmed to room temperature and stirred overnight before it was diluted with water (30 mL). The mixture was extracted with CH_2Cl_2 (2 x 25 mL) and the combined organic layers were washed with water (50 mL), dried over $MgSO_4$ and concentrated under reduced pressure in the presence of Celite. The crude product was purified by flash chromatography (silica, pentane/EtOAc 50:1 to 30:1), to give the corresponding ^{13}C labeled diazo.

Allyl 2-diazo-2-phenyl-1,2-[$^{13}C]_2$-acetate ([$^{13}C]_2$-6)

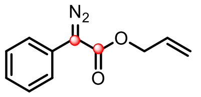

Red oil (250 mg, 68 %). ^{1}H NMR (400 MHz, $CDCl_3$) δ 7.54 – 7.45 (m, 2H), 7.44 – 7.34 (m, 2H), 7.23 – 7.14 (m, 1H), 5.99 (ddt, *J* = 17.2, 10.5, 5.6 Hz, 1H), 5.37 (dq, *J* = 17.2, 1.5 Hz, 1H), 5.28 (dq, *J* = 10.4, 1.3 Hz, 1H), 4.82 – 4.74 (m, 2H).^{13}C NMR (101 MHz, $CDCl_3$) δ 165.0 (d, *J* = 95.9 Hz), 132.2 (d, *J* = 2.1 Hz), 129.1 (d, *J* = 4.6 Hz), 126.0 (d, *J* = 1.2 Hz), 125.6 (dd, *J* = 69.2, 3.7 Hz), 124.2 (dd, *J* = 2.9,1.4 Hz), 118.5, 65.8 – 65.3 (m), 63.4 (d, *J* = 95.9 Hz). IR (ATR): ṽ = 2079, 1659, 1497, 1308, 1210, 1130, 1041, 1014, 993, 932, 750, 690, 667 cm^{-1}. HRMS (ESI$^+$): [M+Na]$^+$: 227.07016; found: 227.07013.

Propyl 2-diazo-2-phenyl-1,2-[$^{13}C]_2$-acetate ([$^{13}C]_2$-14)

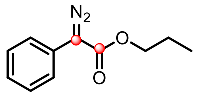

Red oil (96 mg, 42 %). ^{1}H NMR (400 MHz, CDCl3) δ 7.53 – 7.44 (m, 2H), 7.43 – 7.33 (m, 2H), 7.23 – 7.14 (m, 1H), 4.24 (td, *J* = 6.6, 3.1 Hz, 2H), 1.80 – 1.67 (m, 2H), 0.99 (t, *J* = 7.4 Hz, 3H). ^{13}C NMR (101 MHz, $CDCl_3$) δ 165.4 (d, *J* = 95.3 Hz), 125.9 (d, *J* = 1.2 Hz), 125.8 (dd, *J* = 65.3, 3.8 Hz), 124.1 (dd, *J* = 2.9, 1.4 Hz), 66.9 – 66.4 (m), 63.4 (d, *J* = 95.3 Hz), 22.3 (d, *J* = 2.2 Hz), 10.5. IR (ATR): ṽ = 2969, 2080, 1661, 1497, 1310, 1213, 1136, 1062, 1007, 752, 691 cm^{-1}. HRMS (ESI$^+$): [M+Na]$^+$: 229.08581; found: 229.08568.

Kinetic experiments

Kinetic studies with complexes **C7** - **C10**

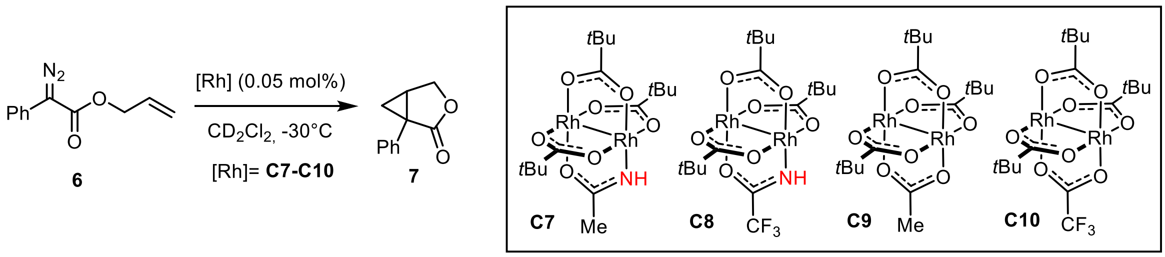

Stock solution of the catalyst: c = 1 mg/mL in acetonitrile

Stock solution of the diazo ester: c = 100 mg/mL in CD_2Cl_2

A flame-dried NMR tube under argon was charged with an aliquot of the stock solution of the catalyst (0.05 mol%). The solvent was evaporated in vacuo and the NMR tube gently heated to dry the catalyst. Then the dried catalyst was dissolved in CD_2CL_2 (0.1 mL). The NMR tube was cooled to −78°C in a dry ice/ethanol mixture. The solution was layered with CD_2CL_2 (0.3 mL) before an aliquot of the stock solution of the diazo ester (50 µL) was carefully added. The solution was mixed by turning the tube upside down and vortexing; the tube was then immediately transferred to the NMR probe head precooled to –30 °C. After fast shimming, single scan ^{1}H NMR spectra were acquired every 60 s until full conversion of the starting material was observed.

The recorded NMR data was imported into MNOVA 15.0.1 with the reaction monitoring plugin for processing (baseline correction, phasing, integration). The data from the first spectrum was used as concentration reference (100 mol%) for the subsequent spectra. An overview of the obtained reaction profiles is shown in Figure S1(A).

In order to extract the relative reaction rates of the different catalysts, a variable time normalization approach inspired by the work Burés[8] was used. Instead of normalizing it to a concentration of the catalyst, we multiplied the time scale with a factors k_x/k_{ref} in order to obtain a rate relative to a reference reaction. In the current dataset all of the rates are given relative to the reaction with catalyst **C7**, which was the slowest reaction. All the time-normalized data with the relative rates used is shown in Figure S1(B).

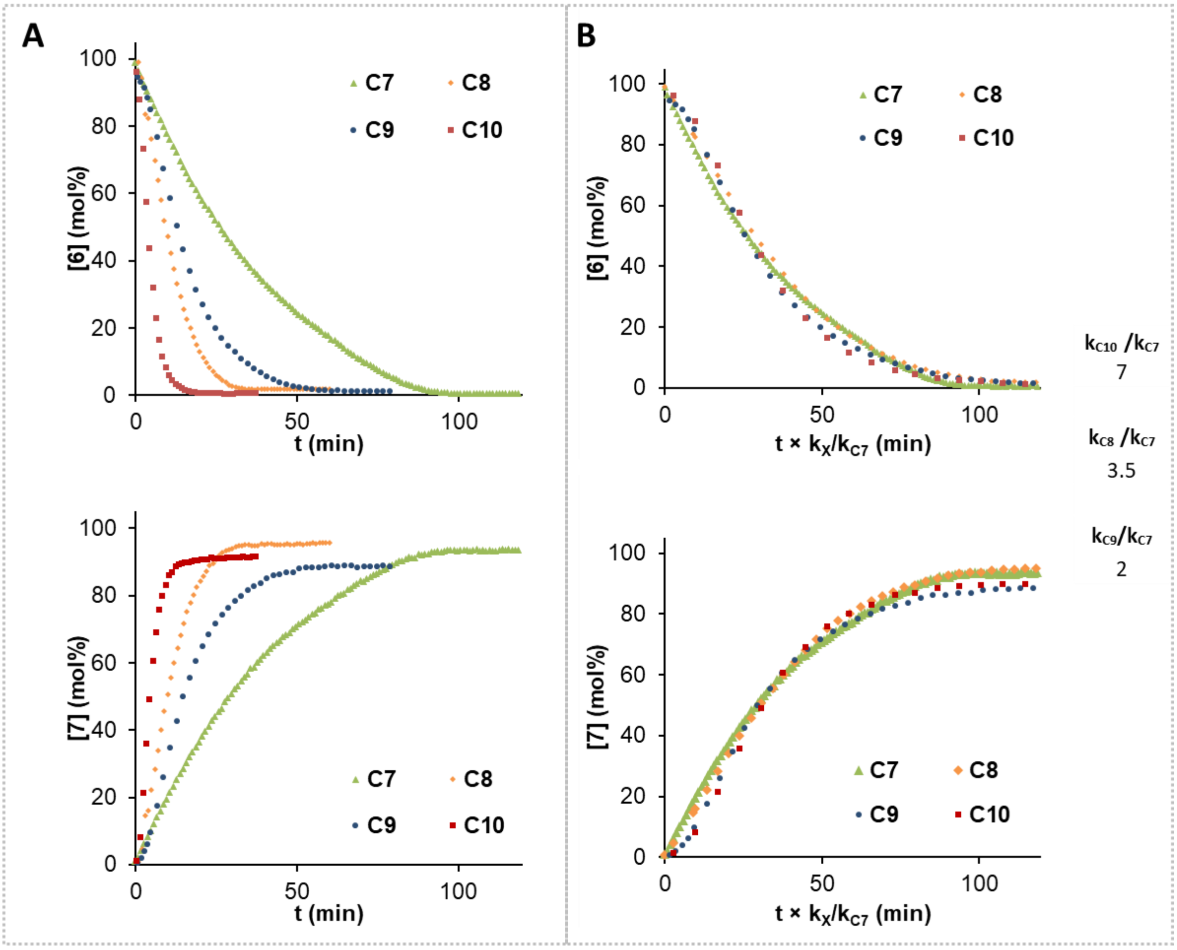

Figure S1. A: Reaction profiles of the consumption of **6** and formation of **7** using catalysts **C7**, **C8**, **C9**, **C10**. B: Reaction profiles with time-normalized scales; the relative rates on the right are values used in these graphs.

Kinetic studies with ´bulky´ complexes C1 and C5

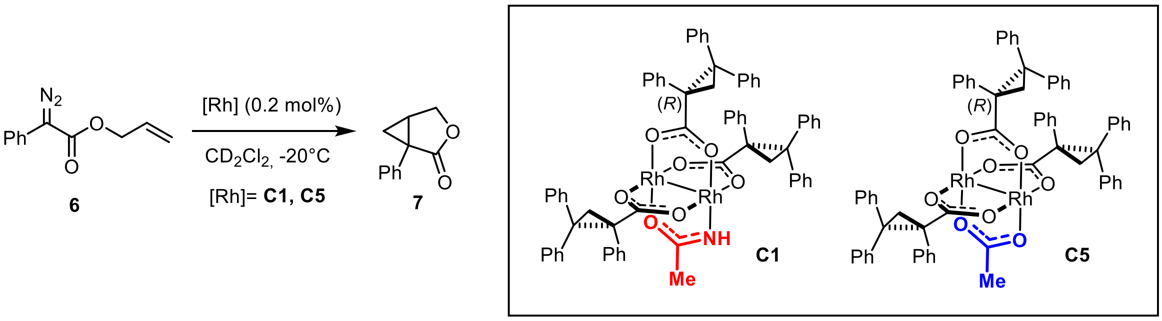

Stock solution of the catalyst: c = 1 mg/mL in acetonitrile

Stock solution of the diazo ester: c = 100 mg/mL in CD_2Cl_2

A flame-dried NMR tube under Argon was charged with an aliquot of the stock solution of the catalyst (0.2 mol%). The solvent was evaporated in vacuo and the NMR tube heated to dry the catalyst. Then the dried catalyst was dissolved in CD_2CL_2 (0.1 mL). The NMR tube was cooled to −78°C in a dry ice/ethanol mixture. The solution was layered with CD_2CL_2 (0.3 mL) before an aliquot of the stock

solution of the diazo ester (50 µL) was carefully added. The solution was mixed by turning the tube upside down and vortexing; the tube was then immediately transferred to the NMR probe head precooled to −20 °C. After fast shimming, single scan ^{1}H NMR spectra were acquired every 60 s until full conversion of the starting material was observed.

The NMR data was imported into MNOVA 15.0.1 with the reaction monitoring plugin for processing (baseline correction, phasing, integration). The data from the first spectrum was used as concentration reference (100 mol%) for the following spectra. An overview of the obtained reaction profiles and the time-normalized data is shown in Figure S2.

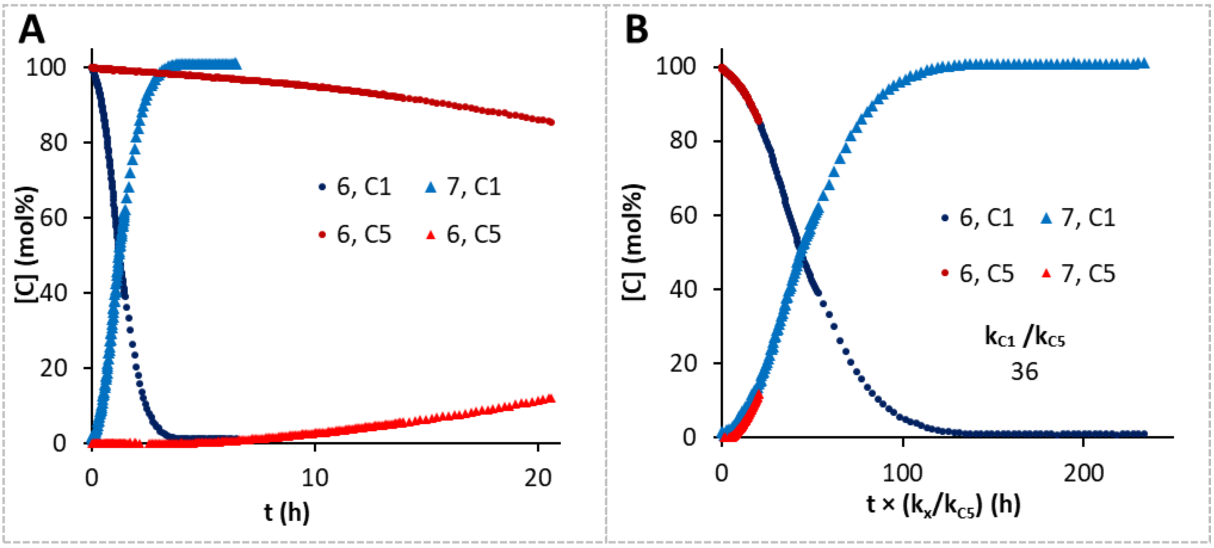

Figure S2. A: Reaction profiles of the consumption of **6** and formation of **7** using catalysts **C1** and **C5**. B: Reaction profiles with time-normalized scales; the relative rate is shown as insert in the graph.

NMR Studies of the Reactive Intermediates

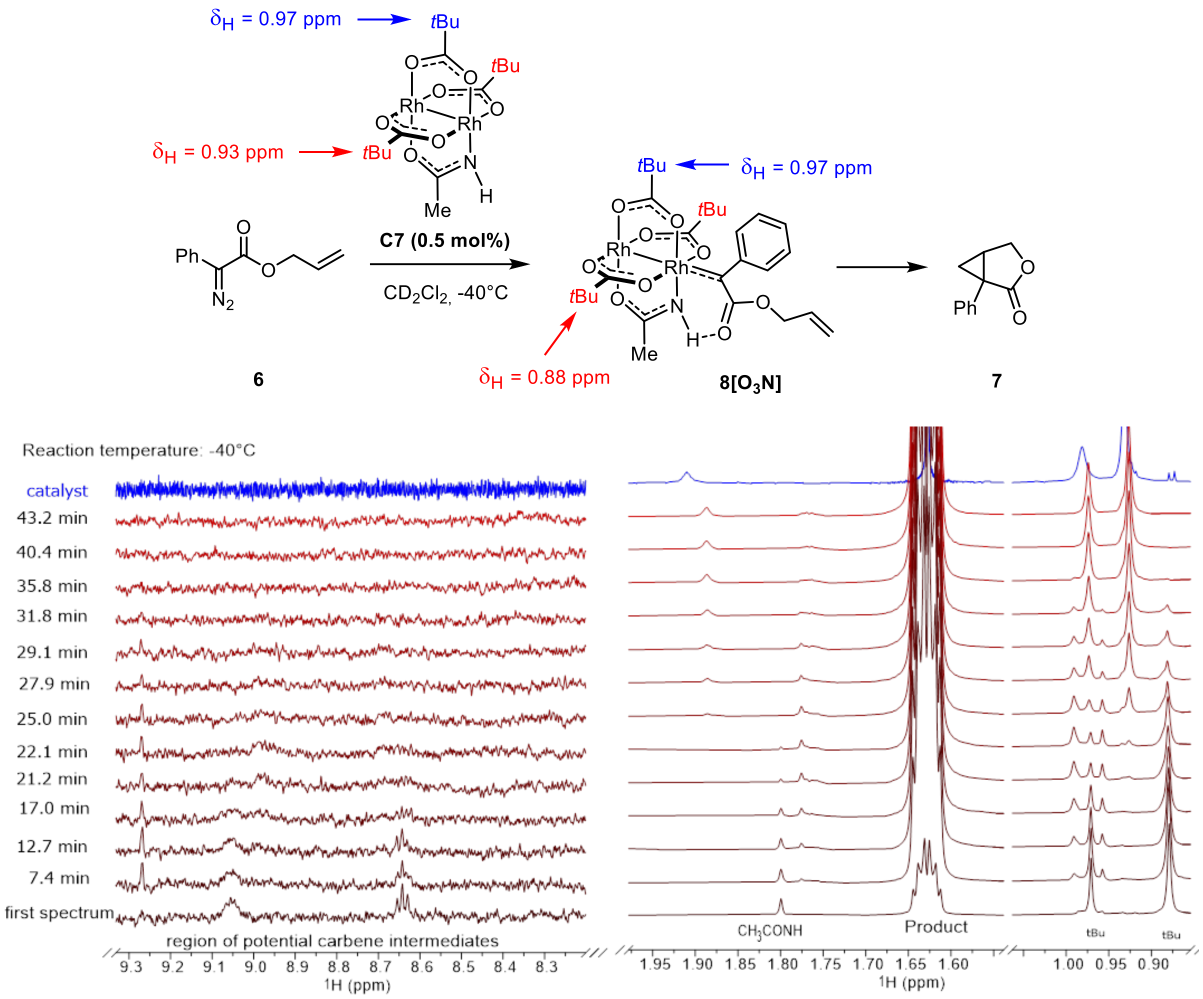

Figure S3. Aromatic and aliphatic regions of the ^{1}H NMR spectra (600 MHz, CD_2Cl_2, 233 K) showing the two signals of the *tert*-Bu groups *trans* and *cis* (1:2) to the acetamidate ligand of the putative carbene intermediate **8[O₃N]** derived from **C7** (0.5 mol%), which disappeared once complete conversion of substrate **6** was reached. This reactive intermediate also shows characteristic signals at 8.65 and 9.05 ppm, which are attributed to the *ortho*-H atoms of the adjacent phenyl ring; the assignment is based on the ^{1}H-^{13}C HMBC cross peaks observed with the ^{13}C-labelled substrate [^{13}C]$_2$-**14** (Figure 8 of the main text, Figure S13) and analogous signals visible in Figures S4, S6, S7, S8 and S9. The shift difference between the methyl group of the acetamidate ligands in **C7** and **8[O₃N]** is ca. –0.1 ppm.

Once the signals of **8[O₃N]** have fully disappeared, the corresponding signals of the bare catalyst **C7** reappeared in the mixture and gained intensity, which were assigned by comparison to the signals of pure **C7** shown in blue.

The additional weak signals visible in the spectra recorded between 12.7 - 27.9 min are unlikely to be those of **8[O₄]** or any other second carbene intermediate because this transient species lacks the caracteristic downfield signals of the two *ortho*-H atoms of the adjacent phenyl ring; its identity is currently unknown.

With Diazoester $[^{13}C]_2$-**6**

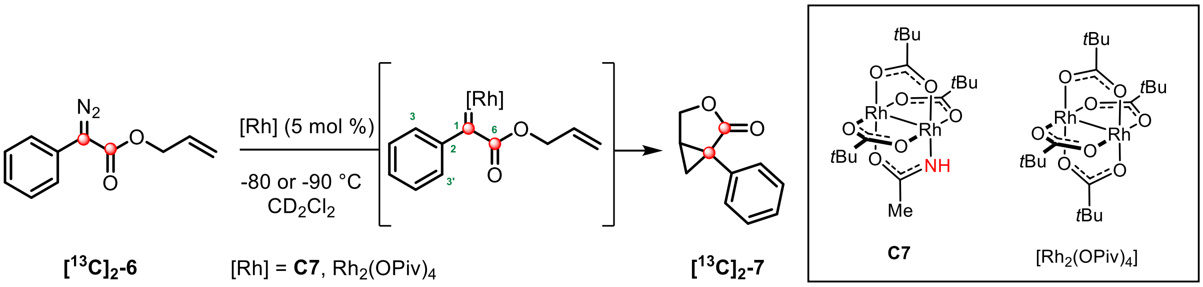

Stock solution of the catalyst: c = 1 mg/mL in acetonitrile

Stock solution of the labeled diazo ester: c = 100mg/mL in CD_2Cl_2

A flame-dried NMR tube under Argon was charged with an aliquot of the stock solution of the catalyst (5 mol %). The solvent was evaporated in vacuo and the NMR tube heated to dry the catalyst. The dried catalyst was dissolved in CD_2CL_2 (0.1 mL). The NMR tube was cooled to −78°C in a dry ice/ethanol mixture. The cold solution was layered with CD_2CL_2 (0.35 mL) before n aliquot of the stock solution of the labeled diazoester (50 µL) was carefully added. The solution was mixed immediately before the tube was inserted into the NMR probe precooled to –80°C in case of Rh_2(acam)(OPiv)$_3$ **(C7)** or –90°C in case of $Rh_2(OPiv)_4$. After quick shimming, the NMR spectra were immediately acquired.

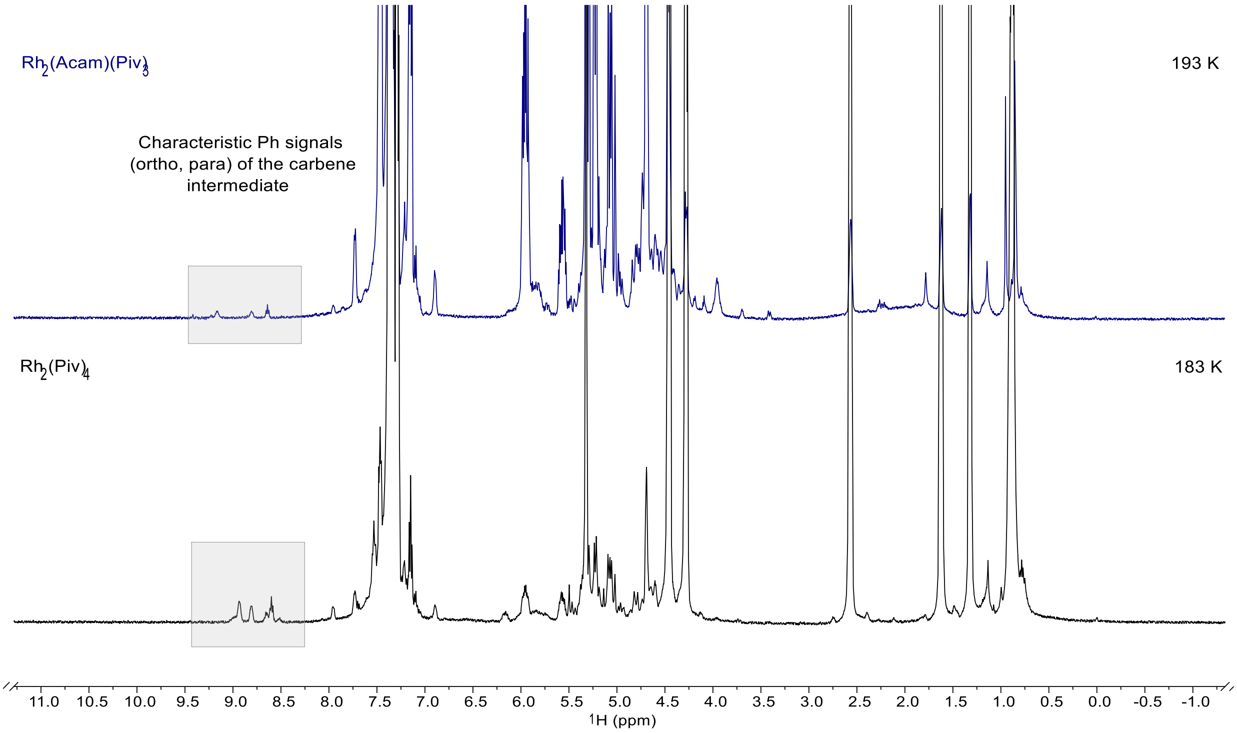

Figure S4. ^{1}H NMR spectra of the different reaction mixtures using catalyst **C7** and **C9** at 193 K and 183 K respectively.

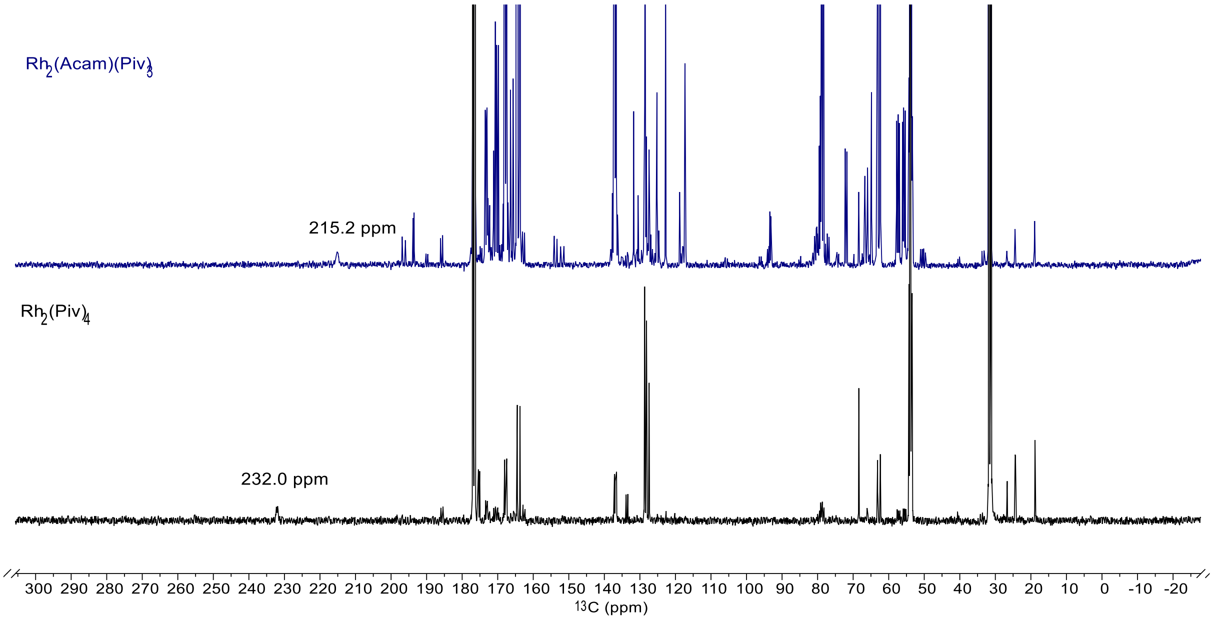

Figure S5. ^{13}C NMR spectra of the different reaction mixtures using catalyst **C7** and **C9** at 193 K and 183 K, respectively.

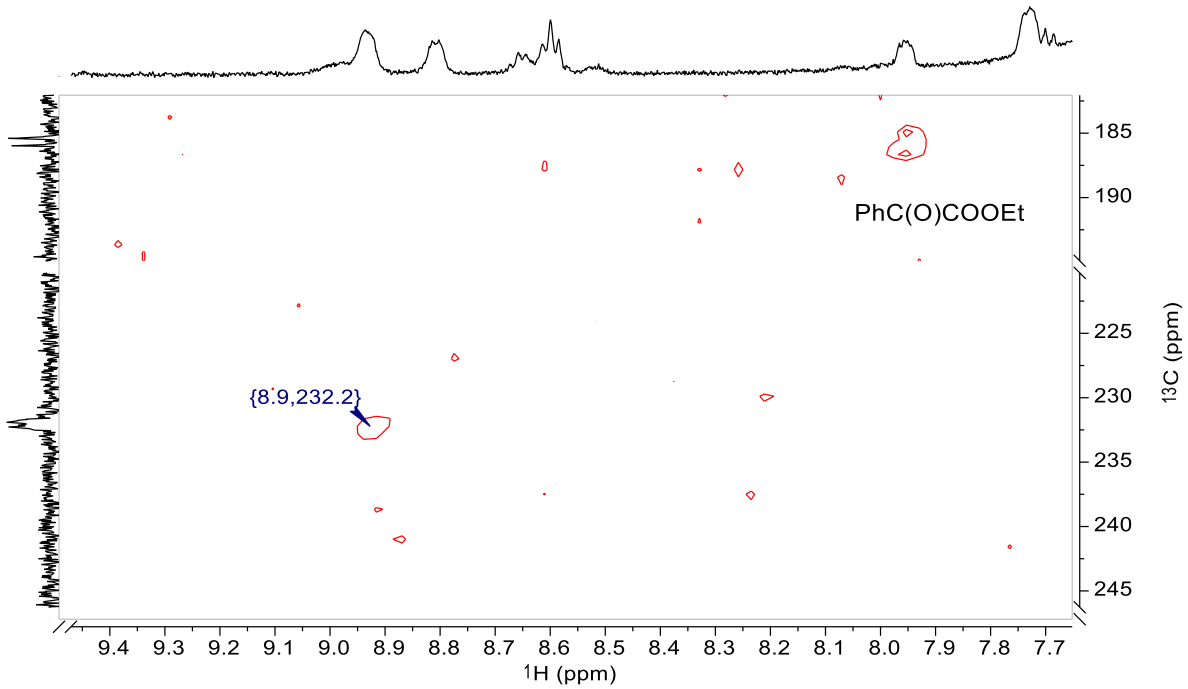

Figure S6. ^{1}H-^{13}C HMBC at 183K showing the cross peak to the carbene signal from the reaction using catalyst **C9**

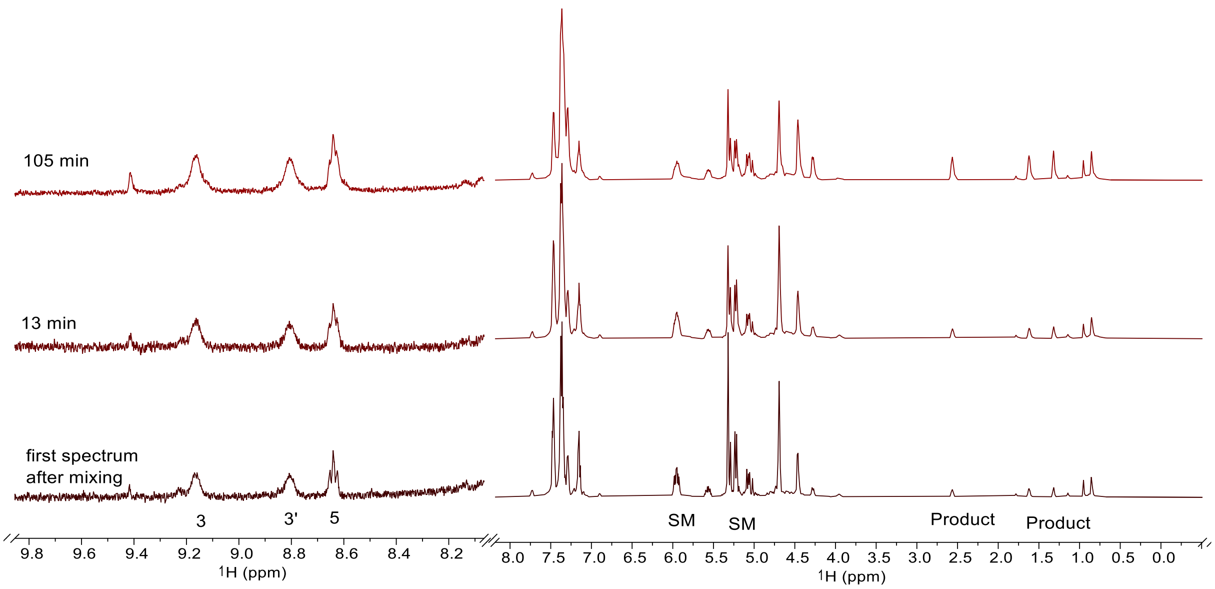

Figure S7. ^{1}H NMR spectra of the reaction with catalyst **C7** taken at different time points after mixing and insertion of the tube into the NMR instrument.

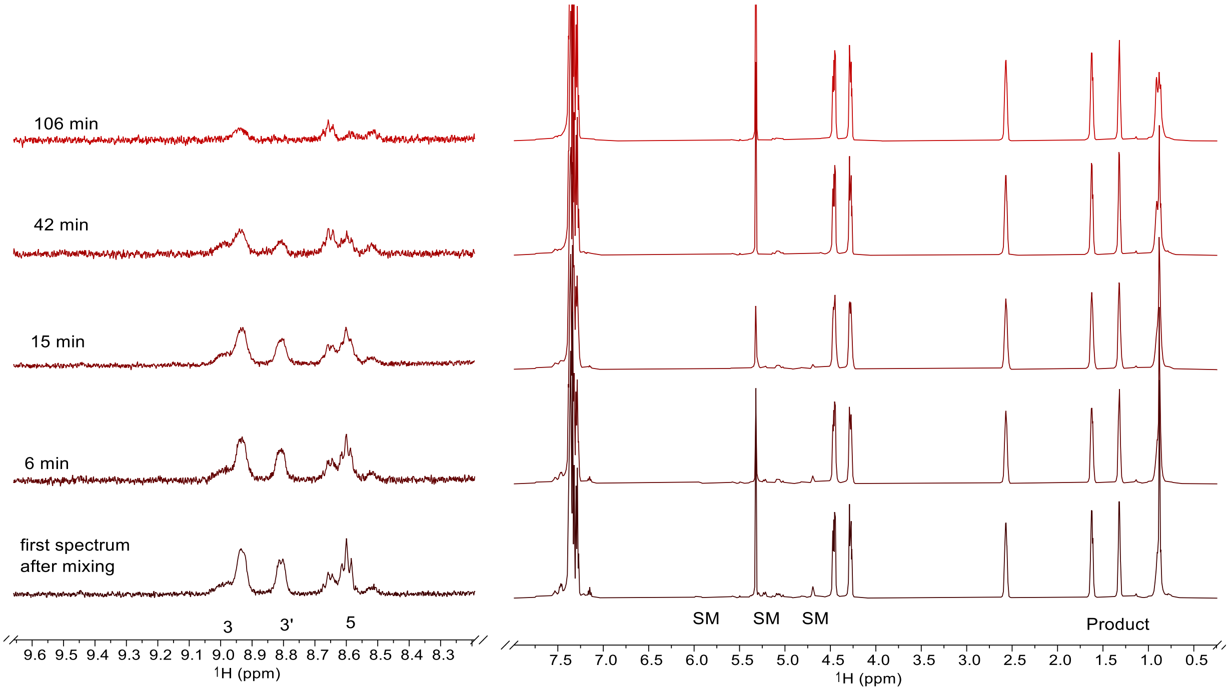

Figure S8. ^{1}H NMR spectra of the reaction with catalyst **C9** taken at different time points after mixing and insertion of the tube into the NMR instrument.

With Diazoester $[^{13}C]_2$-**14**

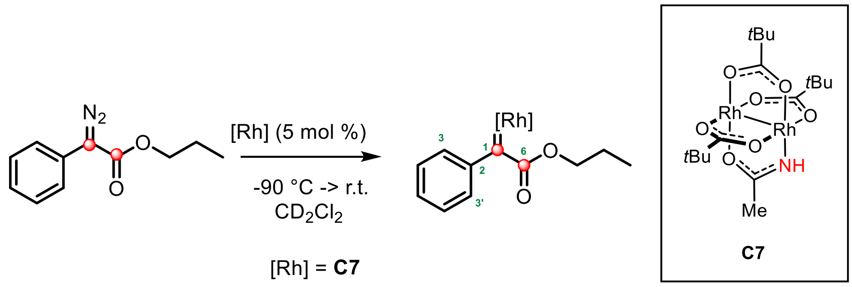

Stock solution of the catalyst: c = 1 mg/mL in acetonitrile)

Stock solution of the labeled diazoester: c = 100mg/mL in CD_2Cl_2

A flame-dried NMR tube under Argon was charged with an aliquot of the stock solution of the catalyst (5 mol %). The solvent was evaporated in vacuo and the NMR tube heated to dry the catalyst. The dried catalyst was dissolved in CD_2CL_2 (0.1 mL). The NMR tube was cooled to −78°C in a dry ice/ethanol mixture. The cold solution was layered with CD_2CL_2 (0.35 mL) before an aliquot of the stock solution of the labeled diazoester (50 µL) was carefully added. The solution was mixed immediately before the tube was inserted into the NMR probe precooled to –80°C in case of $Rh_2(acam)(OPiv)_3$ (**C7**) and to –90°C in case of $Rh_2(OPiv)_4$. After quick shimming, the NMR spectra were immediately acquired.

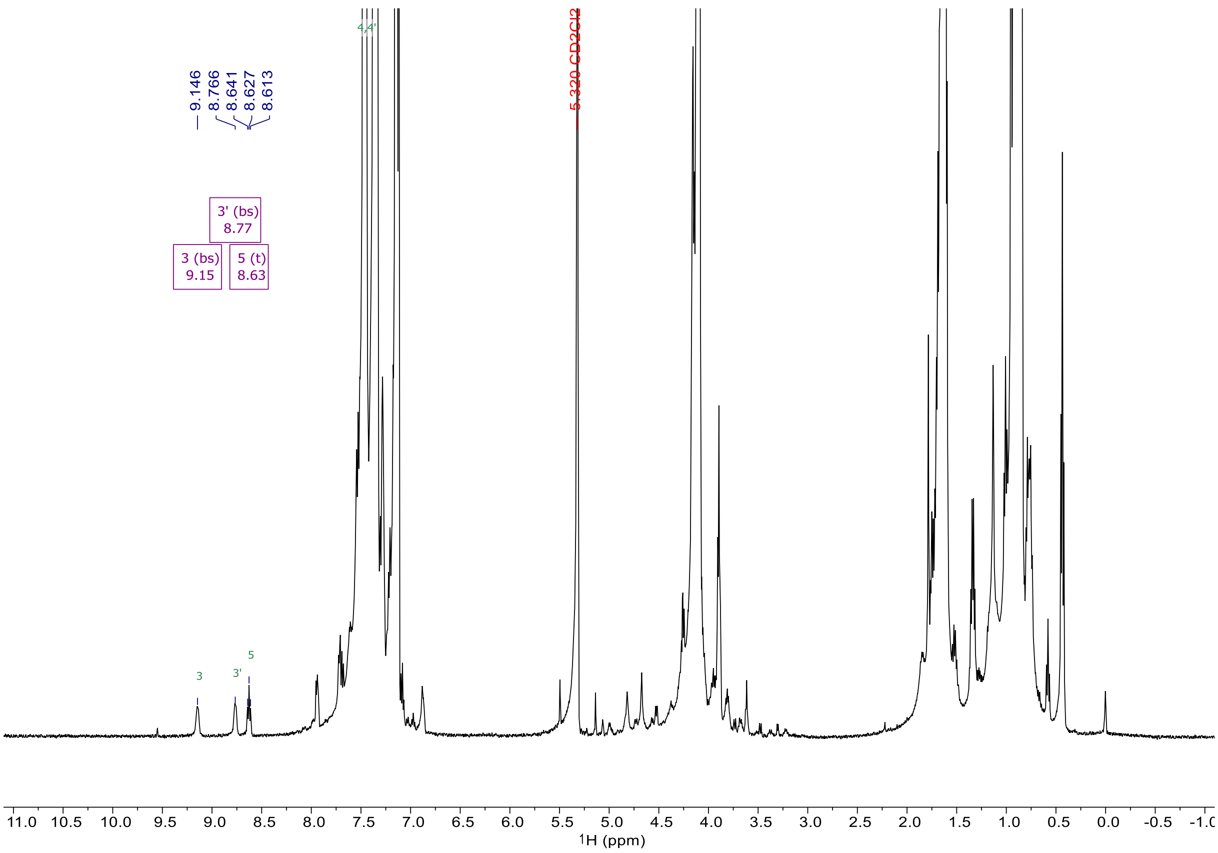

Figure S9. ^{1}H NMR spectrum of the reaction mixture at 183K. Labelled peaks show the characteristic signals assigned to the carbene **15[O$_3$N]**.

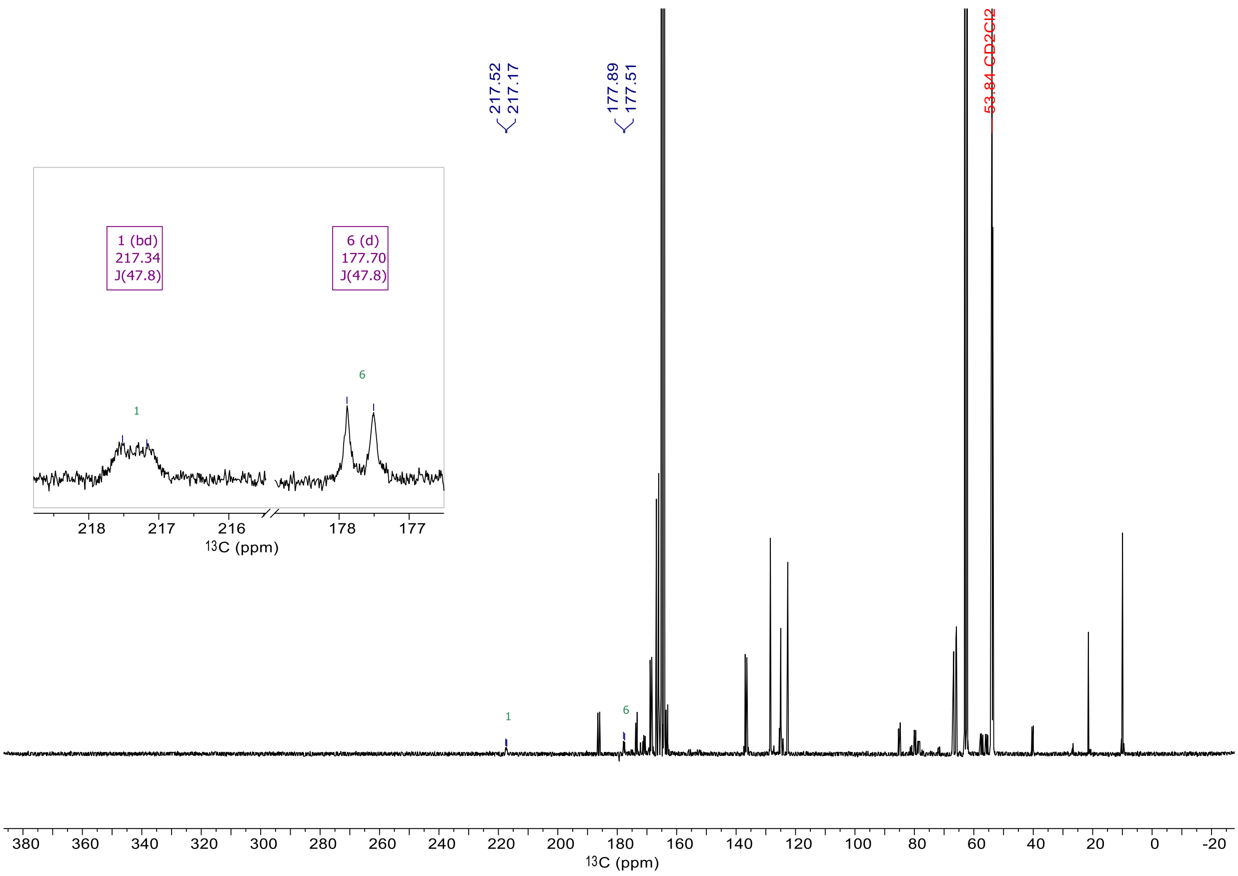

Figure S10. ^{13}C NMR spectrum of the reaction mixture at 183K; the labeled peaks show the characteristic signals assigned to the carbene **15[O$_3$N]**

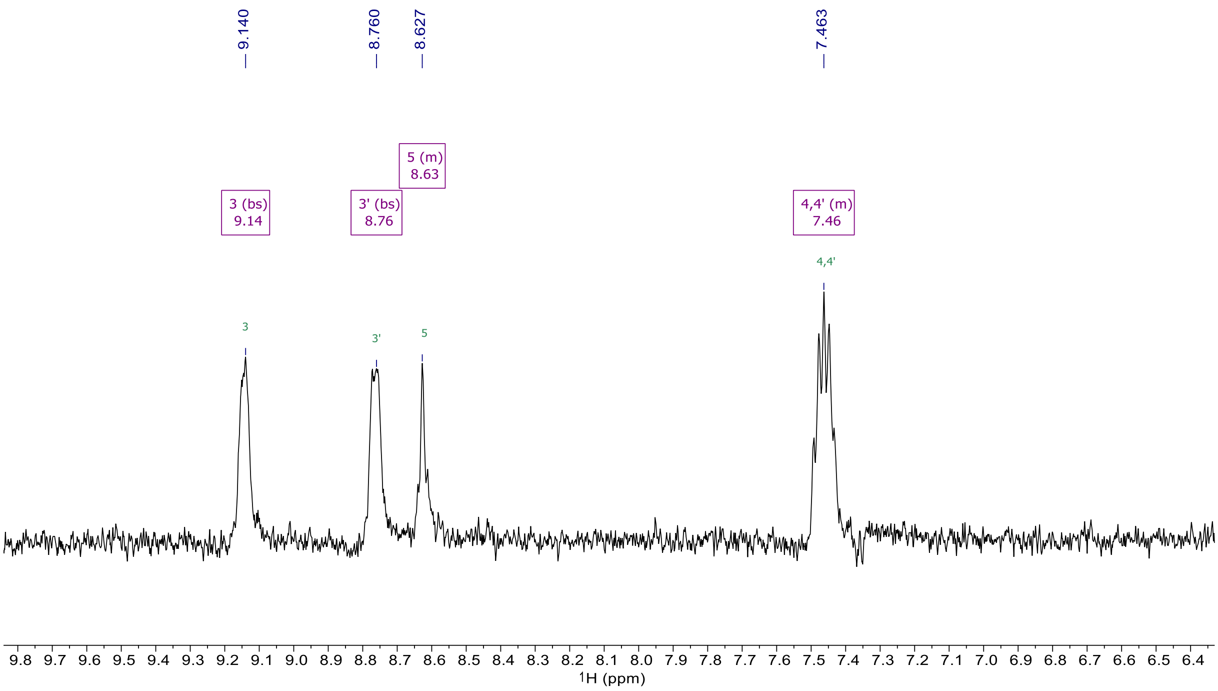

Figure S3. 1D selective TOCSY spectrum showing the signals of the Ph spin system of carbene **15[O$_3$N]**

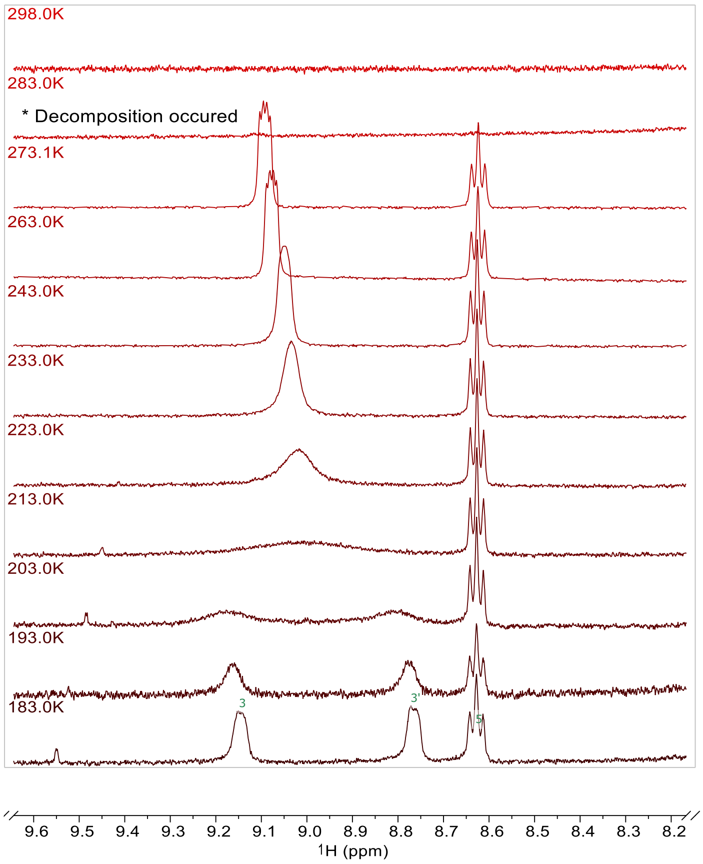

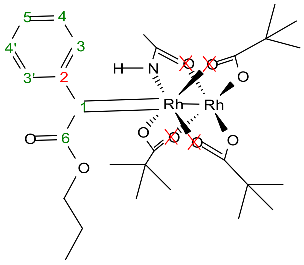

T_c	213	K
Δu	194	Hz

$k_{ex} = \pi \times \Delta u/\sqrt{2}$

$k_{ex} =$ 430.96 hz

physical constants

	**value**	**unit**
R	8.31446	J/(K*mol)
h	6.63E-34	J s
k_B	1.4E-23	J K^{-1}

$\Delta G^{\ddagger}_{Tc} = -\ln(k_{ex} \times h / (k_B \times T_C)) \times RT$

$\Delta G^{\ddagger}_{Tc}$ =	40.8	kJ mol^{-1}
	9.8	kcal mol^{-1}

Figure S4. Left: Variable Temperature ^{1}H NMR spectra of carbene **15[O₃N]** showing the characteristic region of signals H3, H3' and H5. Right: Extracted values, formula and constants used to estimate the rotational barrier about the C1-C2 bond; labeling as shown in the Insert.

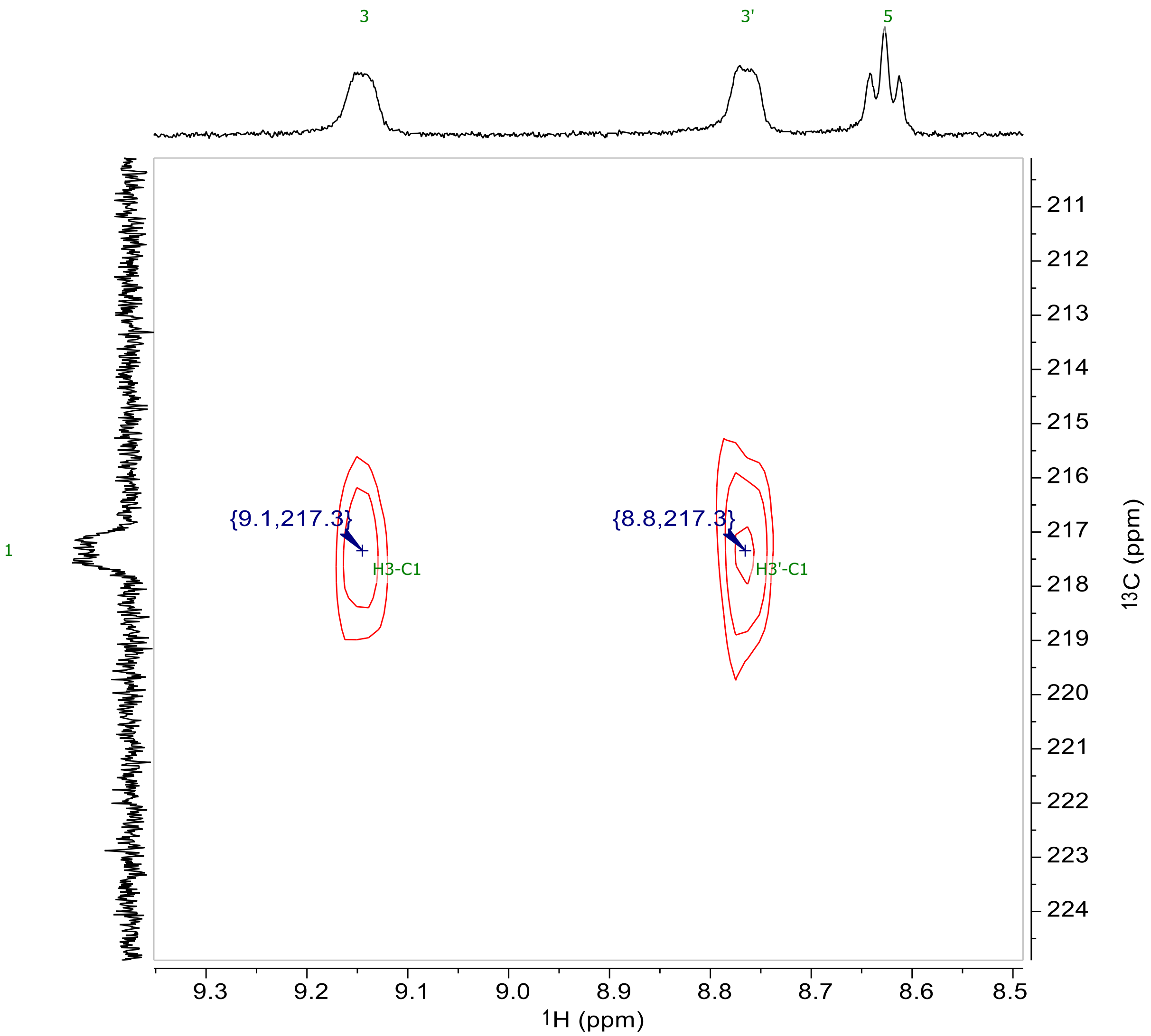

Figure S5. ^{1}H-^{13}C HMBC spectrum at 183K showing the cross peak to the carbene signal of **15[O$_3$N]**

NMR Spectra of New Compounds

S1: ^{1}H NMR (400 MHz, CD_3CN):

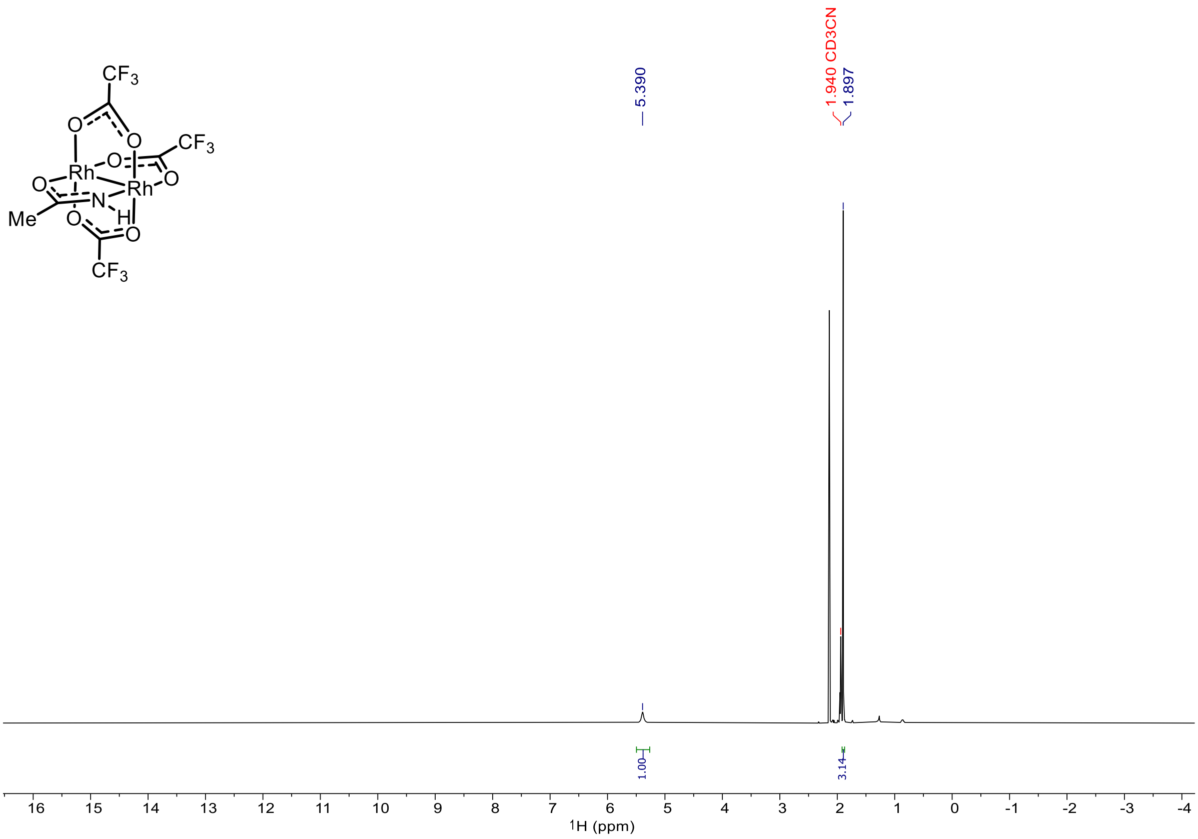

S1: ^{13}C NMR (151 MHz, CD_3CN):

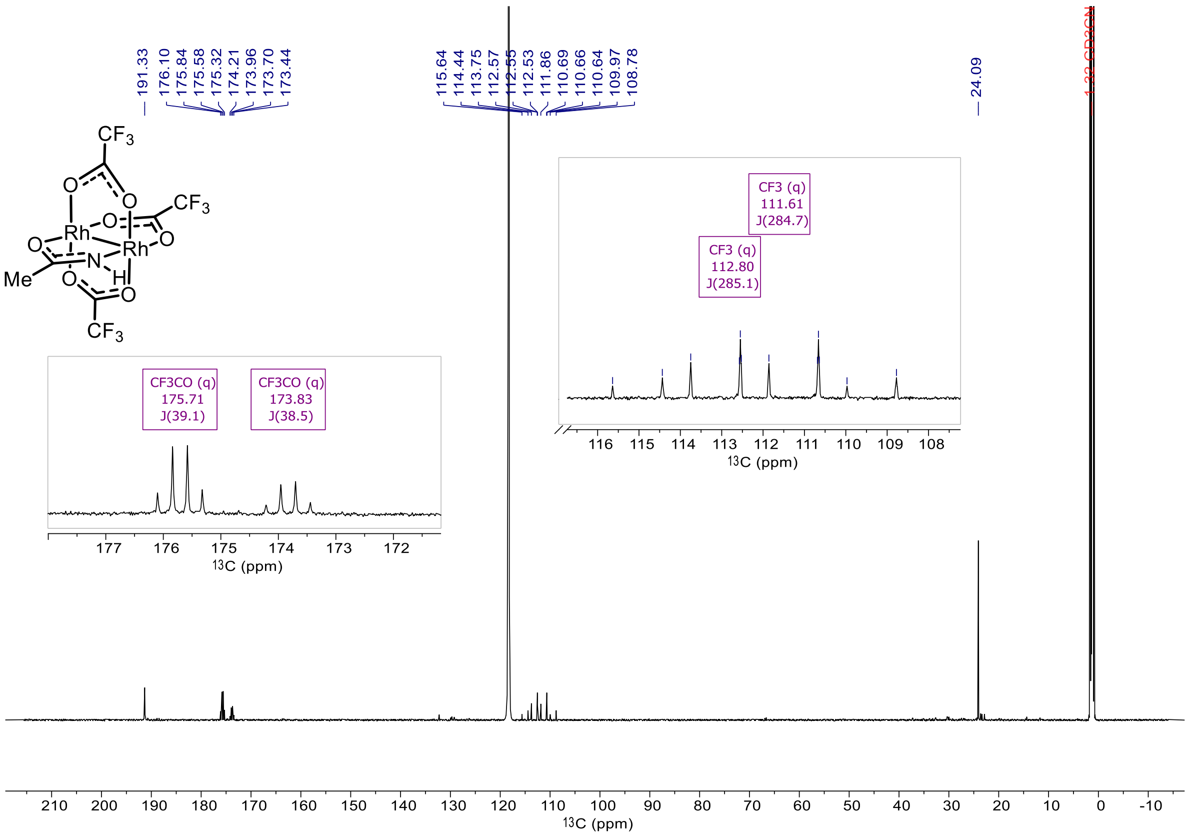

S1: ^{19}F NMR (565 MHz, CD_3CN):

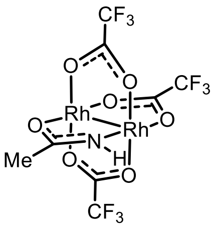

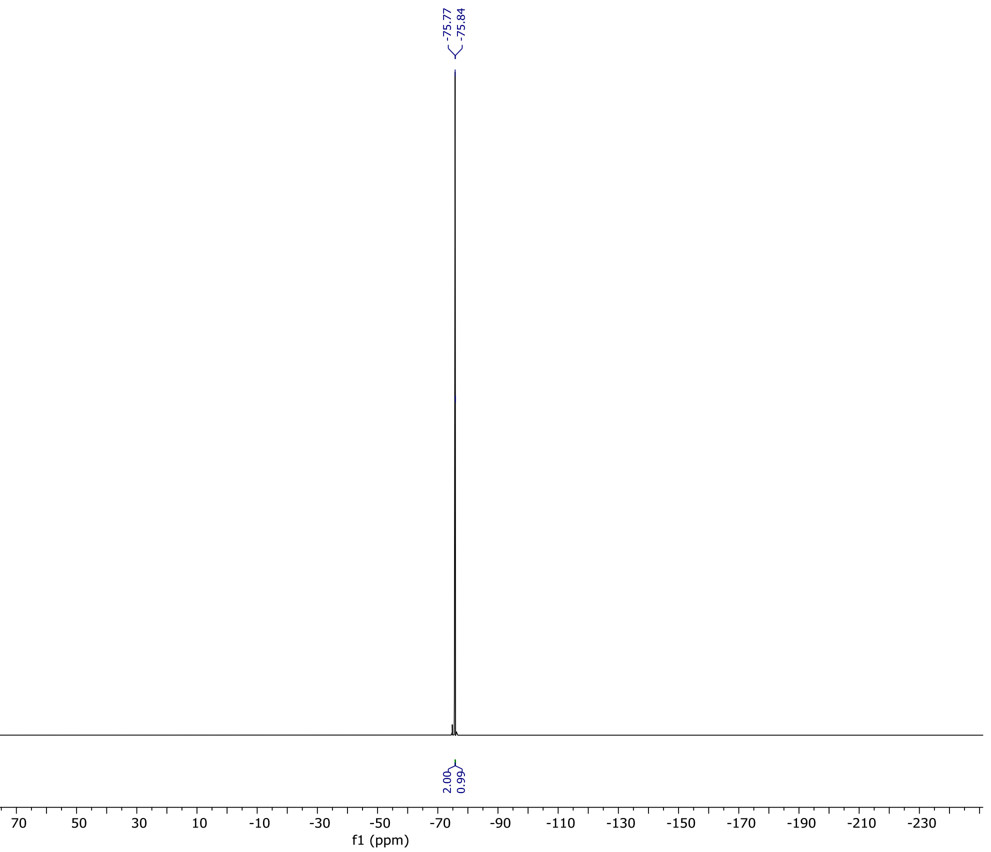

C9: ^{1}H NMR (600 MHz, CD_3CN):

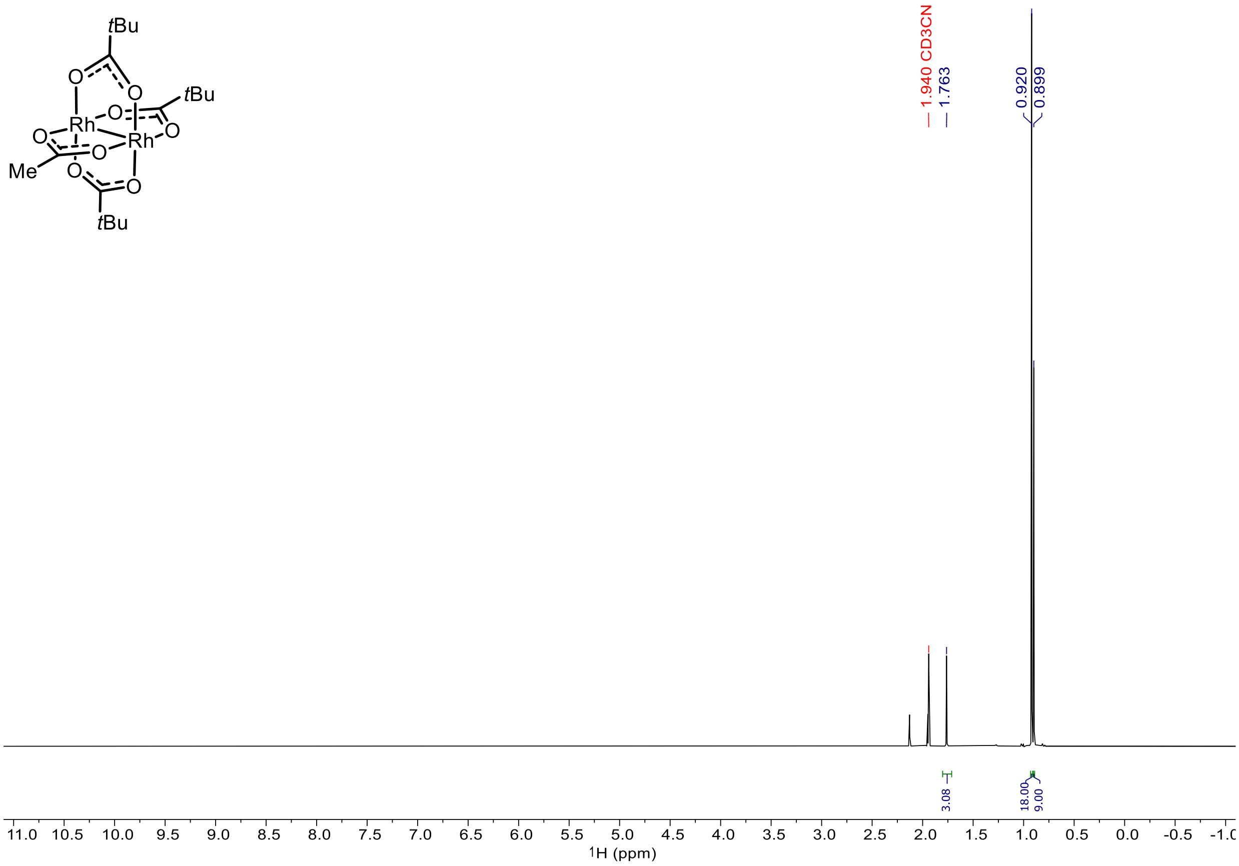

C9: ^{13}C NMR (151 MHz, CD_3CN):

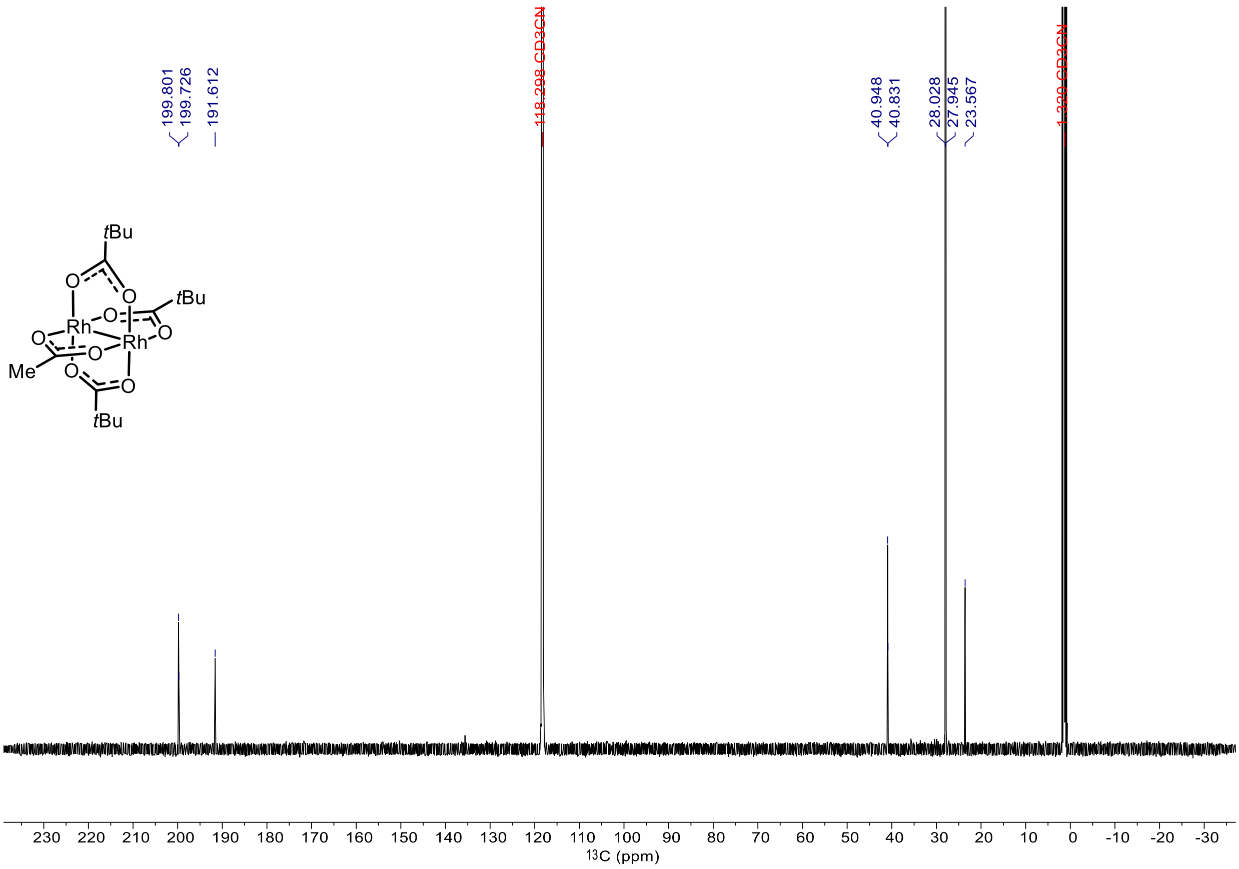

C9: H(C)Rh NMR (499.9 MHz, 15.9 MHz, CD_3CN)

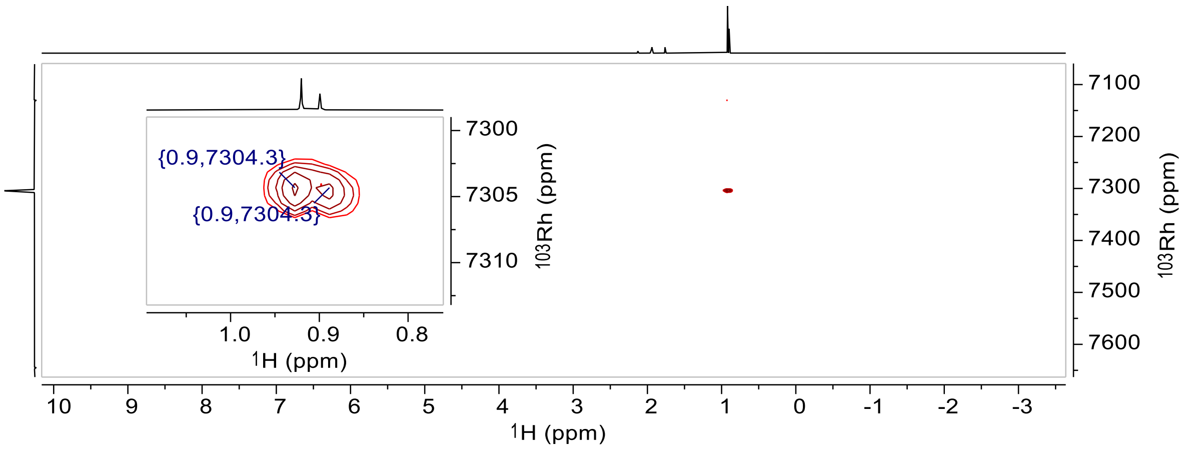

C10: ^1H NMR (600 MHz, CD_3CN):

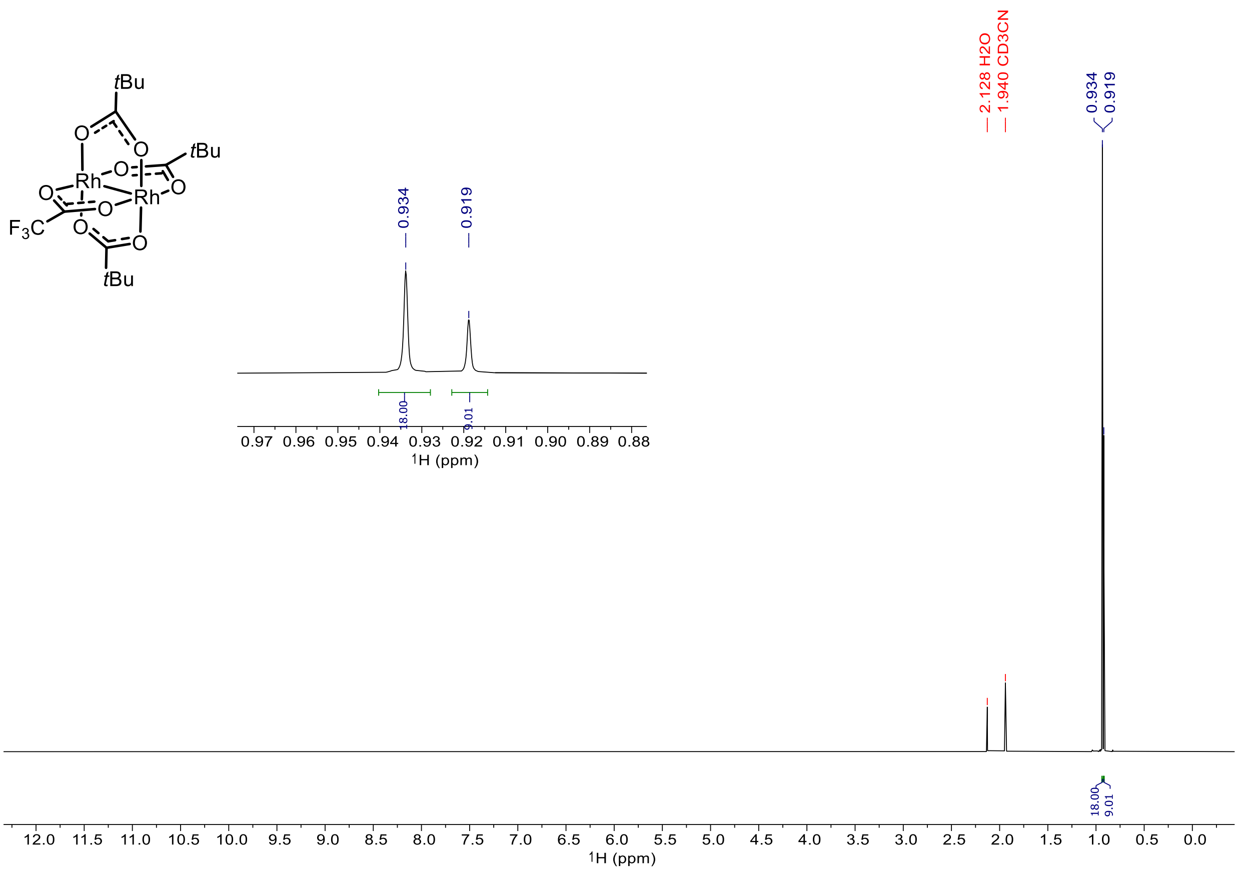

C10: ^{13}C NMR (151 MHz, CD_3CN):

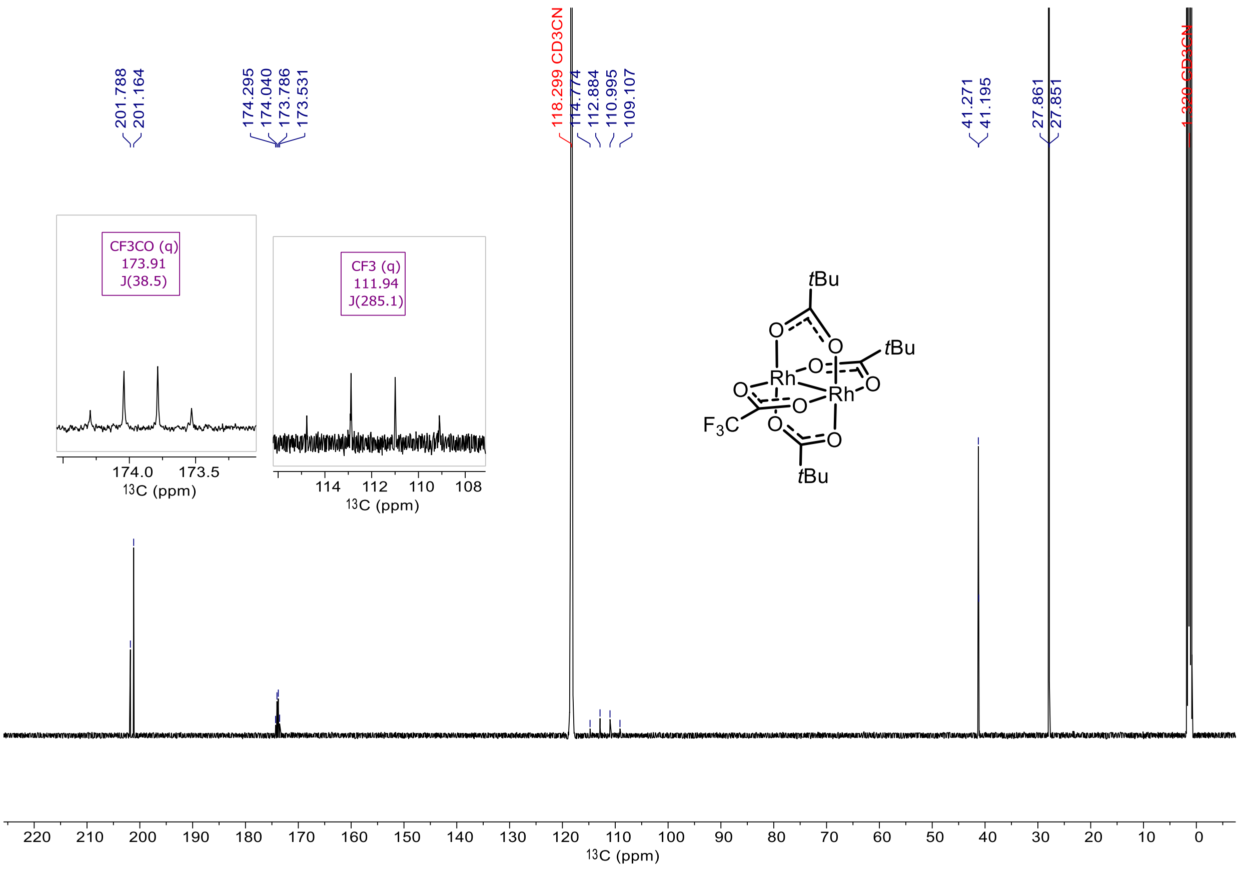

C10: ^{19}F NMR (565 MHz, CD_3CN):

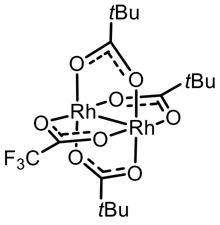

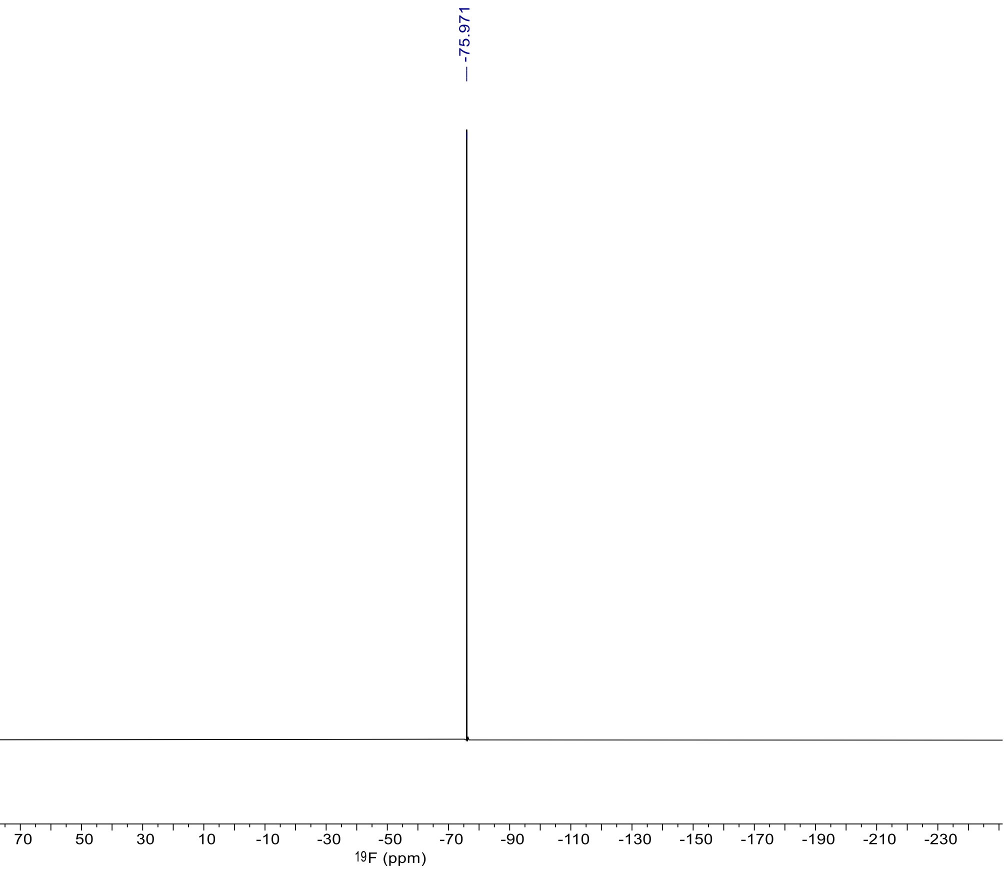

C10: H(C)Rh NMR (499.9 MHz, 15.9 MHz, CD_3CN)

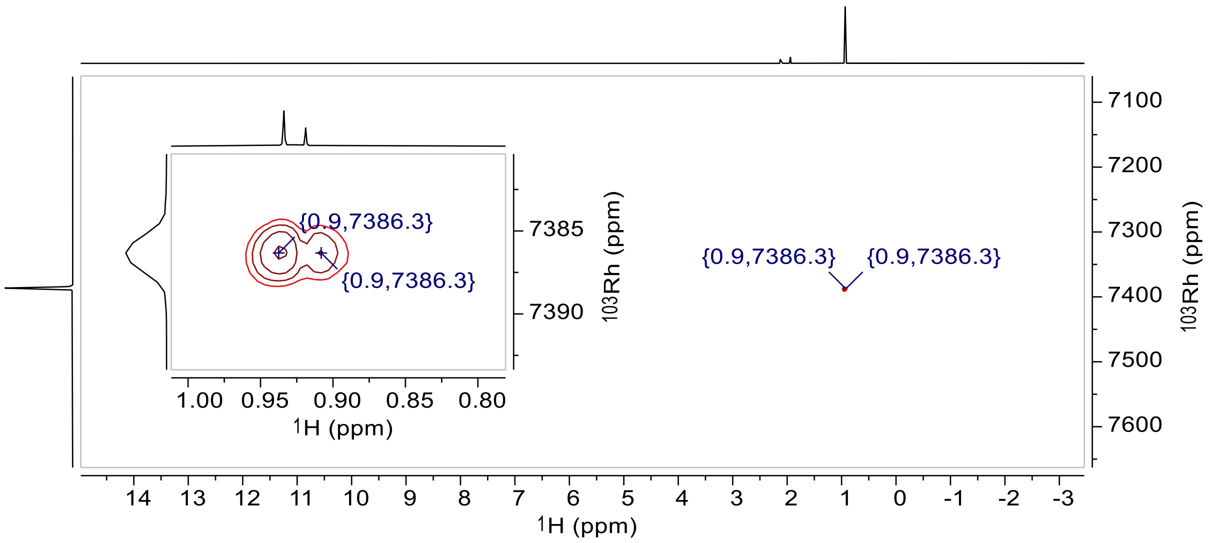

[^{13}C]$_2$-S2: ^{1}H NMR (400 MHz, $CDCl_3$):

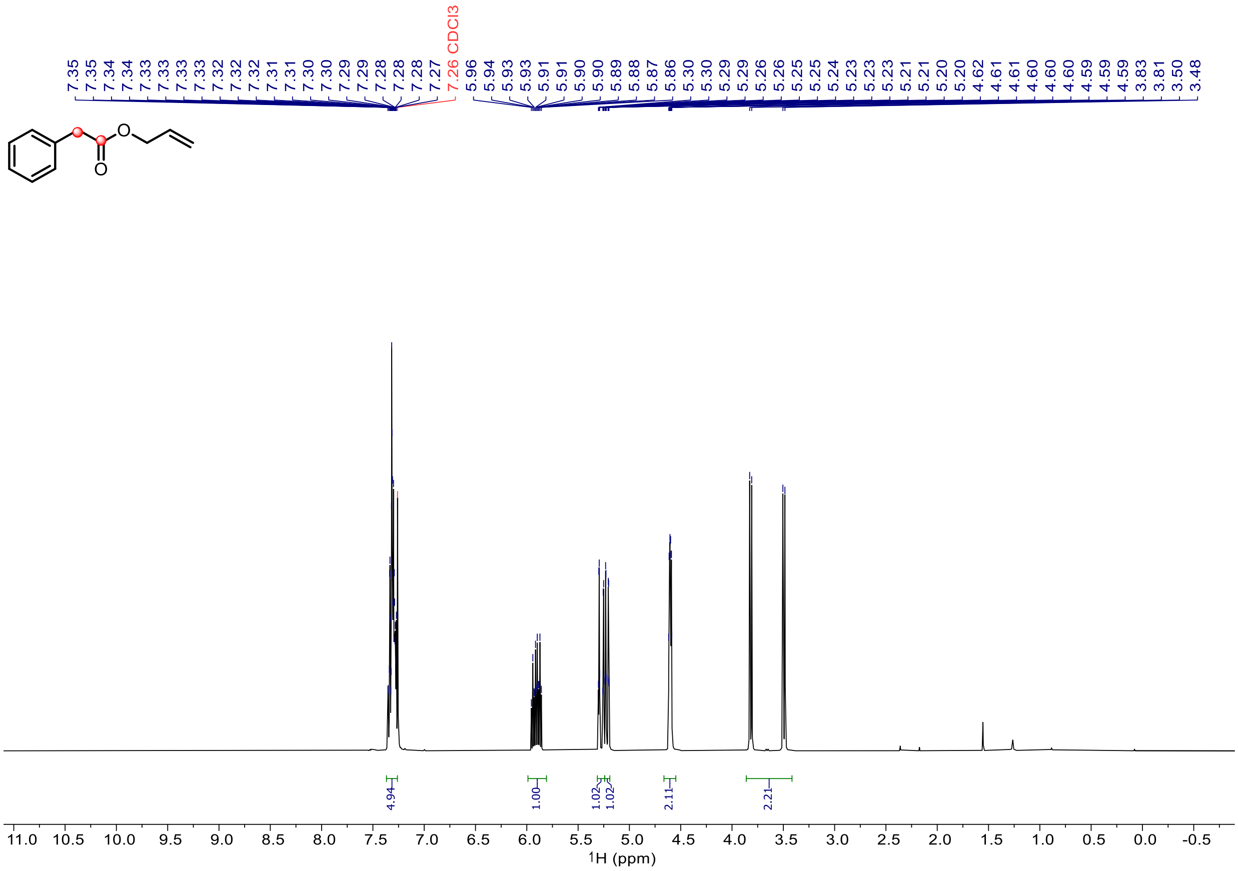

[^{13}C]$_2$-S2: ^{13}C NMR (101 MHz, $CDCl_3$):

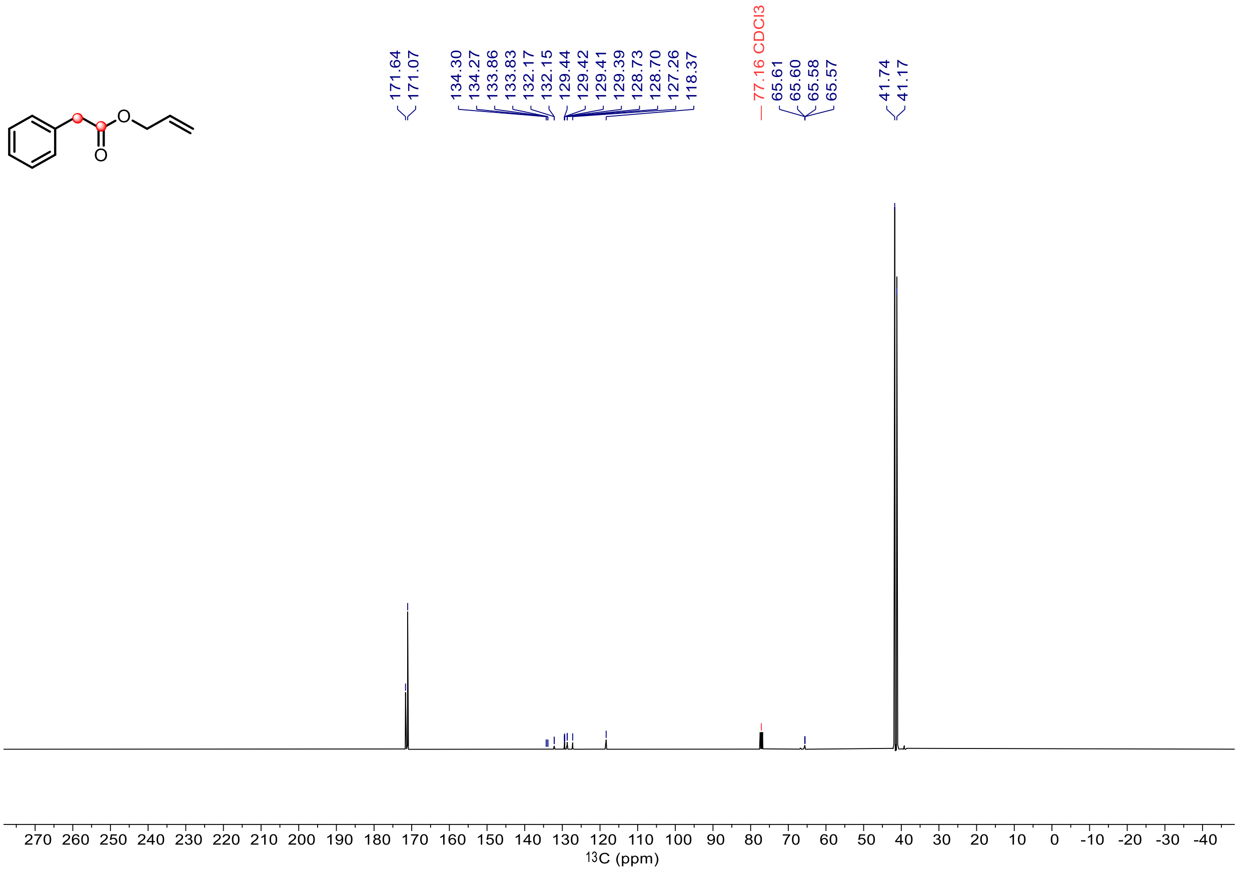

[13C]2-S3: ^{1}H NMR (400 MHz, $CDCl_3$):

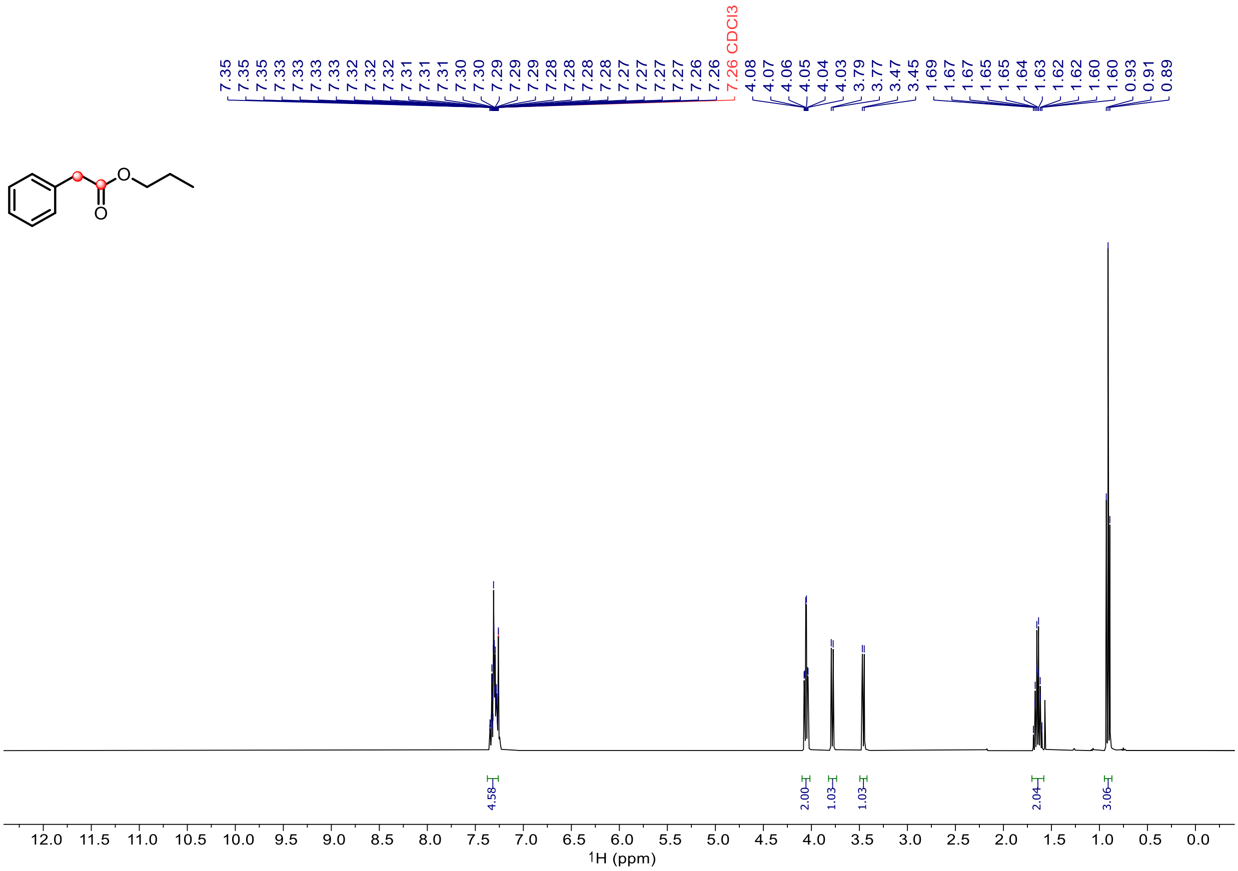

[13C]2-S3: ^{13}C NMR (101 MHz, $CDCl_3$):

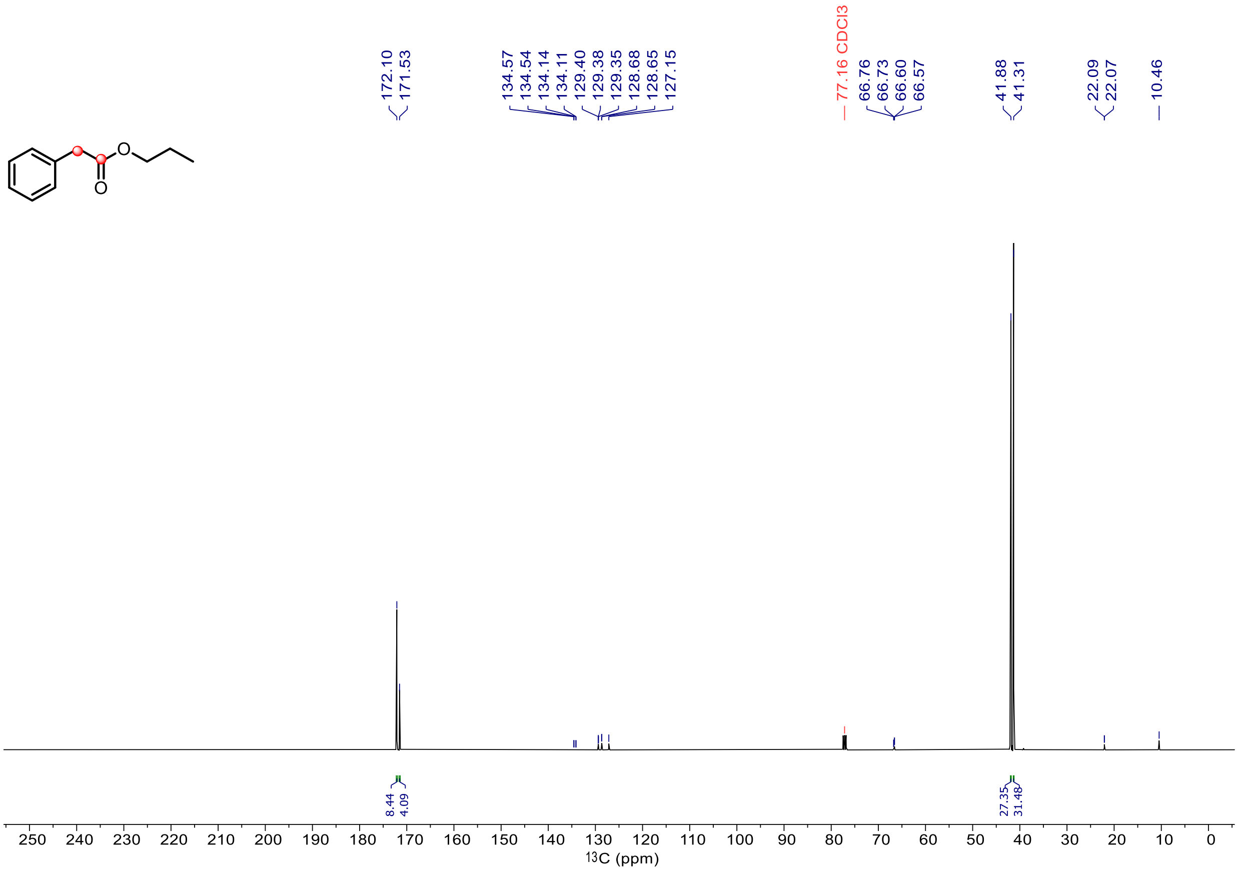

[^{13}C]$_2$-**C14**: ^{1}H NMR (400 MHz, $CDCl_3$):

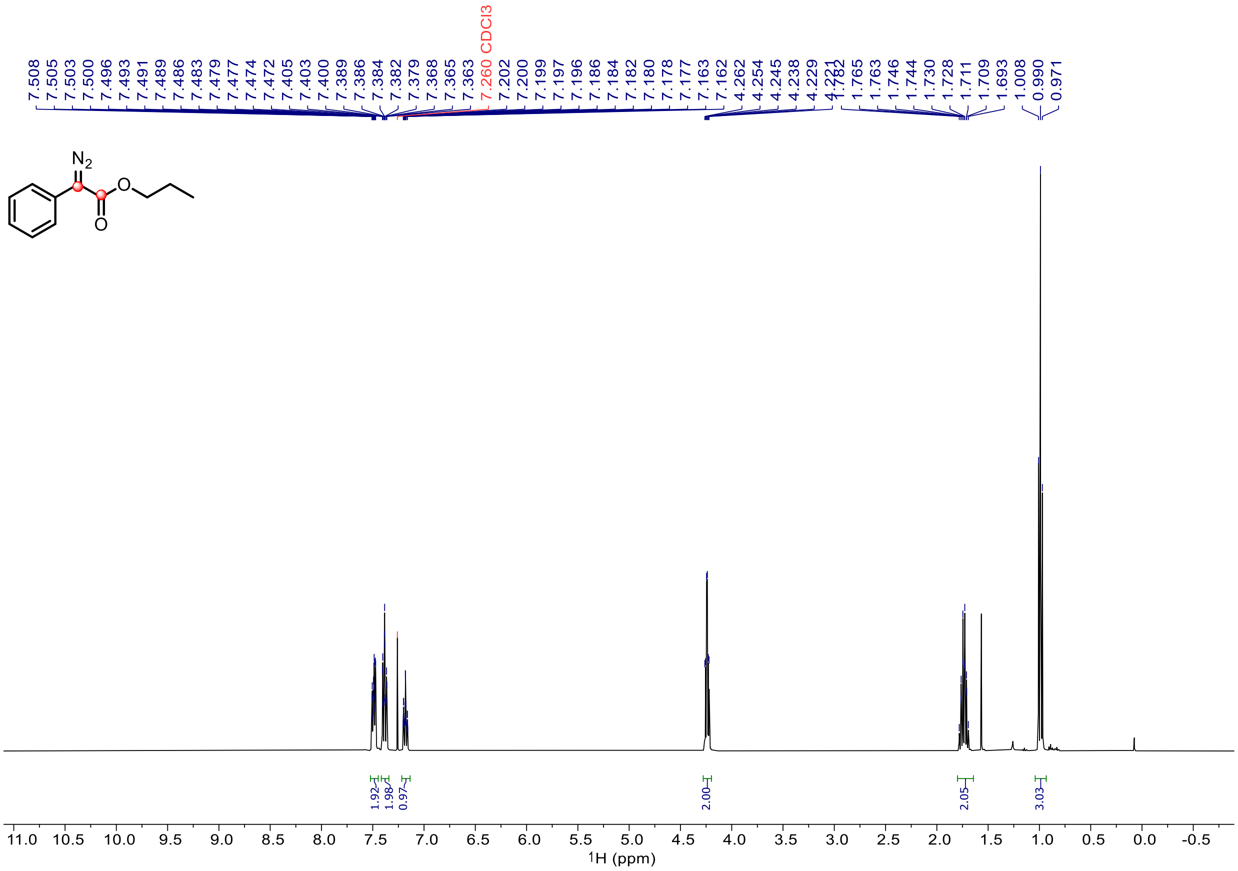

[^{13}C]$_2$-**C14:** ^{13}C NMR (101 MHz, $CDCl_3$):

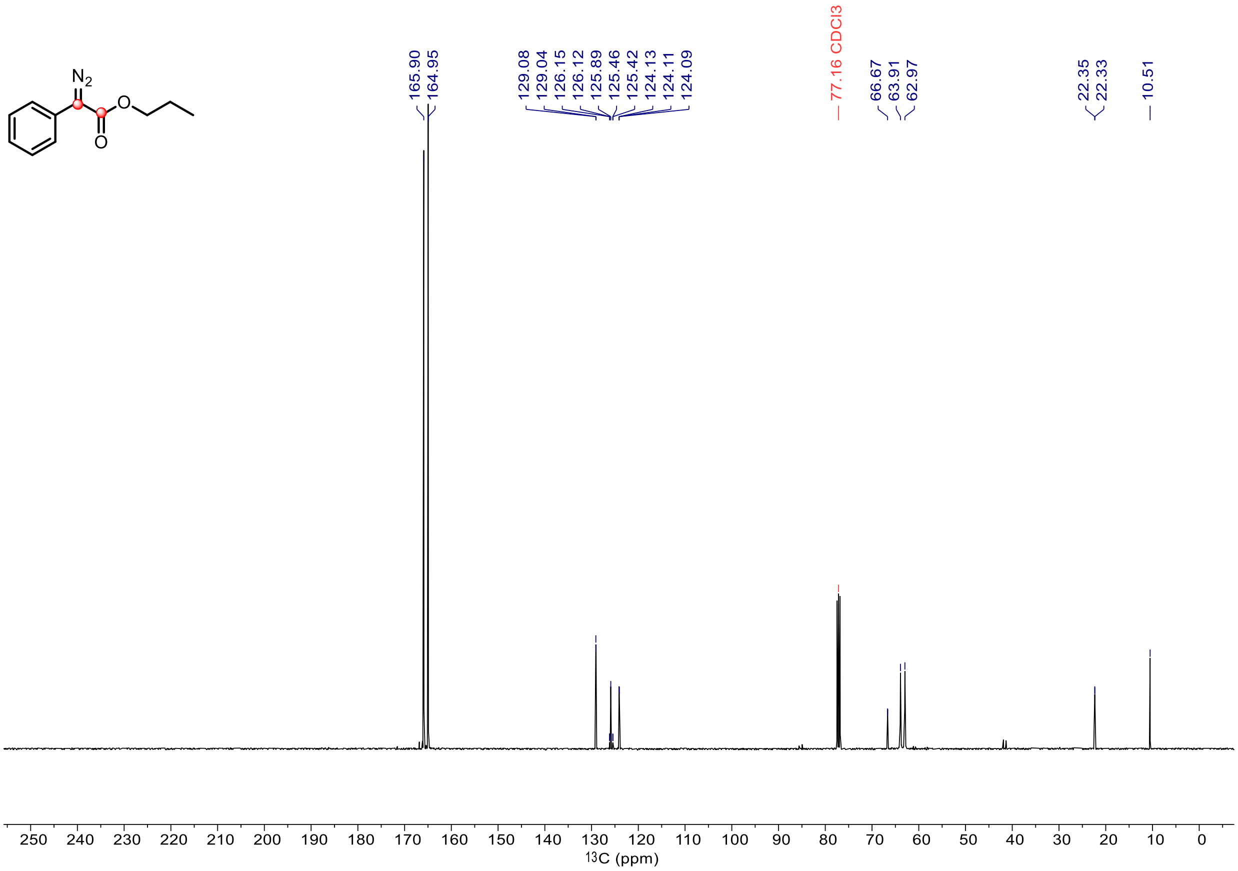

[^{13}C]$_2$-C6: ^{1}H NMR (400 MHz, $CDCl_3$):

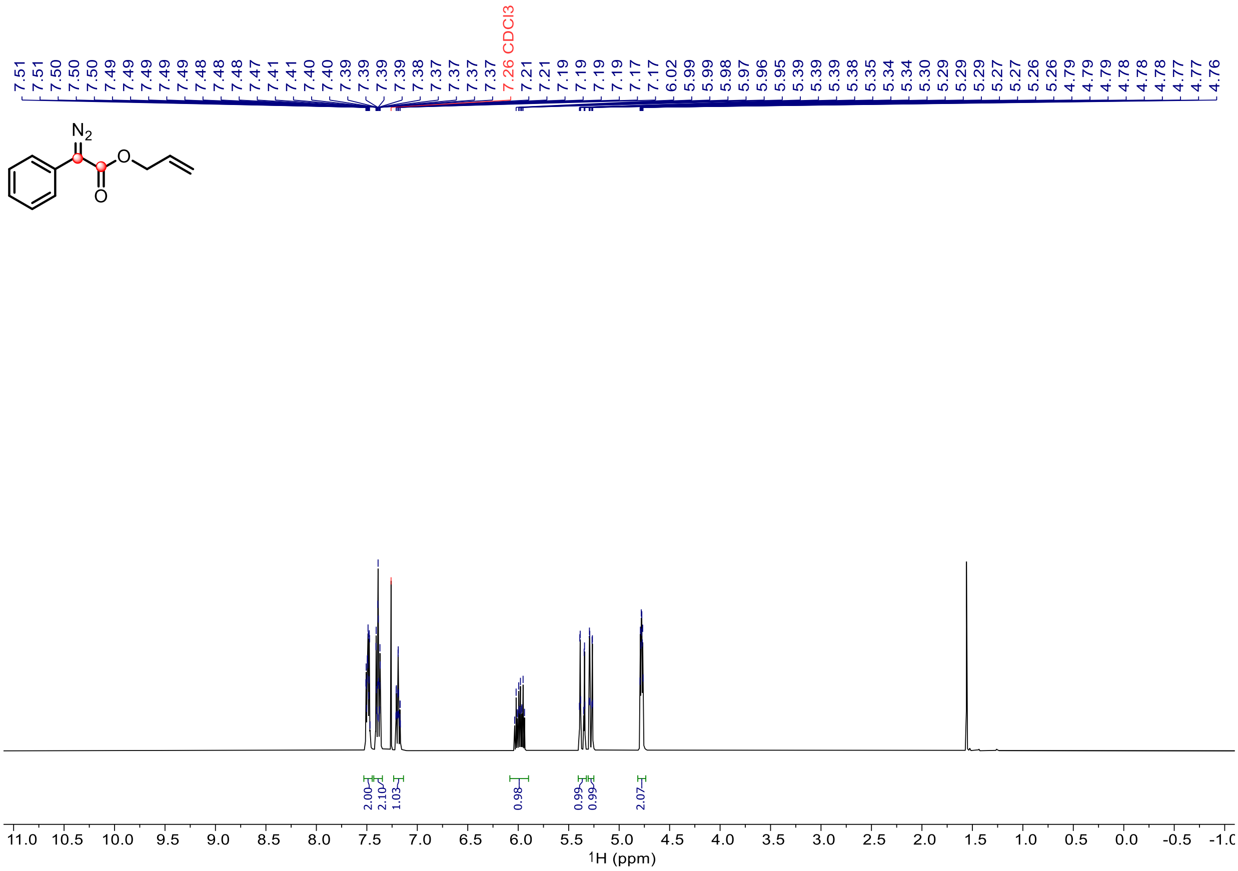

[^{13}C]$_2$-C6: ^{13}C NMR (101 MHz, $CDCl_3$):

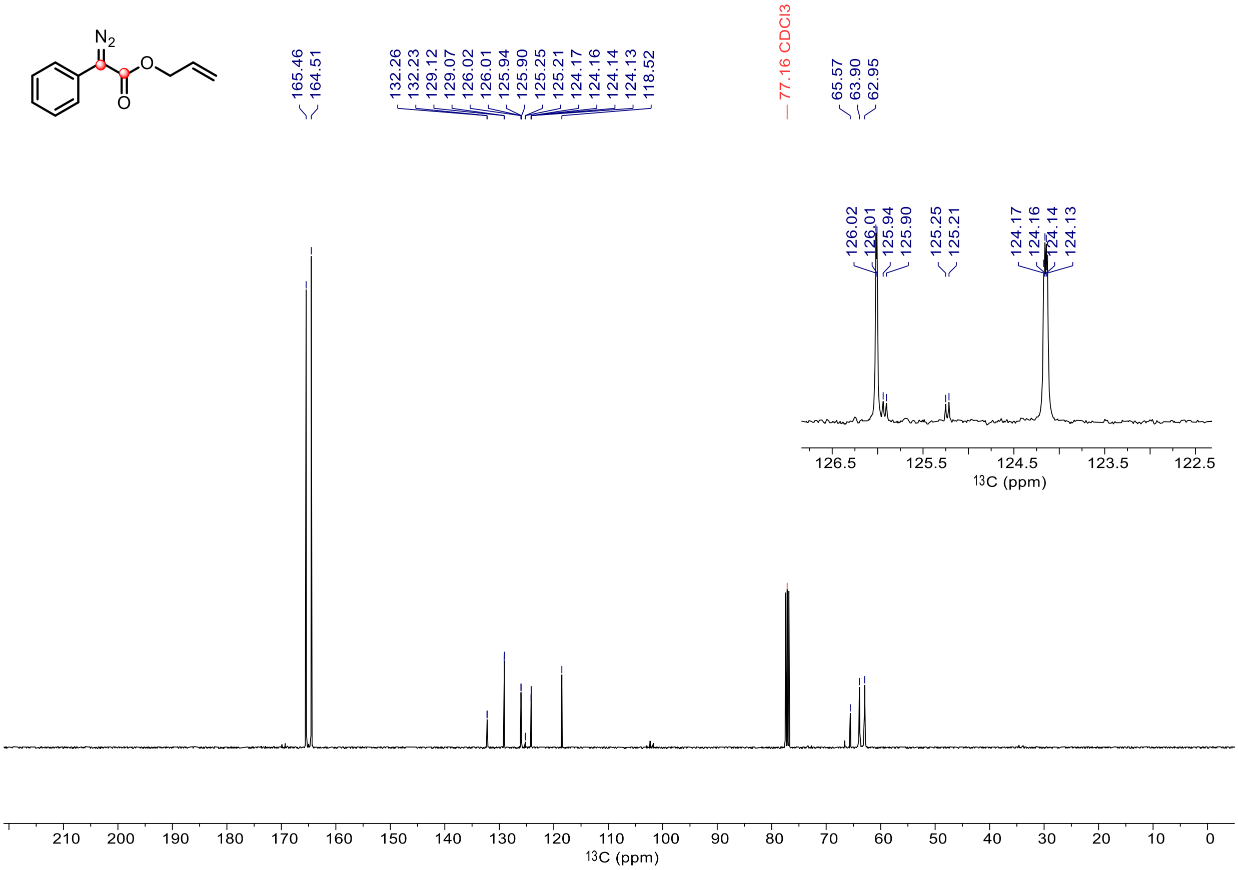

References

[1] X. Gui, D. Sorbelli, F. P. Caló, M. Leutzsch, M. Patzer, A. Fürstner, G. Bistoni, A. A. Auer, *Chem. Eur. J.* **2024**, *30*, e202301846.

[2] D. M. Upp, R. Huang, Y. Li, M. J. Bultman, B. Roux, J. C. Lewis, *Angew. Chem. Int. Ed.* **2021**, *60*, 23672-23677.

[3] F. P. Caló, A. Fürstner, *Angew. Chem. Int. Ed.* **2020**, *59*, 13900-13907.

[4] Michael P. Doyle, W. Hu, *Advanced Synthesis & Catalysis* **2001**, *343*, 299-302.

[5] G. R. Fulmer, A. J. M. Miller, N. H. Sherden, H. E. Gottlieb, A. Nudelman, B. M. Stoltz, J. E. Bercaw, K. I. Goldberg, *Organometallics* **2010**, *29*, 2176-2179.

[6] F. P. Caló, G. Bistoni, A. A. Auer, M. Leutzsch, A. Fürstner, *J. Am. Chem. Soc.* **2021**, *143*, 12473-12479.

[7] E. D. B. Robin K. Harris, Sonia M. Cabral de Menezes, Pierre Granger, Roy E. Hoffman and Kurt W. Zilm, *Pure Appl. Chem.* **2008**, *80*, 59-84.

[8] J. Burés, *Angew. Chem. Int. Ed.* **2016**, *55*, 16084-16087.
