# Supplementary material for: In Situ Observation of Elusive Dirhodium Carbenes and Studies on the Innate Role of Carboxamidate Ligands in Dirhodium Paddlewheel Complexes: A Combined Experimental and Computational Approach
Source: J Am Chem Soc. 2024 Sep 11;146(38):26466–77. doi: 10.1021/jacs.4c09847 (PMC11440507; doi:10.1021/jacs.4c09847)
Supplement: Supplementary file 2 — ja4c09847_si_002.pdf [file ja4c09847_si_002.pdf]

SUPPORTING INFORMATION - PART 2

COMPUTATIONAL DETAILS

The In Situ Observation of Elusive Dirhodium Carbenes and Studies on The Innate Role of Carboxamidate Ligands in Dirhodium Paddlewheel Complexes.

A Combined Experimental and Computational Approach

Matthias Peeters,[a] Lorenzo Baldinelli,[b] Markus Leutzsch,[a] Fabio Caló,[a] Alexander Auer,[a] Giovanni Bistoni,[b],* and Alois Fürstner[a],*

[a] *Max-Planck-Institut für Kohlenforschung, D-45470 Mülheim/Ruhr, Germany*

[b] *University of Perugia, Department of Chemistry, Biology and Biotechnology, Via Elce di Sotto 8, I-06123 Perugia, Italy*

Emails: fuerstner@kofo.mpg.de; giovanni.bistoni@unipg.it

Table of Contents

Mechanism	S2
NMR Shifts	S2
Structures	S6
References	S33

Unless otherwise specified, all calculations were performed with ORCA 5.0.3.[1]

Mechanism

The computational protocol used in this work is consistent with that successfully adopted in previously published mechanistic studies on related transformations.[2],[3] In the present case, different computational settings were compared and the results found to be largely independent of the technical parameters of the calculations. The reaction energetics obtained with different basis sets, exchange correlation functionals and implicit solvation models for the reaction pathways at the [O_3N]-face and [O_4]-face are shown in Figures S1 and S2.

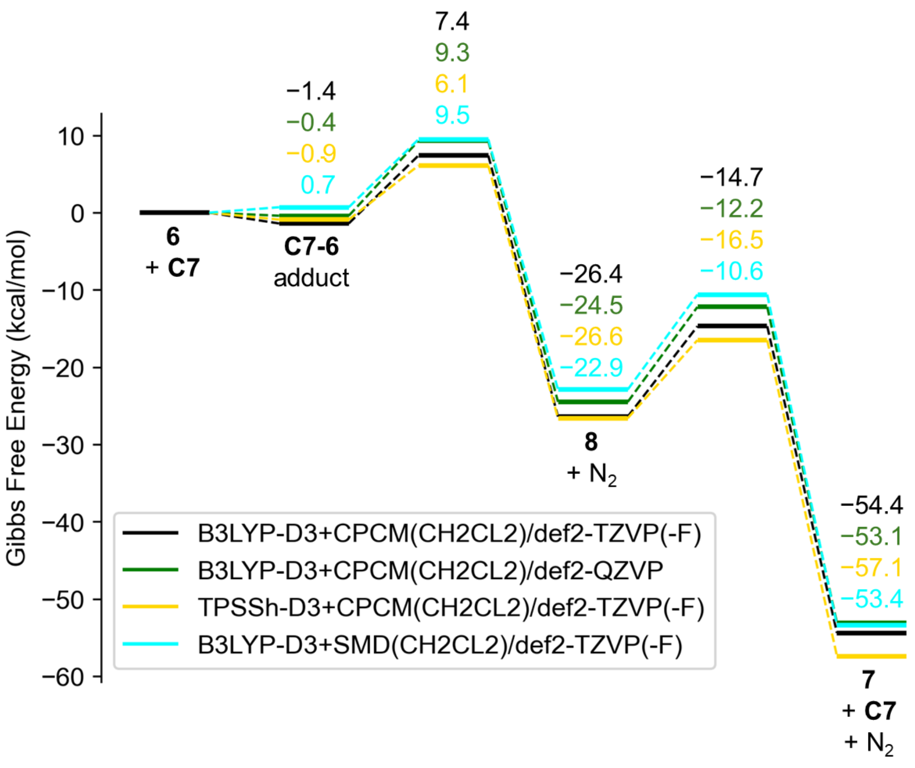

Figure S1. Effect of the basis set, functional, set and implicit solvation model on the reaction profile on the [O_3N]-face. Geometries and free energies corrections were computed at B3LYP-D3+CPCM(CH2CL2)/def2-SVP level.

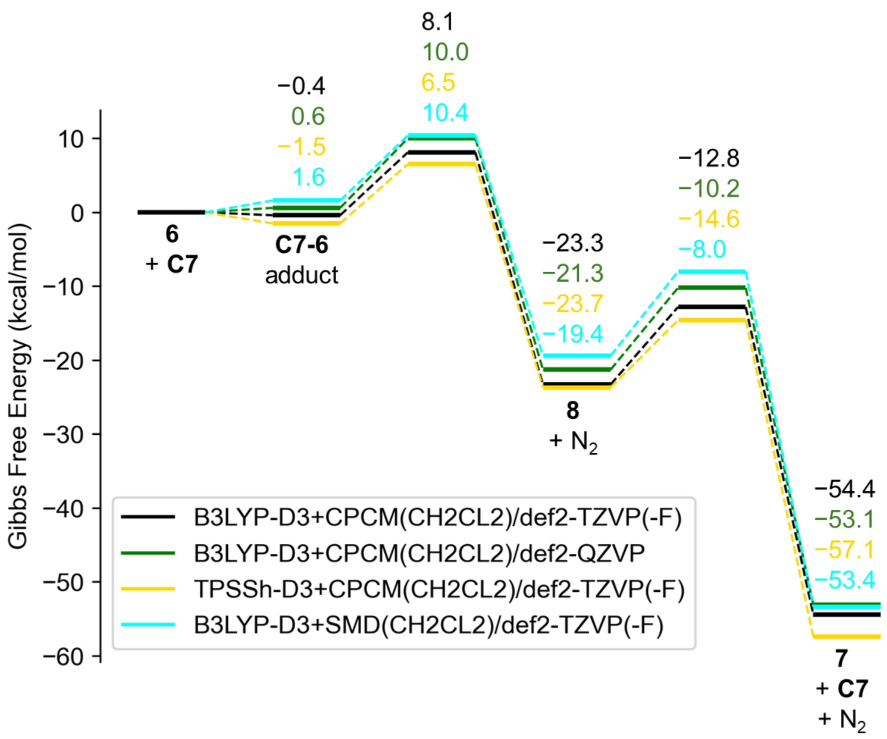

Figure S2. Effect of the basis set, functional, and implicit solvation model on the reaction profile on the [O_4]-face. Geometries and free energies corrections were computed at B3LYP-D3+CPCM(CH2CL2)/def2-SVP level.

A preliminary exploration of the chemical space for the relevant reaction intermediates was carried out using the CREST algorithm at the XTB2 level.[4],[5] Refined geometries, frequencies and thermostatistical corrections at 298.15 K were computed using the hybrid exchange-correlation functional B3LYP-D3(BJ) together with Alrichs def2-SVP basis set.[6],[7],[8],[9],[10],[11] Electronic energies were refined at B3LYP-D3(BJ)/def2-TZVP(-f) level. In all calculations, solvent effects were included using the C-PCM implicit solvation model for CH_2Cl_2.[12],[13] The resolution of identity (RI) approximation was used in the RIJCOSX variant together with the corresponding auxiliary basis set.[14],[15],[16] Initial guesses for the transition state (TS) structures were found with NEB and relaxed scan calculations.[17],[18] Final TS structures were then obtained through fully relaxed geometry optimizations. The low-energy stationary points were characterized by calculating vibrational modes.

Transition states showed a single imaginary frequency, while reaction intermediates showed no imaginary vibrational frequency.

NMR Shifts

Multiple computational setups (see Table S1) were tested to compute the ^{13}C NMR shifts for **8[O$_4$]** and for **8[O$_3$N]** for the heteroleptic catalyst and **8[O$_4$]** for the homoleptic catalyst. Tetramethylsilane (TMS) was used as reference compound in all NMR calculations.

Regardless of the computational settings used, **8[O$_3$N]** consistently resonates at lower frequencies than **8[O$_4$]**, which aligns with the experimental findings. When TMS was used as a reference, the computational approach that provided the best quantitative agreement with experimental data was a composite method. This method combines scalar relativistic single-point NMR calculations at the TPSSh/ZORA-def2-TZVPP + CPCM level using ORCA 5.0.3,[19],[20],[21],[22] with corrections for the spin-orbit coupling (SOC) effect[23] calculated at PBE0 level using the Amsterdam Density Functional (ADF) 2017 software.[24],[25]

For the benchmark study, B3LYP, TPPSh and PBE0[26] exchange-corrrelation functionals were tested in conjunction with def2-TZVPP basis set in ORCA (denoted hereafter as TZVPP) and TZ2P basis set in ADF.[27] For the calculations in ADF, the basis set TZ2P was used and solvent effects were included using the COSMO implicit solvation model.[28]

Table S1. NMR shifts of the carbenic carbon for the intermediates **8[O3N]** and **8[O4]** derived from the heteroleptic catalyst **C7** (blue), and for the analogous **8[O4]** intermediate derived from the homoleptic catalyst **C6** (red) using different computational setups. The table also reports the differences between the shifts of **8[O4]** for both homoleptic (red) and heteroleptic (blue) catalysts with respect to the shift of **8[O3N]**.

8[O_3N] 8[O_4] 8[O_4]

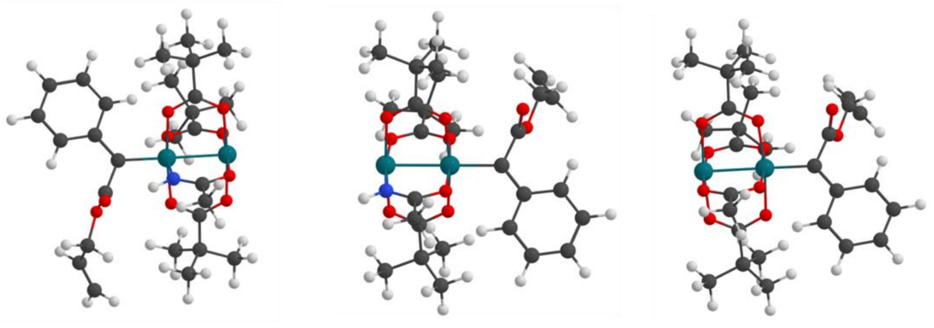

FUNCTIONAL / BASIS SET	ZORA	SOC	$\delta_{8[O4]} - \delta_{8[O3N]}$	$\delta_{8[O4]} - \delta_{8[O3N]}$	$\delta_{8[O3N]}$	$\delta_{8[O4]}$	$\delta_{8[O4]}$
**B3LYP/TZVPP**	NO	NO	8.8	11.1	295.6	304.4	306.7
**PBE0 / TZVPP**	NO	NO	9.0	11.0	306.2	315.3	317.2
**PBE0 / TZVPP**	YES	NO	9.5	11.3	306.3	315.8	317.6
**TPSSH/TZVPP**	YES	NO	8.4	10.2	251.7	260.1	261.9
**TPSSH/TZVPP DECONTRACTED BASIS**	YES	NO	8.1	9.8	255.6	263.6	265.3
**TPSSH/TZVPP**	YES	YES*	8.1	10.5	227.0	235.1	237.6

*SOC effects were computed with ADF 2017 program package, as described above.

Structures

C7

58

C	-2.63316316521483	-0.38178135111714	0.00224939284275
O	-2.03821473751161	-0.40539235973380	1.12420890301306
Rh	0.00709711881278	-0.42146581147657	1.18388657541255
Rh	-0.00540479399868	-0.50520145152194	-1.19595896349898
N	0.00199596915584	-2.51479318015804	-1.01446185104235
C	0.00925510901034	-3.10854093264241	0.15300024181893
O	0.01206811687063	-2.45435043680458	1.26206878369263
C	0.01500743850127	-4.60928171130110	0.26999397538963
H	0.00942347924564	-5.09977310658162	-0.71260208621692
H	-0.86755806802501	-4.92982246952038	0.84502949585927
H	0.90817608098791	-4.92454893734787	0.83150700304911
H	0.00141614473082	-3.13200289527393	-1.82374832194443
O	-2.05007156759151	-0.46704730589784	-1.12232666530870
O	2.04023988184472	-0.45770874261307	-1.14421373503338
C	2.63502242735453	-0.37533309469140	-0.02626490959173
O	2.05167162579789	-0.39701180787513	1.10253057764659
O	-0.01182660763613	1.57728446015611	-1.18768057642521
C	-0.00758149606300	2.19473321098586	-0.08344210873359
O	0.00043915117121	1.62599355064309	1.05530270818599
C	4.15217763745343	-0.17182263446852	-0.02307445933032
C	4.74667598974096	-0.46782772698771	-1.40582991115590
C	4.78868602068606	-1.08529428710072	1.03917823259680
C	4.38499574383400	1.31161545597671	0.34486187210990
H	4.56136702036361	-1.51124360414737	-1.70437998515098
H	5.83550639939330	-0.30502101246340	-1.37866727985972
H	4.31753201210901	0.18938749031227	-2.17558486345924
H	4.60592791944891	-2.14702559620523	0.80812066481446

H	4.38313294213972	-0.87389291883607	2.03849342566930
H	5.87757089239885	-0.92194376883631	1.06187686548631
H	5.46547081076161	1.52529806046916	0.35319532377415
H	3.97424431869512	1.54109912449460	1.33920274252393
H	3.90696785115669	1.97902713490622	-0.39001058377472
C	-0.01301525351282	3.72656630509167	-0.08843977634965
C	1.25260494313981	4.20641809859227	0.65100281984079
C	-1.27510469375643	4.19676669730282	0.66323231483266
C	-0.02234051840175	4.26675761607331	-1.52377615916125
H	1.26715353505725	3.84040972119679	1.68768287147566
H	1.27729917943244	5.30747958540590	0.66754691416787
H	2.16428468863516	3.84942220171023	0.14559103001389
H	0.86652761503000	3.93661828139281	-2.08177364035667
H	-0.02721962567463	5.36784479291868	-1.50164451215777
H	-0.91363277123080	3.92835435508961	-2.07283184800960
H	-2.18851464388844	3.82944649563989	0.16875968164991
H	-1.30998784560933	5.29760084094338	0.67676696232839
H	-1.27486644643972	3.83395405859385	1.70115228140805
C	-4.14645491662550	-0.15221323248638	0.00898967900192
C	-4.77766001893222	-0.81080163204902	1.24535690988755
C	-4.78291656509023	-0.70616993185716	-1.27360311148685
C	-4.33219386189584	1.38215708804552	0.07307785017645
H	-4.62123226823119	-1.90129906294262	1.23627767338816
H	-5.86222998143438	-0.61985985146838	1.25091081665908
H	-4.34858281900758	-0.40924745244499	2.17387105021835
H	-3.86752224934344	1.87205312814892	-0.79696051523638
H	-3.87903808962412	1.79678653991962	0.98648170057838
H	-5.40655756927789	1.62523095702144	0.07539183068569
H	-4.62589648072460	-1.79326103086918	-1.35699070075273
H	-4.36062166604713	-0.22887410225412	-2.16895913056443

H -5.86756924577069 -0.51634074885676 -1.25710272219666

C7-6 [O_3N] adduct

83

C	-2.59053581493187	-1.39552902413163	0.40935925901364
O	-1.84059285347499	-2.41623342361553	0.38017519885655
Rh	0.10926935561724	-2.21896934078945	-0.23617543820949
Rh	-0.33895308805154	0.11061956319620	-0.63099159652355
N	-0.79342287790252	-0.41176683505504	-2.52415920656754
C	-0.73576419670163	-1.65303385447764	-2.93786433436945
O	-0.43164576498698	-2.63153548692758	-2.16067162696171
C	-1.02824507075183	-2.00910037097708	-4.37229982130530
H	-1.31230432963098	-1.13025266698558	-4.96634682116299
H	-1.84223700554928	-2.74995159585491	-4.40127739370596
H	-0.13708161203330	-2.47983163463400	-4.81627269728956
H	-0.97098181597248	0.29650651034791	-3.23517453266212
O	-2.25598997128667	-0.24325228619522	-0.00643818611782
O	1.63367869423420	0.32062460327042	-1.16701405746747
C	2.40541861812136	-0.68689818220463	-1.11709303143963
O	2.04165407710125	-1.86339907539566	-0.81768261365248
O	0.22034214975773	0.42850351121474	1.35573443382827
C	0.59286840901160	-0.53951019891717	2.07839423005282
O	0.64405682512701	-1.74868406760910	1.69114846650683
C	3.88170726840609	-0.43960117150585	-1.44748847157052
C	4.72204903266912	-1.69176153283441	-1.16784634826878
C	4.37255850980390	0.73433126730309	-0.57795671724696
C	3.96802692254769	-0.06913562068588	-2.94199064301036
H	5.77639816368625	-1.48779030186145	-1.41231664991048
H	4.38459688845407	-2.54510419863133	-1.77388731049672
H	4.66340537470669	-1.98257713719757	-0.10799878595904
H	3.78846404288488	1.64300258084447	-0.77360826409451

H	5.43042954184547	0.94562010209988	-0.80021239150329
H	4.28825354196438	0.49058820579357	0.49368296970841
H	5.01361555833943	0.14577829937782	-3.21354707127451
H	3.36244671649966	0.82050134721825	-3.16295370552108
H	3.61128001539643	-0.89737390427460	-3.57496696132962
C	0.97837955135727	-0.21711894067231	3.52689604999817
C	-0.32694257501658	0.14535724564232	4.26695758101899
C	1.93276936959042	0.99155208975586	3.52313387572661
C	1.65187460501662	-1.41979866338418	4.19878765892638
H	-0.10268706522304	0.40066807395766	5.31494378891476
H	-1.03069306033077	-0.70214265646103	4.26157746103233
H	-0.81860385911759	1.00796785341639	3.79484623730319
H	0.98393763319986	-2.29305302567116	4.22316368481134
H	1.92035509236516	-1.15985654120102	5.23496546369763
H	2.57135895558312	-1.70875832335940	3.66663893152617
H	1.45497901446054	1.87096022874841	3.07048867416206
H	2.85152220537371	0.76673523459556	2.95686497407662
H	2.21962376000926	1.24137574609205	4.55694227338177
C	-3.97295662383028	-1.52227606152959	1.05577566383268
C	-4.43486862269999	-2.98560502569687	1.06679732213085
C	-4.98884894322823	-0.64861311157558	0.30191738561125
C	-3.80856706359792	-1.00538969534249	2.50415695043383
H	-4.52354366567434	-3.38154101217240	0.04296739840934
H	-5.42193257653775	-3.05645748358468	1.54986884780184
H	-3.73075402257140	-3.62296417362901	1.61982011587126
H	-4.77434588742827	-1.06617777398020	3.03022295926878
H	-3.46934527845487	0.04160256713291	2.51334926751509
H	-3.07369262044820	-1.61074194241057	3.05760105566028
H	-5.97796943122019	-0.74057771486174	0.77728333630273
H	-5.08216677929865	-0.96496461689267	-0.74923080464356

H	-4.69394799639671	0.40989295964080	0.31593384883987
C	-0.99514382023545	2.52959060049357	-0.95935440655181
C	-0.95774636232125	3.07089543608059	0.43182015280598
C	0.13256588298476	3.81309361001930	0.91722554074227
C	0.13351055009204	4.28329899916061	2.23361184121880
C	-0.94166944732266	4.02703870080701	3.08734759306782
C	-2.02596605937982	3.28209178185397	2.61123360140431
C	-2.03294679805721	2.80422925378323	1.30217477120231
H	0.99256739909402	4.85701772754507	2.59054245607578
H	-0.93291280393524	4.39615451150707	4.11565033052845
H	-2.87202443498002	3.05917412538892	3.26587943789634
H	-2.87229009097096	2.19313443282661	0.96871462333111
H	0.98167554175550	4.01726590807140	0.27129916738095
C	-0.09240264654749	2.78027919789779	-2.11888476706935
O	-0.40399958359285	2.49681935783361	-3.26217807812022
O	1.07124215461402	3.31813223896043	-1.77572373898437
C	2.00395966789225	3.59134960254600	-2.85152146721511
C	3.16469350555380	4.33407404752767	-2.27524711790840
H	2.30626042746960	2.64040411456104	-3.31251251581863
H	1.47604868992538	4.19053949747657	-3.61093107583933
C	4.43678569884523	3.95948655474130	-2.43490436980361
H	2.91916228738817	5.25021513917547	-1.72457025225850
H	4.69831574816074	3.04568114444595	-2.97779477077593
H	5.26056790752089	4.55504989345771	-2.03120842280442
N	-2.22521089755733	2.22783687055029	-1.41918836577203
N	-3.24270193717766	1.94199793682666	-1.79309004868827

C7-6 [O_4] adduct

83

C	-1.67965906201651	2.34856993222500	-1.22832693788342
O	-1.33541108306260	1.18086191440178	-1.58984917451288

Rh	0.11947555505199	0.17510501457396	-0.56275005179251
Rh	0.35830461102177	2.12705738988841	0.82576583416480
N	1.72100755297908	2.75736572192073	-0.52978472797418
C	2.05491836738781	2.05071527143918	-1.57944323025576
O	1.52277582263775	0.90865136476701	-1.84827078896063
C	3.10290471372619	2.52752657028743	-2.55080813547632
H	3.47580045818501	3.52921916747491	-2.29829345105846
H	2.67850853142530	2.53729425265734	-3.56642552185741
H	3.94288501124729	1.81495673161036	-2.55114114530964
H	2.21735633133837	3.64005756056367	-0.42707481069348
O	-1.16804196759550	2.98301039518877	-0.25882765829383
O	1.82298086548761	1.13983623161755	1.86963714548468
C	2.11330691309436	-0.05099499343211	1.54486509711684
O	1.59965844516206	-0.68231568352683	0.57195696689779
O	-1.07215842759265	1.30816855087972	2.10457380073202
C	-1.59982716514772	0.20112054181532	1.80025881945522
O	-1.29106826952221	-0.49175643890747	0.77915276095450
C	3.14452726079037	-0.81408197936424	2.38294947162529
C	4.28036743460038	-1.26977933640569	1.44599564034874
C	2.42964088005766	-2.04405840083080	2.98130783453232
C	3.70318432402898	0.06959546307714	3.50445047721463
H	3.89613694196353	-1.91151382877745	0.64157564426617
H	5.02908378378992	-1.83838578941517	2.02022826373090
H	4.78545778717490	-0.40319705717410	0.98936581180743
H	2.04725194466121	-2.70098205593824	2.18754211161327
H	1.58301779433565	-1.73797660109649	3.61743352191323
H	3.13539220514473	-2.61811898755583	3.60240296825564
H	2.90624401149862	0.40339938110142	4.18541165688659
H	4.20076021538769	0.96328418706549	3.09893701265015
H	4.44137196529599	-0.50149312314041	4.08934610982443

C	-2.71140455698162	-0.36560458579327	2.69079102526722
C	-4.00745089435708	-0.36506197255113	1.85208289013235
C	-2.89931592863512	0.49179683138979	3.94783529639857
C	-2.33196684126655	-1.80654755953827	3.08017129503641
H	-4.83863913065842	-0.77623853140920	2.44652573195462
H	-3.89009696405020	-0.97553480954196	0.94586537392587
H	-4.27683365070382	0.65804637555633	1.54356685081337
H	-2.19825645457145	-2.43413256914464	2.18947007138824
H	-3.12714539757451	-2.24838405647741	3.70117075291711
H	-1.39398788286835	-1.82397997620018	3.65882443846119
H	-3.70038827929544	0.06164583187850	4.56949886949833
H	-3.17815812893693	1.52411628881963	3.69037893792051
H	-1.97789891198285	0.52753485898358	4.54868012605491
C	-2.79231409438493	3.00916237777965	-2.04858359964555
C	-3.15243467407198	4.38495990033244	-1.47594078503381
C	-4.02161424736779	2.07840159758388	-2.01882115702510
C	-2.27818482896996	3.15270272320027	-3.49638509061588
H	-3.95016332123030	4.83882694970615	-2.08468655738769
H	-2.28436808933366	5.06091212158895	-1.48317228689207
H	-3.51169170491883	4.30421415615595	-0.43898058922968
H	-2.03084639663963	2.17088605433346	-3.92521858634457
H	-1.37656017567714	3.78531984656664	-3.53526794430713
H	-3.05431741560336	3.62232920927104	-4.12119504292271
H	-3.77999940255537	1.09223710504912	-2.43986454943315
H	-4.83833612220220	2.52295093979613	-2.60934007182211
H	-4.38189055655346	1.93434377330106	-0.98720864415639
C	-0.14293338854115	-1.79386134588086	-2.06218290219326
C	1.10359187330853	-2.57034900531924	-1.78751367162414
C	2.25632640493634	-2.35982658533850	-2.56780017565068
C	3.43793808897481	-3.05198425036405	-2.29827927118294

C	3.49502954125557	-3.97269577449304	-1.24789896126710
C	2.35677533428106	-4.18010465873340	-0.46412102628239
C	1.17514157674731	-3.48224414900519	-0.71886888538146
H	4.31854254787320	-2.86745938658982	-2.91866151005001
H	4.41978283467542	-4.51481988488446	-1.03649467784659
H	2.38736642079673	-4.88414370831537	0.37130923989642
H	0.31113861992614	-3.63670634142265	-0.07931370264885
H	2.24613856489171	-1.64371141192488	-3.39093910609868
C	-1.56201293299980	-2.19183830655881	-1.79352626901800
O	-2.50624934739807	-1.69275277770328	-2.37001241077111
O	-1.66825366926650	-3.14188761564773	-0.87001582453644
C	-3.00227973620413	-3.62947784457079	-0.59052348280638
C	-2.87550221719578	-4.83736841786494	0.27906160048783
H	-3.48251533707789	-3.87208086655839	-1.55246342354896
H	-3.58508058030671	-2.83171154133798	-0.10818552547866
C	-3.53604423802191	-4.99487945619644	1.42921773684946
H	-2.21864818120196	-5.62945299566382	-0.10036019347276
H	-4.19144908952206	-4.21037623464721	1.82155822902538
H	-3.44991471499658	-5.91468064711487	2.01470408399688
N	-0.11654914843256	-1.02220785876022	-3.16795799979014
N	-0.10241892764381	-0.34370318273094	-4.05933994097340

TS1[O_3N]

83

C	-2.59183884889501	-1.39467058181340	0.43169965596210
O	-1.86380734109808	-2.42515238852161	0.37545850416892
Rh	0.08997336053567	-2.25542525009771	-0.25182373528205
Rh	-0.32320679996634	0.13312630925234	-0.59277541766299
N	-0.79660636563343	-0.34964935942472	-2.49229226445452
C	-0.76187956013884	-1.58400725863068	-2.93644294959309
O	-0.47354038429127	-2.58851558696960	-2.19402620675536

C	-1.06974678770553	-1.88608293262825	-4.38177007808812
H	-1.34614188821111	-0.98484123002948	-4.94532169860760
H	-1.89372219181160	-2.61476377488597	-4.42945632542710
H	-0.18797795020991	-2.35324401351859	-4.84773258599189
H	-0.96738638553226	0.37577742406442	-3.18644868800647
O	-2.24417058695949	-0.23856472016111	0.02901297600733
O	1.65887812218776	0.28519929868154	-1.14331056985592
C	2.40747369983785	-0.74276880442618	-1.11748101149820
O	2.02533684920125	-1.91520616542303	-0.83944162418524
O	0.28183778263536	0.37233999244372	1.40110144218489
C	0.62801693337175	-0.62713541571965	2.09721647759334
O	0.64179478118909	-1.82592562757060	1.68634687179572
C	3.88786222710238	-0.51630047866265	-1.45189399282386
C	4.69950681668799	-1.79801034558046	-1.22716650346687
C	4.41423359472312	0.61012841927774	-0.54131681138358
C	3.97512120716293	-0.09100185040541	-2.93110704267257
H	5.75627577069187	-1.61131984606614	-1.47549026277562
H	4.33456331820119	-2.61994960565703	-1.86015702786019
H	4.64396328033896	-2.12626937873493	-0.17821929882095
H	3.85567766880209	1.54145469795964	-0.70117008594848
H	5.47700215253514	0.80027426667437	-0.75979735377544
H	4.32675501923580	0.32915923485401	0.52100054585998
H	5.02402395685632	0.10813347893059	-3.20210805042639
H	3.39073277141491	0.82083274028272	-3.11320336648256
H	3.59351294511726	-0.88483123508988	-3.59310800531404
C	1.02313218174510	-0.34732083776781	3.55351511060661
C	-0.28079959829387	-0.03435400139357	4.31869262021040
C	1.95716113947341	0.87596397702535	3.59062934570908
C	1.72066487916813	-1.56370473913870	4.17495366394893
H	-0.05131490669066	0.17871570219811	5.37499414005442

H	-0.97514198080476	-0.88857436485711	4.28222239723876
H	-0.78624517716486	0.84233485424489	3.88845099848681
H	1.06599211286875	-2.44700237787370	4.17071443960276
H	1.99455569505423	-1.33951956416540	5.21808570067164
H	2.63965491399967	-1.81910941626457	3.62464239653485
H	1.45811750994791	1.76653365886769	3.18341039280275
H	2.87068801166151	0.69312779667579	3.00154093815949
H	2.25505948933988	1.08569249385001	4.63019874033974
C	-3.96422174620156	-1.51571029285604	1.10347429686364
C	-4.56353627039960	-2.90414855804118	0.83311341422997
C	-4.91542428090806	-0.41843990637621	0.60548046927511
C	-3.70542562973917	-1.33470922552842	2.61737085523915
H	-4.72799121604877	-3.06067334572139	-0.24495764447630
H	-5.53444290261043	-2.99422936688225	1.34502399906280
H	-3.90141175616991	-3.70122228057226	1.19875759725736
H	-4.65723303170936	-1.40930163518509	3.16671554239211
H	-3.26110153364218	-0.34934354966935	2.82773162097376
H	-3.02218052841649	-2.10975684838639	2.99716718456675
H	-5.89175610243264	-0.52578482303519	1.10364646896256
H	-5.07374422588356	-0.49035992085132	-0.48172387022180
H	-4.52183571332904	0.58350453458625	0.82648953189926
C	-0.70916765239732	2.21532055669696	-0.81817029086397
C	-0.91797234230792	3.06142457836183	0.37364504677532
C	-0.43825799102626	4.38727374545528	0.44762228731054
C	-0.62560933259952	5.14268546220676	1.60514522019166
C	-1.32633341875578	4.60518162373860	2.69057212768247
C	-1.83792232442296	3.30378743138071	2.61597023532172
C	-1.62692335919288	2.53407643882402	1.47513300879145
H	-0.23212520172152	6.16074497055432	1.65540428484989
H	-1.47874607617786	5.20305217793418	3.59284901293046

H	-2.39425077838068	2.88338096934524	3.45700892818726
H	-2.01338501552168	1.51842181191835	1.41826629064056
H	0.09955485688944	4.82238632369504	-0.39349941591474
C	0.05397263815142	2.69858411338313	-2.01102727241430
O	-0.31237040248798	2.57522215647167	-3.16445402717930
O	1.23950002223998	3.18528696460418	-1.65107750860655
C	2.14070558593435	3.56868083016703	-2.71991707093072
C	3.32319429242110	4.24261311616444	-2.10533613819981
H	2.42310313594324	2.67155000914951	-3.28902396978517
H	1.59269786058474	4.24609941497755	-3.39456073716379
C	4.58680090192833	3.87334570432331	-2.33102411805114
H	3.10142417199155	5.10176070441320	-1.46076637428109
H	4.82453165305301	3.01294545299879	-2.96474462869298
H	5.42695347782183	4.41827631926593	-1.89112351317426
N	-2.33030203291463	2.15483375134592	-1.55121895381936
N	-3.36476916924142	1.77737739733773	-1.64946829040794

TS1[O_4]

83

C	-1.65583161578443	2.30986298215528	-1.28672100247345
O	-1.29586280903966	1.13311044107585	-1.60838918099251
Rh	0.14398563451555	0.15584034215973	-0.53001149525301
Rh	0.36377400597058	2.19809572700074	0.80392702986569
N	1.74212532467850	2.78145485277701	-0.55609714428634
C	2.07647873136134	2.04792306204581	-1.58343638060573
O	1.55252385197811	0.89118558117627	-1.81870255305560
C	3.11475709773897	2.49792184700541	-2.57834421214493
H	3.49202778137244	3.50471819359890	-2.35388586929749
H	2.67865459284520	2.48639942732004	-3.58915601821521
H	3.95391602190888	1.78435508114095	-2.57364863726502
H	2.23941617752618	3.66718547997980	-0.48638994272052

O	-1.15321694347150	2.99050094079940	-0.34848157011970
O	1.81060531952270	1.23200122221951	1.90557673782734
C	2.08058959369292	0.02292880355476	1.65370265557137
O	1.57858399636208	-0.65580828325581	0.70342464903015
O	-1.08374996432880	1.42183508110844	2.08992009559059
C	-1.61145465395470	0.30918604034749	1.81863623320210
O	-1.29714916809829	-0.42755859391086	0.82870757944450
C	3.07584463862414	-0.71674550641547	2.55688575789735
C	4.23313736943919	-1.23427955394084	1.68067720944334
C	2.32716347541477	-1.90584421933872	3.19459028049947
C	3.61648108813241	0.21319376231052	3.64975680885304
H	3.86683408581971	-1.91942971532198	0.90341706722224
H	4.96116173149385	-1.77292243279937	2.30790734053151
H	4.75795596114244	-0.40063087576020	1.18649695738263
H	1.95981390668921	-2.59830591977756	2.42358133849965
H	1.46485845585127	-1.55838426210298	3.78674746166149
H	3.00548101366082	-2.45615691576127	3.86572636076058
H	2.80547417809214	0.59387934818754	4.28806366398492
H	4.14078858992421	1.07754347830712	3.21541306677801
H	4.32703382737353	-0.33939177485891	4.28453114072572
C	-2.73455065724465	-0.22019042960582	2.72039385538886
C	-4.01956125215096	-0.27513724452764	1.86770875746535
C	-2.94532383011846	0.70059046933393	3.92820493456097
C	-2.34894423414349	-1.63417852372802	3.19341300615804
H	-4.85572933932501	-0.65894826107464	2.47350798445564
H	-3.88721491263579	-0.93320680611599	0.99757103215684
H	-4.29167257065537	0.72758828900003	1.50000053582445
H	-2.19669774532128	-2.30823656383630	2.34124029321325
H	-3.14830688944333	-2.04890253686269	3.82779257650472
H	-1.41864200183300	-1.61131635466125	3.78423819825030

H	-3.75361175430750	0.29960764654564	4.56019898262294
H	-3.22433279173976	1.71709487585065	3.61414745824991
H	-2.03285739131578	0.77197841207395	4.53927361419835
C	-2.78739036509050	2.91406490020531	-2.12708824150176
C	-3.16820452642123	4.30642899089167	-1.61040592525889
C	-3.99962215757371	1.96438301451768	-2.04326414354676
C	-2.29081295341626	3.00462087382536	-3.58456125428183
H	-3.97872929234108	4.71982579230355	-2.23107922439967
H	-2.31270585585563	4.99689850618434	-1.65287659261740
H	-3.51806560104273	4.26277578721238	-0.56805483362170
H	-2.03307357734399	2.00979566473628	-3.97557032341432
H	-1.39942294938917	3.64845040140363	-3.65892857269063
H	-3.07978850961262	3.43623450325788	-4.22074714623912
H	-3.74559034615500	0.96555243629733	-2.42563342180011
H	-4.83177303754793	2.36938440531237	-2.64072859157737
H	-4.34449969944658	1.85718956718023	-1.00193060677397
C	-0.04431983101789	-1.63386168002292	-1.67041715937473
C	1.08727787136614	-2.57401085474046	-1.81812384901724
C	2.41190732669181	-2.09471191912365	-1.73516455025137
C	3.49040279574807	-2.96845966362531	-1.86208068603242
C	3.26871916724959	-4.33460943150371	-2.06836249332994
C	1.96025578224002	-4.82390717112384	-2.16617828458109
C	0.87909786307403	-3.95185131520763	-2.05552750145702
H	4.51020934687930	-2.58261924519263	-1.79326480042599
H	4.11591748991712	-5.01941232622428	-2.15898425163286
H	1.78320532922299	-5.88840971881637	-2.33702366069254
H	-0.13327212106872	-4.34634631880349	-2.14364624009011
H	2.57812792468857	-1.03167231417481	-1.57736773094568
C	-1.43844526326110	-2.17582117869403	-1.54808479385130
O	-2.39465178522289	-1.83314932748552	-2.20946374633178

O	-1.49449762399163	-3.05915480645800	-0.54798221394478
C	-2.78475959975099	-3.65590617413824	-0.27679995668723
C	-2.56970686202356	-4.82939602880118	0.62181830734614
H	-3.22451534198050	-3.96135367552524	-1.24019855781643
H	-3.44514370489832	-2.90211139047539	0.17613696414372
C	-3.24173540618924	-5.02778350452040	1.75891671820376
H	-1.83301932788292	-5.56401648467768	0.27463698368084
H	-3.97606196893720	-4.30054342819980	2.12088723681007
H	-3.08636721419654	-5.92552725941895	2.36408248422098
N	-0.18833577593960	-0.90160294508859	-3.29151529240819
N	0.09444387430456	-0.17405329470429	-4.07379070521009

8[O$_3$N]

C	-2.48912703135947	-1.28864370172808	0.51478710370416
O	-1.81949811270045	-2.35034443615123	0.38073018617804
Rh	0.14386546702445	-2.24446295144206	-0.23390379942505
Rh	-0.14586674688816	0.19335758532148	-0.40558583960381
N	-0.67271173888146	-0.16579477495539	-2.31956365653649
C	-0.69806316440088	-1.36581028843004	-2.84923476349386
O	-0.42625304164230	-2.43079743357692	-2.19173336468984
C	-1.05962259522395	-1.54748885271756	-4.30228556183561
H	-1.29293553839986	-0.59488142587369	-4.79626297618764
H	-1.92770248516812	-2.22082803792983	-4.37558267881765
H	-0.21904417359422	-2.03326232453293	-4.82195262152780
H	-0.87272423085141	0.62161460847307	-2.93345663968443
O	-2.07912957535443	-0.12303153001916	0.20817463322398
O	1.82025746895981	0.28369472161160	-0.98773732023166
C	2.51222708394725	-0.78150324134430	-1.08116762367109
O	2.09150032061695	-1.94548870441410	-0.83372306759110
O	0.45811440577757	0.26605276409467	1.59850943273312
C	0.73504049370318	-0.80070188029769	2.22940207131918

O	0.70161561546767	-1.96231900327987	1.73195900313270
C	3.95951415544217	-0.59875754848304	-1.55398662935654
C	4.74311771421485	-1.90996408380459	-1.42189979482788
C	4.61789502270946	0.50471933445631	-0.70440979800476
C	3.90083294808980	-0.16138670925635	-3.03323626475795
H	5.77648042817388	-1.75765470736572	-1.77138078497792
H	4.28807486682547	-2.71115960081667	-2.02219820882658
H	4.77900799508884	-2.24823796487266	-0.37502048771072
H	4.08788928033174	1.45972616417895	-0.81815040167109
H	5.66214500456624	0.64677797938664	-1.02486827489358
H	4.62032401654212	0.23154410591076	0.36341007975151
H	4.92117553167111	0.00278878535458	-3.41436280600054
H	3.33385575537000	0.77376469453940	-3.14329349384581
H	3.42092044652249	-0.93454317864873	-3.65447447100628
C	1.13585364419105	-0.63589594144459	3.70086505558377
C	-0.02760042085704	0.06344808789689	4.43254936579166
C	2.39631135603389	0.25132932342553	3.74892876337964
C	1.42216344288045	-1.99676497003784	4.34601062158783
H	0.22641206555760	0.19481008419648	5.49632456902466
H	-0.94995656173169	-0.53603512107338	4.37055549245406
H	-0.23029098947301	1.05280153953354	3.99787109150703
H	0.53656796269795	-2.64894497311605	4.31255070276396
H	1.70547553872003	-1.85198818230991	5.40057616116068
H	2.24602801700502	-2.51672020299491	3.83484796764462
H	2.19704164055049	1.24117916102635	3.31348278101067
H	3.22691949777496	-0.21182941319952	3.19169948051688
H	2.71749205075440	0.38675794812119	4.79393669070985
C	-3.87663685899666	-1.37723292065210	1.16148415593260
C	-4.48118934395605	-2.77100041857862	0.93968251515042
C	-4.80533878265562	-0.29580611743282	0.58770775169597

C	-3.65481664848190	-1.13035880467422	2.67177904680017
H	-4.61065946349922	-2.98047722284731	-0.13388703358833
H	-5.46963422545687	-2.82486109695613	1.42228001696174
H	-3.84193596467925	-3.55626247334431	1.36675456832485
H	-4.61652652120963	-1.20565695135704	3.20354258594021
H	-3.23783862369293	-0.12776305009560	2.85247334702897
H	-2.96404298067997	-1.87525251554834	3.09700710924461
H	-5.79310477036935	-0.36422092516223	1.07003518240994
H	-4.94384735061021	-0.42677051612658	-0.49734790376817
H	-4.40114406347431	0.71078260444241	0.76358631738653
C	-0.37986094162333	2.16162788046218	-0.52504006437922
C	-1.07394872212933	3.02053261764084	0.38000511967398
C	-1.20213992218891	4.41665221536765	0.11374577420249
C	-1.86945567296024	5.24929229332251	1.00021260665303
C	-2.41957814535668	4.71731507368953	2.17750362821437
C	-2.30247414222386	3.35048481241353	2.46552094752400
C	-1.64268816139210	2.50669081748346	1.58092829181803
H	-1.96439741386635	6.31569877139038	0.78457965035718
H	-2.94181067810323	5.37655912211868	2.87578651269330
H	-2.72965493024520	2.94615316361919	3.38584481479727
H	-1.54434815364348	1.44760298029611	1.79805191016035
H	-0.77397680320346	4.83662486882236	-0.79873029009978
C	0.23462534110328	2.75434525259643	-1.73497728526424
O	-0.28904363013349	2.68855089528429	-2.83340442813741
O	1.42687330352232	3.29223473289317	-1.49635229241391
C	2.18171475580535	3.75629472615259	-2.64837929780731
C	3.49423292422493	4.27345885716388	-2.15853550467335
H	2.30217579963096	2.92227501681614	-3.35515855493908
H	1.59201690415825	4.54602791686697	-3.14208216739889
C	4.67510028683220	3.82552234706679	-2.59339839425118

H	3.44330777411335	5.07858708655829	-1.41555731889449
H	4.74231153055304	3.01548240820845	-3.32710400584773
H	5.61498887970346	4.25272133098874	-2.23213801220927

8[O_4]

C	-1.62998636712404	2.07598553495596	-1.27401363273524
O	-1.21019244308022	0.90253929222301	-1.53221296099791
Rh	0.25855214881366	0.03657379342479	-0.40165198534728
Rh	0.39620686550712	2.18658510177624	0.79841553722103
N	1.78220248879767	2.71156696594076	-0.57460430514000
C	2.15717412424230	1.91632063170640	-1.53999825870890
O	1.67052437750045	0.73117066382832	-1.70841104649606
C	3.20174399090938	2.32556306916641	-2.54634179317051
H	3.57820797680193	3.34036862118098	-2.35997087274284
H	2.77056274664017	2.27556934471787	-3.55830807933545
H	4.03920616508228	1.61141572844524	-2.50916980037521
H	2.25297834580718	3.61435257862118	-0.56149443907499
O	-1.12691377971800	2.85629761131420	-0.41877459362303
O	1.84914938435259	1.33220171051291	1.98635949079777
C	2.16749935081490	0.12401727208085	1.81321172811738
O	1.69936476651539	-0.63374631997541	0.90203798235715
O	-1.04459609725687	1.43666615677943	2.12279061092588
C	-1.51086327537427	0.28257045584745	1.92952566873261
O	-1.16930376190758	-0.49534411833240	0.97947528435647
C	3.18176000889698	-0.52557336696871	2.76261335719100
C	4.36544197304158	-1.04352230007132	1.92194507078597
C	2.47558334581884	-1.70658958820294	3.46111689675490
C	3.67501326028295	0.48432976011879	3.80562352457138
H	4.03155763833228	-1.78492087317812	1.18203494971955
H	5.10940235343819	-1.51856405989984	2.58088813313242
H	4.86031056605285	-0.21781652116907	1.38561070841852

H	2.14081421787613	-2.45453477235875	2.72804866172441
H	1.59648283076588	-1.36135143766374	4.02922725650541
H	3.17053946775963	-2.19150835322434	4.16478766771858
H	2.84267952656762	0.86863492334260	4.41355843320698
H	4.17225049169251	1.34232397664290	3.32893213253805
H	4.39740663506944	-0.00549801434225	4.47728401354681
C	-2.59534275595384	-0.26146852110109	2.86943853198998
C	-3.91004917906453	-0.30535318726034	2.06133634449812
C	-2.75801203342476	0.64602608905830	4.09449882403086
C	-2.20013477036917	-1.68377474957864	3.30879643626162
H	-4.72101902211021	-0.71166973209516	2.68642165547444
H	-3.80201945459672	-0.94350569088078	1.17315931979879
H	-4.20209574862204	0.70377598223083	1.72932372841309
H	-2.12202152758894	-2.35685720643150	2.44457937672760
H	-2.96202632373043	-2.08622781267861	3.99516401884883
H	-1.23182501855091	-1.67901865205506	3.83559859453818
H	-3.54178510349954	0.23914056524521	4.75296281270901
H	-3.04666354910180	1.66615308195604	3.80254137204717
H	-1.82225757478779	0.70864753131143	4.67104174958582
C	-2.86401409923237	2.52349888790114	-2.06684182233749
C	-3.23943729048066	3.96924725067867	-1.72241214728764
C	-4.01589577469251	1.56801085106354	-1.68674834833371
C	-2.55378983620245	2.39335620269484	-3.57063046764839
H	-4.13401835649165	4.26304639518037	-2.29389037017258
H	-2.42487230070014	4.66489151471905	-1.97489931794051
H	-3.46081914711300	4.07980252984654	-0.65061227252881
H	-2.30135087371799	1.35560279236213	-3.83132843643440
H	-1.70901993799368	3.04011717659741	-3.85810976091956
H	-3.43403025964237	2.69768412431779	-4.15885561743210
H	-3.76236080830646	0.52907867935219	-1.94221139572524

H	-4.92995506670914	1.85250736788968	-2.23172664668625
H	-4.23010411040017	1.61611606474268	-0.60705026623333
C	0.13982932135778	-1.70682570647912	-1.35571419955592
C	1.21934353410145	-2.52187125975864	-1.81445586478466
C	2.57281562657148	-2.13707038853957	-1.59079776761917
C	3.61589411836834	-2.94850880875924	-2.01935458346225
C	3.34322085121877	-4.15186314489038	-2.68409403849130
C	2.01858163112929	-4.55232740975609	-2.92201347625819
C	0.96977696996421	-3.75282165825207	-2.49235965457718
H	4.64967564157089	-2.64562500451351	-1.83886075048342
H	4.16814098663734	-4.78514605035623	-3.02090137264169
H	1.81528152336577	-5.49097597409608	-3.44201778235104
H	-0.05795661978185	-4.07056873210926	-2.67877241415327
H	2.77464827252092	-1.19965511196008	-1.08160479814065
C	-1.24234265202391	-2.16451840368303	-1.63914998967091
O	-1.85144747631588	-1.82575696067711	-2.63378762246899
O	-1.72731571769414	-2.94454432820350	-0.67421440115875
C	-3.11374098234907	-3.35690336888198	-0.78920909123479
C	-3.38019489883842	-4.36377016207242	0.28174756410194
H	-3.26206737053492	-3.78371560560982	-1.79420856410471
H	-3.75759924570727	-2.46973024552819	-0.69064120334429
C	-4.32284647301038	-4.22031508963937	1.21721971998180
H	-2.75576950491064	-5.26500916334314	0.25473735351855
H	-4.94650641647024	-3.32141686983478	1.26380994159620
H	-4.50429081810510	-4.99520679436238	1.96758189328432

TS2[O$_3$N]

81

C	-2.51931021173917	-1.41819102408673	0.27350520558957
O	-1.93345646207747	-2.52883376873800	0.11440757048248
Rh	0.00783421873800	-2.55475214596219	-0.57353645246000

Rh	-0.15060867231642	-0.12138101350711	-0.81617000129283
N	-0.80429028009653	-0.52254602673777	-2.68664230788559
C	-0.93438006359303	-1.73705372928296	-3.16139678182881
O	-0.61973609553981	-2.79016706425568	-2.49934404130499
C	-1.48023221947158	-1.96266891800956	-4.54973159025463
H	-1.69099288413462	-1.02019207109547	-5.07274374169139
H	-2.40498303273685	-2.55685210389792	-4.48023711683543
H	-0.75335029406641	-2.55005563275215	-5.13177038547074
H	-1.13857248741483	0.23280757863033	-3.28411351953275
O	-2.07277796924619	-0.29568691394704	-0.11506928643255
O	1.80805290153148	-0.15625838723996	-1.46181774434029
C	2.44431411963837	-1.25786548882293	-1.50949820022394
O	1.95926508984183	-2.38669910340187	-1.21898616960223
O	0.53122282287063	-0.04002825887140	1.17644148635801
C	0.78752463047182	-1.09955755410838	1.82146390679285
O	0.64380528779243	-2.27160203132733	1.36702817345292
C	3.91334814753946	-1.17315357629893	-1.94286613005670
C	4.56289011376531	-2.56229230135383	-1.93196653483889
C	4.64200165403475	-0.24202876561881	-0.95242622182857
C	3.96304959205414	-0.57748609432166	-3.36412171763658
H	5.61479735378236	-2.47979579232064	-2.24769827571567
H	4.04727310733367	-3.24888349587850	-2.61959896138461
H	4.53699357217005	-3.00691243164085	-0.92593790827853
H	4.22381017910789	0.77440903763161	-0.97909661133959
H	5.71049932316705	-0.18438178524980	-1.21338586090561
H	4.55939330962613	-0.62063171537971	0.07915204873224
H	5.00986676831478	-0.49519065065752	-3.69682951342840
H	3.51004928736895	0.42367002698503	-3.39255747206620
H	3.42510579504566	-1.22002331540441	-4.07950069986299
C	1.36079470980360	-0.92474605167420	3.23300664464270

C	0.58283745774694	0.18264149796485	3.96533582836922
C	2.83619850436191	-0.50220375865744	3.05405949833174
C	1.28009227562124	-2.23812168471230	4.02110607773649
H	0.99090746959834	0.31613971696684	4.97965221853670
H	-0.48406212647038	-0.07762456748691	4.05534513485829
H	0.65726648620671	1.14082322340245	3.43209793047164
H	0.23637317336744	-2.57306248671257	4.12711485387689
H	1.69942172807339	-2.09197080309654	5.02913343850506
H	1.84494902804184	-3.03910071507326	3.52313009184116
H	2.91044909479527	0.44237680681487	2.49420955001855
H	3.40332592050882	-1.27326202596084	2.50821215622611
H	3.30707119958720	-0.36049678251631	4.03988779410183
C	-3.82173530730605	-1.39433999855450	1.08232667740124
C	-4.54155609954764	-2.74643297485444	0.99310768115957
C	-4.73872958514710	-0.26311327629166	0.59254908827187
C	-3.38457571906072	-1.12045410278380	2.54138450884441
H	-4.82117363949842	-2.97903788575031	-0.04671938549363
H	-5.46226853343321	-2.71685113267541	1.59709579397548
H	-3.90704032789278	-3.56219955380194	1.36702635370949
H	-4.26860954760796	-1.09903105510217	3.19832176858202
H	-2.86925921041170	-0.15160045310255	2.61964568430424
H	-2.70134762358002	-1.90551017148919	2.90206775927901
H	-5.65333015234449	-0.23708851710051	1.20590201240139
H	-5.03477708703721	-0.41955304269446	-0.45714633612780
H	-4.23963156034330	0.71252752873002	0.66837464963549
C	-0.34567326513455	2.04774438232907	-0.95569848267311
C	-1.36342468219330	2.72613765771380	-0.13128632490529
C	-2.11781341260389	3.81451744745256	-0.62313176707216
C	-3.10394296367343	4.41158188563590	0.16153422674196
C	-3.34004975750687	3.95338497826458	1.46309026937514

C	-2.58524558133188	2.89044146362029	1.97274247873311
C	-1.61406094677763	2.27833868905063	1.18356491128619
H	-3.68559784396665	5.24419408626198	-0.24166447253605
H	-4.10871976158756	4.42661860511209	2.07951760780457
H	-2.76261577502142	2.52850733562507	2.98835215139048
H	-1.03536938552953	1.44410461874596	1.57528024723352
H	-1.94365188792868	4.18615812652349	-1.63361345497307
C	-0.29901418815762	2.36535005194744	-2.41078215594744
O	-1.30262622871920	2.22919377500054	-3.09016804950720
O	0.85216527806227	2.68890475001639	-3.01928404822180
C	1.97273340795289	3.00906197323622	-2.17314069569898
C	1.44936735764431	3.35077081250604	-0.81398405077981
H	2.63888576007999	2.13761411949271	-2.13593568261228
H	2.48602445623186	3.85904603351436	-2.64335008227606
C	1.50002482343485	2.43951074242474	0.19857534945508
H	0.98407411188658	4.33054879601595	-0.66913039436304
H	2.04676638316077	1.50558312613785	0.08115061624223
H	1.09776697188509	2.66426332650680	1.18617918493529

TS2[O_4]

81

C	-2.53469992876550	-1.46160535285467	0.25851940211969
O	-1.93118251024396	-2.56888562157945	0.15646148494650
Rh	0.01214570286361	-2.59953280981794	-0.53009276483405
Rh	-0.14271094621146	-0.16246621303475	-0.77611993945638
O	-0.70589381673181	-0.44944147702422	-2.72064295627876
C	-0.82450740668649	-1.64898382642042	-3.17376061858848
N	-0.59904413891115	-2.71319683159670	-2.44859849635216
C	-1.24307513626256	-1.75257924709065	-4.61864187267958
H	-0.48730019040482	-1.25940963558003	-5.25006677212622
H	-2.19207810388135	-1.21300784182156	-4.76119173609615

H	-1.36178435628576	-2.79590529068432	-4.94093567870040
O	-2.08521027121139	-0.34578598258856	-0.14700796714040
O	1.82900164026840	-0.21378224997412	-1.38231140521115
C	2.46434125228492	-1.31654881774457	-1.42789906225583
O	1.97481611988984	-2.44491007042853	-1.14591408811396
O	0.51128730075136	-0.05434106097564	1.18727209582741
C	0.79553573454351	-1.10096657526452	1.85469416293165
O	0.65836563963523	-2.28066185057400	1.43510127843866
C	3.92901846171764	-1.23575629778084	-1.87634401564496
C	4.62993688489739	-2.58651640368562	-1.68479251035438
C	4.63859917601404	-0.15047263240710	-1.04430395198970
C	3.93392237823854	-0.84462925127954	-3.36904443479752
H	5.67833047311574	-2.50703909838778	-2.01268013997533
H	4.14220461988784	-3.37788678144679	-2.27221956503044
H	4.62076530757863	-2.89340144356118	-0.62777218454636
H	4.18485076082106	0.83741027830232	-1.20596526350869
H	5.70012016854052	-0.09596729917858	-1.33276723735239
H	4.58618794564955	-0.38112369967933	0.03208378755945
H	4.97169328707176	-0.77891149796529	-3.73258382254108
H	3.44947899056611	0.13014138609268	-3.52672121822755
H	3.40442653951942	-1.59686128356921	-3.97548876142062
C	1.37328979273655	-0.86357126767497	3.25640088460957
C	0.41744135557453	0.05816710107084	4.03747682274996
C	2.73922108479072	-0.16901922632864	3.07149364217884
C	1.55360061209054	-2.19134541104627	4.00195399493733
H	0.81057157874410	0.22897867805870	5.05219301145247
H	-0.58310116153104	-0.39469418874332	4.12795561269696
H	0.30959596767143	1.03200632882353	3.53911737166854
H	0.59175638172883	-2.71154150406739	4.12722558591832
H	1.97564523181739	-1.99960077163777	5.00109035909519

H	2.23496986411900	-2.86406193813293	3.46082212251693
H	2.62323289886245	0.79975104967096	2.56396720660449
H	3.42202947150095	-0.79331037780438	2.47240548060764
H	3.20629185950274	0.00559454270375	4.05382228351327
C	-3.87161943211739	-1.43300549391206	1.00905278706051
C	-4.61839476823084	-2.76180882088429	0.82875537597998
C	-4.73825688641399	-0.26050732873014	0.52565389521683
C	-3.50058635681821	-1.22956775755275	2.49729489845223
H	-4.86341182196330	-2.93774724475137	-0.23089254539131
H	-5.56064963201487	-2.73824170422308	1.39873251047387
H	-4.01656181861738	-3.60888579824479	1.18661401555156
H	-4.41673165676883	-1.19290738935163	3.10795051561344
H	-2.95216446817701	-0.28583099155785	2.63922850557944
H	-2.87045190308311	-2.05478166750635	2.86389205748117
H	-5.68499352351354	-0.24593709843921	1.08845205455487
H	-4.97720384378993	-0.35847604173244	-0.54525100622118
H	-4.22738190271583	0.70034037993876	0.67662271108791
C	-0.42088975504506	1.99436695535808	-0.92446972658313
C	-1.34697818063301	2.70232010831879	-0.01462210939117
C	-2.02115957386443	3.88035904066903	-0.40867673666224
C	-2.91741484103184	4.51364295782439	0.45086069519599
C	-3.15029660428891	3.99475947177509	1.73048069697092
C	-2.47682141983560	2.84012344421727	2.14342812088108
C	-1.58696445701311	2.20075970228399	1.28155311833593
H	-3.43410132355658	5.41912421299967	0.12318019588307
H	-3.85226449956358	4.49296419280772	2.40424378567401
H	-2.64949253413117	2.42995926769721	3.14147617452779
H	-1.06493027626795	1.30326774692976	1.60226961922627
H	-1.85110363094820	4.30008959982386	-1.40105508575560
C	-0.57939187848886	2.29247703533233	-2.38532053633285

O	-1.67653576421347	2.24060710912496	-2.89610941966740
O	0.50159956092946	2.52991003492309	-3.15267621549243
C	1.72996215731779	2.79695753093038	-2.45035769551919
C	1.37465626052636	3.28080668719884	-1.07935969159770
H	2.32282633580411	1.87464162294565	-2.40434002713443
H	2.26135756618880	3.56378080867181	-3.03100791580699
C	1.51246462391965	2.47704003282402	0.01418406111155
H	0.94386156931577	4.28285522937795	-0.98679104074675
H	2.03056990449883	1.52193063834555	-0.05409640500243
H	1.21042810536003	2.81949775428109	1.00480124064139
H	-0.73480984662182	-3.59834246300457	-2.93250100534535

6

25

C	-0.23085067164565	2.08767719463819	-0.69919642197808
C	0.38287599435929	3.23780270699863	0.00248663096378
C	1.60633711951959	3.07526865373645	0.68246912535340
C	2.21307235923970	4.15334671178521	1.32853855238278
C	1.60782027930143	5.41430277735223	1.31765093124799
C	0.39326755054372	5.58467744773598	0.64527062878677
C	-0.21478913417577	4.51327018668290	-0.01235215832578
H	3.16112494206873	4.00263050045923	1.85125811954292
H	2.07934796741679	6.25751353372944	1.82838986738260
H	-0.08749246914232	6.56623129368004	0.62331237232243
H	-1.15589405680991	4.66326129857924	-0.53859337757975
H	2.08631792969732	2.09403440813290	0.71461285362565
C	-1.64875187810409	1.79108534074999	-0.96459370648740
O	-2.03524398646684	0.80907729646398	-1.57057267676570
O	-2.46494130327039	2.72186384103751	-0.45286300468681
C	-3.88234850888801	2.52757905126636	-0.65206705304442
C	-4.59843330197979	3.58513343636581	0.12455007022908

N	0.57027216368653	1.13338588376609	-1.13041611672435
N	1.25513054470072	0.31322122069767	-1.50291328545397
H	-4.14582703447926	1.51755615454838	-0.29652498409311
H	-4.11166306369868	2.57918730184402	-1.72790824352265
C	-5.47688030695961	4.43708068676787	-0.41091804571042
H	-4.37671047409255	3.61778060378706	1.19829814613546
H	-5.70711217982119	4.42082112699317	-1.48200209718521
H	-6.00363587179966	5.17389079580138	0.20247037578478

7

23

C	-2.14147761163386	-5.58161780962729	-0.70141750808943
C	-1.53379713427413	-5.21519452829116	-2.01439897245749
C	-2.33590270364321	-4.64308482004047	-3.01620236942175
C	-1.78602243351164	-4.29703357069415	-4.25253091294565
C	-0.42568135784356	-4.51320119124860	-4.50356176537090
C	0.37886227677243	-5.08116534640946	-3.51133057016042
C	-0.17365243477067	-5.43365975825650	-2.27516404606714
H	-2.42132670476293	-3.85322069463563	-5.02358355469462
H	0.00474196844177	-4.23905716864980	-5.47024417271871
H	1.44146641230309	-5.25584775223714	-3.69925755725279
H	0.45859956779465	-5.88617437390682	-1.50779297797027
H	-3.39679034861236	-4.47409227544397	-2.82116651175573
C	-3.25213799340312	-6.59735732547531	-0.67756343595979
O	-4.06189036548499	-6.84239738337208	-1.53579788052447
O	-3.24596752270018	-7.25436423534835	0.50712156069296
C	-2.06154784539542	-6.96434880178554	1.28050789617165
C	-1.40858220905938	-5.77262195178358	0.60828809531078
H	-2.37663884503857	-6.76893553476525	2.31641221668505
H	-1.40983643100826	-7.85165443441515	1.25955875719969
C	-2.27124492021963	-4.56581236072969	0.43470863717385

H	-0.32408889760159	-5.66641899445808	0.67414366774992
H	-3.22050410035346	-4.49983247484160	0.97653666063182
H	-1.76858057009496	-3.61290201088423	0.25316191917351

N_2

N	0.00000000000000	0.00000000000000	-0.20013880414973
N	0.00000000000000	0.00000000000000	0.90013880414973

References

[1] F. Neese, *WIREs Computational Molecular Science* **2022**, *12*, e1606.
[2] F. P. Caló, A. Zimmer, G. Bistoni, A. Fürstner, *J. Am. Chem. Soc.* **2022**, *144*, 7465-7478.
[3] P.-P. Chen, P. Wipf, K. N. Houk, *Nat. Commun.* **2022**, *13*, 7292.
[4] P. Pracht, F. Bohle, S. Grimme, *Physical Chemistry Chemical Physics* **2020**, *22*, 7169-7192.
[5] C. Bannwarth, S. Ehlert, S. Grimme, *J. Chem. Theory Comput.* **2019**, *15*, 1652-1671.
[6] A. D. Becke, *The Journal of Chemical Physics* **1992**, *96*, 2155-2160.
[7] C. Lee, W. Yang, R. G. Parr, *Physical Review B* **1988**, *37*, 785-789.
[8] S. H. Vosko, L. Wilk, M. Nusair, *Canadian Journal of Physics* **1980**, *58*, 1200-1211.
[9] P. J. Stephens, F. J. Devlin, C. F. Chabalowski, M. J. Frisch, *J. Phys. Chem.* **1994**, *98*, 11623-11627.
[10] S. Grimme, J. Antony, S. Ehrlich, H. Krieg, *J. Chem. Phys.* **2010**, *132*, 154104.
[11] F. Weigend, R. Ahlrichs, *Phys. Chem. Chem. Phys.* **2005**, *7*, 3297.
[12] V. Barone, M. Cossi, *J. Phys. Chem. A* **1998**, *102*, 1995-2001.
[13] M. Cossi, N. Rega, G. Scalmani, V. Barone, *Journal of Computational Chemistry* **2003**, *24*, 669-681.
[14] K. Eichkorn, O. Treutler, H. Öhm, M. Häser, R. Ahlrichs, *Chem. Phys. Lett.* **1995**, *240*, 283-290.
[15] F. Neese, *Journal of Computational Chemistry* **2003**, *24*, 1740-1747.
[16] F. Weigend, *Physical Chemistry Chemical Physics* **2006**, *8*, 1057-1065.
[17] G. Henkelman, H. Jónsson, *J. Chem. Phys.* **2000**, *113*, 9978.
[18] G. Henkelman, B. P. Uberuaga, H. Jónsson, *J. Chem. Phys.* **2000**, *113*, 9901.
[19] J. Tao, J. P. Perdew, V. N. Staroverov, G. E. Scuseria, *Phys. Rev. Lett.* **2003**, *91*, 146401.
[20] V. N. Staroverov, G. E. Scuseria, J. Tao, J. P. Perdew, *The Journal of Chemical Physics* **2003**, *119*, 12129-12137.
[21] E. v. Lenthe, E. J. Baerends, J. G. Snijders, *The Journal of Chemical Physics* **1993**, *99*, 4597-4610.
[22] C. van Wüllen, *The Journal of Chemical Physics* **1998**, *109*, 392-399.
[23] S. K. Wolff, T. Ziegler, *The Journal of Chemical Physics* **1998**, *109*, 895-905.
[24] G. te Velde, F. M. Bickelhaupt, E. J. Baerends, C. Fonseca Guerra, S. J. A. van Gisbergen, J. G. Snijders, T. Ziegler, *Journal of Computational Chemistry* **2001**, *22*, 931-967.
[25] C. Fonseca Guerra, J. G. Snijders, G. te Velde, E. J. Baerends, *Theoretical Chemistry Accounts* **1998**, *99*, 391-403.
[26] C. Adamo, V. Barone, *The Journal of Chemical Physics* **1999**, *110*, 6158-6170.
[27] E. Van Lenthe, E. J. Baerends, *Journal of Computational Chemistry* **2003**, *24*, 1142-1156.
[28] C. C. Pye, T. Ziegler, *Theoretical Chemistry Accounts* **1999**, *101*, 396-408.
